# Loss of the E3 ubiquitin ligase MKRN1 represses diet-induced metabolic syndrome through AMPK activation

**DOI:** 10.1038/s41467-018-05721-4

**Published:** 2018-08-24

**Authors:** Min-Sik Lee, Hyun-Ji Han, Su Yeon Han, Il Young Kim, Sehyun Chae, Choong-Sil Lee, Sung Eun Kim, Seul Gi Yoon, Jun-Won Park, Jung-Hoon Kim, Soyeon Shin, Manhyung Jeong, Aram Ko, Ho-Young Lee, Kyoung-Jin Oh, Yun-Hee Lee, Kwang-Hee Bae, Seung-Hoi Koo, Jea-woo Kim, Je Kyung Seong, Daehee Hwang, Jaewhan Song

**Affiliations:** 1 Harvard Medical School, Boston Children’s Hospital, 3 Blackfan Circle CLS-16060.2, Boston, MA 02115 USA; 2 Department of Biochemistry, College of Life Science and Biotechnology, Yonsei University, Seoul, 03722 Republic of Korea; 3 Laboratory of Developmental Biology and Genomics, Research Institute for Veterinary Science and BK21 Program for Creative Veterinary Science and Research, College of Veterinary Medicine, Seoul National University, Seoul, 08826 Republic of Korea; 4 Korea Mouse Phenotyping Center (KMPC), Seoul National University, Seoul, 08826 Republic of Korea; 5 Center for Plant Aging Research, Institute for Basic Science, and Department of New Biology, Daegu Gyeongbuk Institute of Science and Technology (DGIST), Daegu, 42988 Republic of Korea; 6 Department of Nuclear Medicine, Seoul National University Bundang Hospital, Seongnam, 13620 Republic of Korea; 7 Metabolic Regulation Research Center, Korea Research Institute of Bioscience and Biotechnology (KRIBB), Daejeon, 34141 Republic of Korea; 8 College of Pharmacy, Yonsei Institute of Pharmaceutical Sciences, Yonsei University, Incheon, 21983 Republic of Korea; 9 Division of Life Sciences, College of Life Sciences & Biotechnology, Korea University, Seoul, 02841 Republic of Korea; 10 Department of Biochemistry and Molecular Biology, Brain Korea 21 PLUS Project for Medical Science, Yonsei University College of Medicine, Seoul, 03722 Republic of Korea

## Abstract

AMP-activated protein kinase (AMPK) plays a key role in controlling energy metabolism in response to physiological and nutritional status. Although AMPK activation has been proposed as a promising molecular target for treating obesity and its related comorbidities, the use of pharmacological AMPK activators has been met with contradictory therapeutic challenges. Here we show a regulatory mechanism for AMPK through its ubiquitination and degradation by the E3 ubiquitin ligase makorin ring finger protein 1 (MKRN1). MKRN1 depletion promotes glucose consumption and suppresses lipid accumulation due to AMPK stabilisation and activation. Accordingly, *MKRN1*-null mice show chronic AMPK activation in both liver and adipose tissue, resulting in significant suppression of diet-induced metabolic syndrome. We demonstrate also its therapeutic effect by administering shRNA targeting MKRN1 into obese mice that reverses non-alcoholic fatty liver disease. We suggest that ubiquitin-dependent AMPK degradation represents a target therapeutic strategy for metabolic disorders.

## Abstract (web summary)

AMPK activation has been suggested as treatment for obesity and its complications. Here the authors show that the ubiquitin ligase MKRN1 binds to AMPK and mediates its ubiquitination and degradation. Loss of MKRN1 leads to AMPK activation, increased glucose consumption and decreased lipid accumulation.

## Introduction

Metabolic syndrome is associated with the risk of developing a number of disorders, including type 2 diabetes (T2D), non-alcoholic fatty liver disease (NAFLD) and cardiovascular disease, and has become a common pathological condition due to the increased prevalence of obesity associated with the western lifestyle^1–3^. The syndrome develops in response to chronic disturbances in the balance between energy intake and expenditure^4^. From a therapeutic perspective, cellular energy expenditure (EE) has emerged as an attractive strategy for the treatment of obesity and diabetes^1^. Although the molecular mechanisms implicated in regulating the energy-wasting process in brown fat and skeletal muscle have been identified, new and effective drugs for the treatment of obesity that stimulate cellular EE remain to be developed^2^.

AMP-activated protein kinase (AMPK), a cellular energy-sensing enzyme, is a key regulator of metabolic syndrome and comprises diverse heterotrimeric complexes containing a catalytic (α1 or α2) subunit and two other regulatory subunits (β1 or β2 and γ1, γ2 or γ3)^5,6^. Under various conditions of energy stress that elevate the AMP/ATP ratio, the binding of AMP to the γ subunit maintains the enzyme in an activated state due to allosteric activation, which promotes phosphorylation by the upstream kinases liver kinase B1 and calcium calmodulin-dependent protein kinase kinase β^6^. Consequently, AMPK activation generates ATP to restore energy homeostasis by upregulating catabolic processes and inhibiting energy-consuming anabolic processes^5–7^.

AMPK, an attractive anti-obesity target for exploiting cellular EE, integrates nutritional, hormonal and pharmacological inputs to maintain the cellular energy balance^8,9^. In many genetic rodent models, including leptin-deficient (*ob*/*ob*) mice, sustained decreases in AMPK activity accompany metabolic syndrome^10,11^. AMPK activation mitigates metabolic syndrome in peripheral tissues. AMPK activation in the liver promotes the phosphorylation of acetyl-CoA carboxylase (ACC) to restrain fatty acid synthesis^5,7^ and suppresses the activity of the transcription factors sterol regulatory element-binding protein 1 (SREBP-1) and carbohydrate response element-binding protein (ChREBP)^12–14^, which inhibit hepatic lipogenesis. AMPKα2 deficiency causes hypertriglyceridemia^15^, whereas liver-specific AMPK activation prevents diet-induced NAFLD in mice^16^. Pharmacological AMPK activators, such as 5-aminoimidazole-4-carboxamide (AICA) riboside, ameliorate insulin resistance in rodent models^5,17,18^, although poor bioavailability limits their biological effects in vivo. Furthermore, a lack of both AMPKβ1 and β2 in adipocytes opposes the thermogenic responses of brown and beige fat and intensifies diet-induced NAFLD and glucose intolerance^19^, supporting the physiological role of AMPK in lipid metabolism and its clinical relevance. In contrast, the activation of hypothalamic AMPK is associated with the promotion of appetite in response to fasting and results in increased food intake and body weight^20–22^. Given the role of AMPK in the maintenance of the whole-body energy balance, it is not surprising that AMPK activation increases the orexigenic effect related to the energy supply^23^. Thus, despite a wide array of salutary effects of AMPK activation on metabolic syndrome, the systemic effect of chronic AMPK activation presents a unique challenge^24^. The identification of a tissue-specific AMPK regulatory mechanism would be an alternative strategy for the development of potent and selective AMPK activators^25,26^.

Although AMPK phosphorylation is the best-delineated system through which AMPK enzymatic activity is regulated, the ubiquitin-proteasome system has begun to attract attention as a possible regulatory mechanism that controls AMPK function in energy metabolism^27–29^. Tripartite motif-containing 28, an E3 ubiquitin ligase, and UBE2O, an E2 ubiquitin-conjugating enzyme, ubiquitinate and degrade AMPKα1 and α2, respectively, resulting in AMPK dysfunction in cancer^30,31^. However, an E3 ubiquitin ligase for AMPK that controls systemic metabolism has yet to be reported.

As shown in this study, makorin ring finger protein 1 (MKRN1) is an E3 ubiquitin ligase for AMPK in the liver and adipocyte tissues. AMPK activation in mice lacking MKRN1 prevents NAFLD, insulin resistance and obesity associated with a high-fat diet (HFD). The possible curative effects of MKRN1 inhibition on hepatic steatosis and hyperglycaemia were further observed using an adenoviral gene delivery system. Our study provides new therapeutic opportunities for treating metabolic syndrome by hijacking the E3 ubiquitin ligase for AMPK.

## Results

### MKRN1 inhibition induces AMPK-dependent catabolic processes

**Fig. 1:**
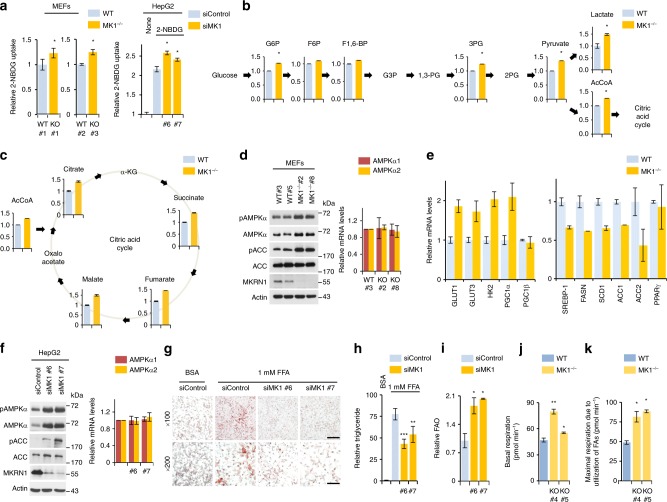
MKRN1 depletion stimulates glucose metabolism by activating AMPK signalling. Analysis of wild-type (WT) or *MKRN1*-knockout (*MK1*^−*/*−^) littermate primary mouse embryonic fibroblasts (MEFs) and HepG2 cells transduced with two independent siRNAs targeting MKRN1 (siMK1 #6 and siMK1 #7) or a control siRNA for 48 h. **a** Glucose consumption in *MK1*^−*/*−^ MEFs (left) or MKRN1-depleted HepG2 cells (right) was measured based on the absorption of 2-[*N*-(7-nitrobenz-2-oxa-1,3-diazol-4-yl) amino]-2-deoxy-d-glucose (2-NBDG) by the cells. **b**, **c** Intracellular levels of glycolytic and citric acid cycle intermediates were determined by capillary electrophoresis–time-of-flight mass spectrometry of the MEFs. Each bar represents the relative amount of a metabolite for WT MEFs. G6P glucose-6-phosphate, F6P fructose-6-phosphate, F1,6-BP fructose-1,6-bisphosphate, G3P glucose-3-phosphate, 1,3-BG 1,3-bisphosphoglycerate, 3PG 3-phosphoglycerate, 2PG 2-phosphoglycerate, AcCoA acetyl-CoA, α-KG α-ketoglutarate. **d**, **f** To validate the AMPK signalling pathway or mRNA levels of AMPKα subunits, immunoblotting or quantitative real-time PCR analysis was performed using MEFs (**d**) and HepG2 cells (**f**). Cell lysates were immunoblotted with antibodies against pAMPKα, AMPKα, pACC, ACC, MKRN1 and actin. **e** The mRNA levels of glycolytic or lipogenic enzymes in MEFs were analysed by quantitative real-time PCR. **g**, **h** FFA-induced steatosis in HepG2 cells. Oil Red O staining (**g**) and TG levels (scale bar = 100 µm (top), 50 µm (bottom) (**h**) of the cells treated with FFA/FFA-free bovine serum albumin (BSA), which served as controls. All the experiments with MEFs were conducted in cells within the first 3–6 passages. **i** Fatty acid oxidation was analysed in MKRN1-depleted HepG2 cells. **j** The basal oxygen consumption rate (OCR) was measured in WT and *MK1*^−/−^ MEFs. **k** After sequential treatment with oligomycin, FCCP and rotenone/antimycin-A in the presence of BSA or BSA conjugated to palmitate, OCR was measured in WT or *MK1*^−*/−*^ MEFs. The results were normalised against total protein levels using XF-Analyze. All data are presented as the mean ± standard deviation (s.d.) of triplicate samples and are representative of at least three independent experiments. two-tailed Student’s *t*-test; ^*^P ≤ 0.05, ^**^P ≤ 0.01, ^***^P ≤ 0.001

The physiological role of MKRN1 has been well delineated in cancer based on its ability to degrade several tumour suppressors^32–35^. MKRN1 is also associated with adipocyte differentiation in vitro^36^. However, the implications of MKRN1 for systemic energy metabolism have yet to be studied. We initially measured glucose metabolic activity in *MKRN1*-deficient mouse embryonic fibroblasts (MEFs) to further define the metabolic functions of MKRN1. Intriguingly, increased glucose uptake was observed in MEFs lacking *MKRN1* or HepG2 cells depleted of MKRN1 (Fig. 1a, f and Supplementary Fig. 1a). Accordingly, we observed increased levels of metabolites of glycolysis and the tricarboxylic acid cycle (Fig. 1b, c) in MEFs lacking *MKRN1*. The data suggest that MKRN1 depletion promotes energy production via cellular glucose utilisation. In addition, our previous reports indicated that MKRN1 suppression might promote AMPKα1 and α2 phosphorylation^35^. Based on these observations, we reasoned that the activation of AMPK signalling might be involved in promoting glucose metabolism in *MKRN1*-null MEFs. As expected, MKRN1 deficiency increased the levels of activated AMPKα (phosphorylated at T172), which suppressed the activity of its downstream target, acetyl coenzyme A ACC, via phosphorylation (Fig. 1d). Additionally, MKRN1 ablation induced an increase in AMPKα protein level without affecting messenger RNA levels, suggesting that these events depend on post-translational processes (Fig. 1d). Similar results were also observed in MKRN1-depleted HepG2 and Hep3B cells following transfection with two independent small interfering RNAs (siRNAs) (Fig. 1f and Supplementary Fig. 1b). In accordance with AMPK activation under MKRN1 depletion, alterations in the expression of genes that activate glycolysis and suppress fatty acid synthesis were further observed (Fig. 1e)^7,37,38^. AMPKα ablation completely reversed the effects of MKRN1 on glycolytic and lipogenic gene expression (Supplementary Fig. 1c, d). The implications of MKRN1 for the modulation of AMPK-mediated regulation of glucose metabolism were further revealed by the increased glucose uptake *MKRN1*-null MEFs, which was reversed by AMPKα knockdown (Supplementary Fig. 1e, f). Given the suppression of lipogenic gene expression, we further investigated the effect of MKRN1 ablation on HepG2 cellular steatosis induced by free fatty acids (FFAs). MKRN1 knockdown reduced lipid accumulation in HepG2 cells, as indicated by Oil Red O staining (Fig. 1g) and the triglyceride (TG) mass (Fig. 1h). These effects were completely reversed upon AMPKα2 ablation (Supplementary Fig. 1g). Corroborating these findings, fatty acid oxidation (FAO) was enhanced in MEFs lacking *MKRN1* or HepG2 cells in which MKRN1 was ablated (Fig. 1i–k and Supplementary. Fig. h–j). Thus, MKRN1 deficiency induces activation of the AMPK signalling pathway, leading to a metabolic switch from anabolism to catabolism.

### MKRN1 acts as an E3 ubiquitin ligase for AMPKα

**Fig. 2:**
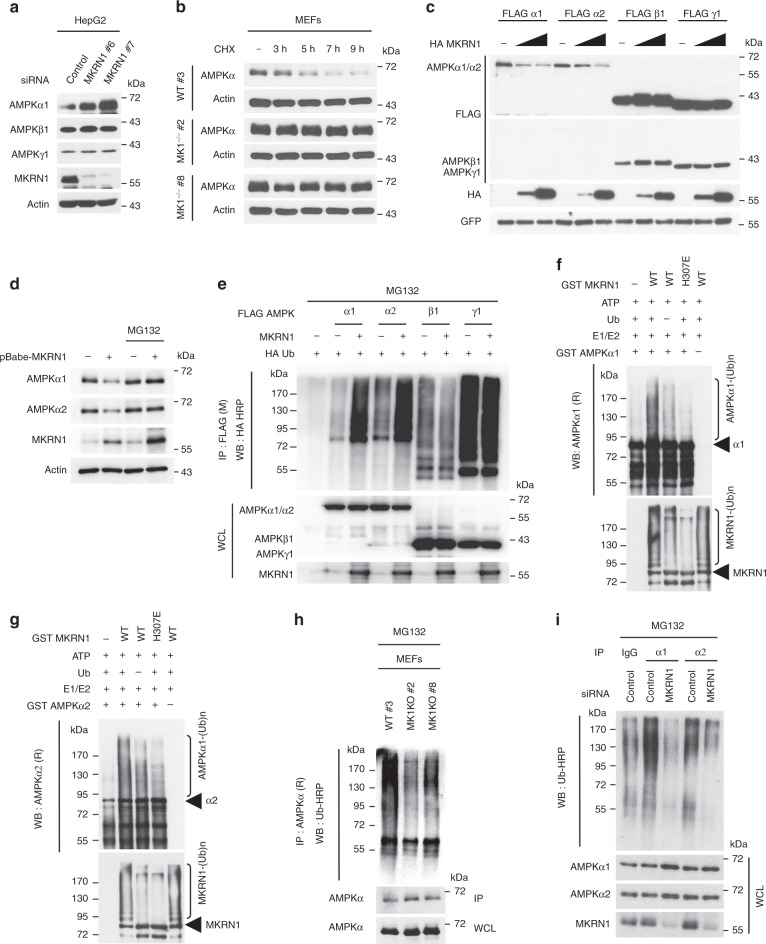
The E3 ubiquitin ligase MKRN1 ubiquitinates and degrades AMPKα subunits. **a** MKRN1 knockdown increases AMPKα1 protein levels. The HepG2 cells were transfected with MKRN1 siRNAs (#6 and #7). **b** MKRN1 knockout stabilises AMPKα. WT or *MK1*^*−/−*^ MEFs were treated with CHX (100 mg ml^−1^) at the indicated time points. **c**, **d** MKRN1 expression promotes the proteasomal degradation of AMPKα subunits. The protein levels of ectopically expressed AMPK subunits were analysed using HEK293T cells. GFP was used as a transfection control (**c**). HepG2 cells were infected with retrovirus expressing MKRN1, followed by selection using puromycin. The cells were treated with 20 µM of MG132 for 6 h, and AMPKα1, α2, MKRN1 and actin were detected with the indicated antibodies (**d**). **e** MKRN1 induces both AMPKα1 and α2 ubiquitination. Constructs expressing FLAG/AMPKα1, α2, β1, γ1, 3.1/MKRN1 and HA/Ub were transfected into 293T cells. The ubiquitination assay was performed using cell lysates under denaturing conditions (in 1% SDS buffer). **f**, **g** MKRN1 directly ubiquitinates AMPKα subunits. In vitro ubiquitination of AMPKα1 (**f**) and α2 (**g**). **h**, **i** MKRN1 is required for the ubiquitination of AMPKα. Ubiquitinated endogenous AMPKα was determined under denaturing conditions using MG132-treated MEFs (**h**) and HepG2 cells (**i**). All the experiments with MEFs were conducted in cells within the first 3–6 passages. The data are representative of at least three independent experiments

As MKRN1 depletion induced AMPKα stabilisation without affecting its mRNA levels, the potential role of MKRN1 as an E3 ligase for AMPKα was further investigated. The results of co-immunoprecipitation analyses using overexpressed proteins showed that both the N and C termini of MKRN1 bound to the β-subunit-interacting domain of AMPKα (Supplementary Fig. 2a, b, d, e). Recombinant MKRN1 directly interacted with AMPKα (Supplementary Fig. 2c). Endogenous interactions between AMPK and MKRN1 were further observed in MEFs (Supplementary Fig. 2f). MKRN1 knockdown resulted in increased AMPKα protein levels, without affecting AMPKβ and γ, indicating that the α subunits are specific targets of MKRN1 (Fig. 2a). In addition to these observations, the AMPKα protein displayed an extensively prolonged half-life in cycloheximide (CHX)-treated *MKRN1*-null MEFs compared with that of the control (Fig. 2b). Conversely, MKRN1 overexpression promoted the degradation of the AMPKα1 and α2 proteins, but not of the β and γ proteins (Fig. 2c). Further confirming these findings, AMPKα stabilised by MKRN1 depletion was degraded upon overexpression of MKRN1 resistant to MKRN1-targeting siRNA but was not affected by the expression of MKRN1 H307E, an E3 ligase-defective MKRN1 mutant^32^ (Supplementary Fig. 3a). Furthermore, the levels of the AMPKα1 and α2 proteins were rescued by the addition of an MG132 proteasome inhibitor (Fig. 2d), suggesting that MKRN1 degrades AMPKα in a proteasome-dependent manner.

Consistent with these results, MKRN1 promoted the ubiquitination of both AMPKα1 and α2 predominantly through K48-linked polyubiquitination (Fig. 2e and Supplementary Fig. 3b, c). In vitro ubiquitination reactions using recombinant proteins revealed that MKRN1 directly facilitated the ubiquitination of AMPKα1 and α2, but not of AMPKβ and γ (Fig. 2f, g and Supplementary Fig. 3d). In contrast to wild-type (WT) MKRN1, the enzymatically inert H307E MKRN1 mutant failed to promote AMPK ubiquitination, despite its ability to bind to AMPK (Fig. 2f, g and Supplementary Fig. 2b). Notably, *MKRN1*-null MEFs and MKRN1-depleted HepG2 cells displayed decreased ubiquitination of endogenous AMPKα compared with that of the control, indicating that MKRN1 might be a pivotal E3 ligase for AMPKα (Fig. 2h, i). Based on these results, both AMPKα1 and α2 are direct and robust targets of the MKRN1 E3 ubiquitin ligase.

### MKRN1 deficiency reduces HFD-induced obesity

**Fig. 3:**
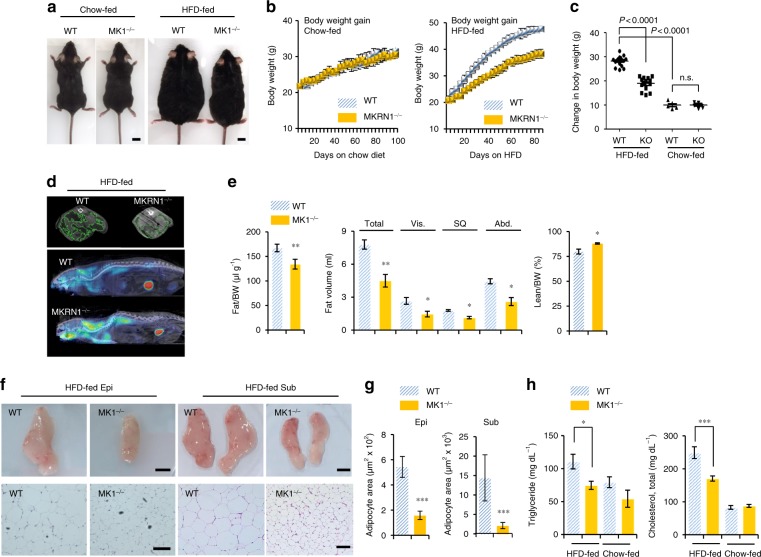
A lack of MKRN1 expression reduces obesity in mice placed on an HFD. Male WT and *MK1*^*−/−*^ mice fed a chow diet or an HFD for 16 weeks. **a** Representative images of male WT and *MK1*^*−/−*^ mice fed a chow diet (left) or an HFD (right). **b** The body weights of male mice on a chow diet (left) or on an HFD (right) were measured every 4 days. **c** Body weight gain in male mice fed a chow diet or an HFD for 12 weeks (chow, WT *n* = 10 and *MK1*^*−/−*^ *n* = 12; HFD, WT *n* = 16 and *MK1*^*−/−*^ *n* = 18). **d**, **e** Fat volume (Vis visceral, SQ subcutaneous, Abd abdominal adipose tissue) and lean mass weight were calculated (**e**) through micro-CT imaging (**d**) of WT and *MK1*^*−/−*^ mice on an HFD (*n* = 5 mice per group). *P*-value compared with WT. **f**, **g** Representative images of epididymal fat (left), subcutaneous fat (right) (top, scale bar = 1 cm) and H&E staining (bottom, scale bar = 50 (Epi) or 100 (Sub) µm) (**f**) and quantitative analysis of the adipocyte area (**g**) in male WT and *MK1*^*−/−*^ mice fed an HFD (*n* *=* 5 mice per group). *P*-value compared with WT. **h** Plasma concentrations of TG and cholesterol in 24 h-fasted male mice fed an HFD (*n* = 6 mice per group). The images in **a**, **d** and **f** are representative images from the respective experiments. Two-tailed Student’s *t*-test; ^*^P ≤ 0.05, ^**^P ≤ 0.01, ^***^P ≤ 0.001, n.s. not significant. Mean ± s.d.

**Fig. 4:**
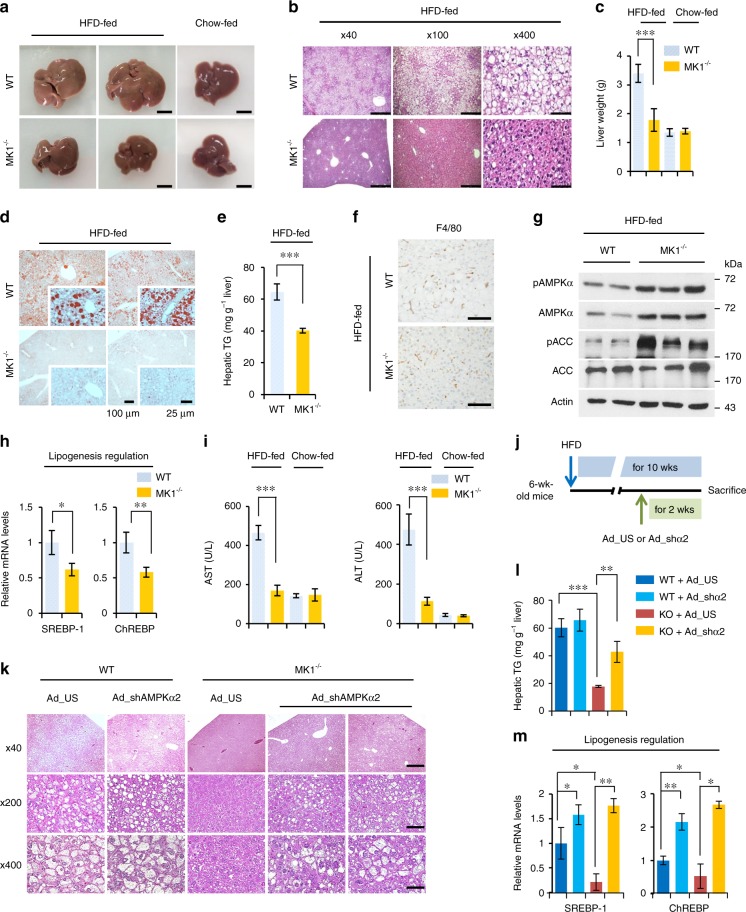
Effect of MKRN1 deficiency on hepatic AMPK signalling and diet-induced NAFLD. Livers from male WT and *MK1*^*−/−*^ mice fed a chow diet or an HFD for 16 weeks were analysed. **a** Representative livers from mice on an HFD (upper left; fatty livers of HFD-fed WT mice) or a chow diet (upper right; normal livers of chow-fed WT mice). Scale bar = 1 cm. **b** H&E staining of livers. Scale bar = left, 250 µm; middle, 100 µm; and right, 25 µm. **c** Liver weights (*n* = 9 HFD-fed mice, *n* = 6 chow-fed mice per group). **d** Representative images of Oil Red O-stained livers (*n* = 6 mice per group). Scale bar = 100 and 25 µm. **e** Liver TG contents (WT *n* = 6 and *MK1*^*−/−*^ *n* = 7). **f** Representative images of immunohistochemical staining for macrophage antigens (F4/80) in liver sections. Scale bar = 100 µm. **g** Hepatic AMPK signalling in WT or MK1^−/−^ mice. **h** Relative mRNA levels of genes related to hepatic lipogenesis (WT *n* = 5 and *MK1*^*−/−*^ *n* = 6). **i** ALT and AST serum levels (WT *n* = 7 and *MK1*^*−/−*^ *n* = 8). **j** Schedule of AMPKα2 knockdown. WT and *MKRN1*-null mice were injected with Ad_US (as control) or Ad_shα2 (shRNA targeting AMPKα2) via the tail vein. **k**–**m** Hepatic steatosis induced by MKRN1 deficiency was rescued by the ablation of AMPKα2 using adenovirus. **k** Representative image of H&E staining. Scale bar = up, 50 µm; middle, 100 µm; and bottom, 500 µm. **l** Plasma TG contents were measured. **m** Lipogenic enzymes were analysed by quantitative real-time PCR in livers from WT and *MK1*^*−/−*^ mice infected with adenoviruses. The data in **c**, **e**, **h**, **i**, **l** and **m** are presented as the mean ± s.d. Two-tailed Student’s *t*-test; ^*^P ≤ 0.05, ^**^P ≤ 0.01, ^***^P ≤ 0.001, n.s. not significant

The finding that the metabolic functions of MKRN1 are implicated in regulating AMPK activity and thus in cellular metabolic homeostasis, regardless of nutritional and pharmacological cues, led us to explore the physiological roles of MKRN1 in *MKRN1*-null mice, which do not exhibit apparent developmental deficits or overt phenotypes^39^. We first compared the body weights of male *MKRN1*-null mice to WT mice that were fed standard chow or an HFD to elucidate any potential role of MKRN1 in fat deposition and obesity. No differences in body weight were observed under the standard chow feeding conditions. On the other hand, *MKRN1*-null mice fed an HFD exhibited 25–30% lower body weights than those of WT mice, without any obvious differences in food intake (Fig. 3a–c and Supplementary Fig. 4a, b). We observed similar results for female *MKRN1*-null mice compared with WT mice under HFD conditions (Supplementary Fig. 5a–d). Little difference was observed between *MKRN1*-null and WT mice in plasma leptin levels, supporting the finding that there was no difference in the amount of feeding (Supplementary Fig. 4c). In addition, there were no significant changes in gross faecal energy, the levels of TG and FFA in faeces or bone length (Supplementary Fig. 4d, e), suggesting that the decreased body weights of the *MKRN1*-null mice were not due to changes in nutritional excretion or morphology. Notably, micro-computed tomography (micro-CT) revealed a reduction in the total body fat content, including the volume of subcutaneous, visceral and abdominal fat, and an increase in the lean mass/body weight ratio (Fig. 3d, e). Accordingly, decreased sizes of individual adipocytes in epididymal and subcutaneous fat pads were observed in *MKRN1*-null mice (Fig. 3f, g). Consequently, HFD-induced hyperlipidaemia was almost completely normalised in *MKRN1*-null mice, which displayed similar plasma lipid concentrations to those of WT mice fed a standard chow diet (Fig. 3h). We further tested whether a lack of MKRN1 induces AMPK activation in vivo, contributing to the metabolic phenotype of *MKRN1*-null mice. MKRN1 is widely expressed throughout the body^40,41^. Intriguingly, *MKRN1*-null mice exhibited chronic AMPK activation in the liver and white (WAT) and brown (BAT) adipose tissues (Fig. 4g, Supplementary Fig. 6a–c, Supplementary Fig. 10b, and Supplementary Fig. 11c), regardless of nutritional status. However, no changes in AMPK activity were observed among other metabolic organs, including the hypothalamus, skeletal muscle and pancreas (Supplementary Fig. 6d–f). In particular, the lack of an apparent change in hypothalamic AMPK activity and its downstream target, pACC, strongly supported the near-equal food intake observed in WT and *MKRN1*-null mice, as previously reported^23^ (Supplementary Fig. 6d). Notably, there was no obvious difference in the liver mRNA levels of MKRN1 between mice fed normal chow and mice fed an HFD, indicating the possible existence of pathway regulating the interaction between MKRN1 and AMPK (Supplementary Fig. 6g). Together, these data support the hypothesis that MKRN1 deficiency potentially protects against nutrient overload-induced obesity through the tissue-specific regulation of AMPK.

### Hepatic AMPK activation prevents steatosis in MKRN1-knockout mice

The reduction in FFA-induced hepatic steatosis in MKRN1-ablated HepG2 cells and the chronic AMPK activation in the livers of *MKRN1*-null mice led us to investigate the effect of MKRN1 deficiency on HFD-induced NAFLD. The lack of MKRN1 dramatically changed the morphology (Fig. 4a), histological appearance (Fig. 4b) and weight (Fig. 4c) of the fatty liver associated with an HFD. Notably, size and morphology of the livers from *MKRN1*-null mice fed an HFD were similar to those of mice maintained on a chow diet. A substantial reduction in the number and volume of enlarged lipid droplets was observed in *MKRN1*-null mice (Fig. 4b, d). Likewise, a decrease in the hepatic TG content was observed in *MKRN1*-null livers compared with that in the WT livers (Fig. 4e). No significant difference in the F4/80-positive macrophage populations was observed, indicating that immune responses did not occur (Fig. 4f). Despite the suppression of AMPK activity under conditions of nutrient overload^5,10^, the lack of MKRN1 caused the remarkable stabilisation of AMPK, which was accompanied by increases in AMPK and ACC phosphorylation (Fig. 4g). Accordingly, the expression levels of transcription factors such as *SREBP-1* and *ChREBP* were suppressed in *MKRN1*-null livers (Fig. 4h). Furthermore, in response to the HFD, plasma levels of aspartate aminotransferase (AST) and alanine aminotransferase (ALT), which are biomarkers employed to diagnose hepatic damage, were elevated in WT mice but were significantly reduced in *MKRN1*-null mice (Fig. 4i). There was no obvious difference in glycogen accumulation or the generation of lactate in the liver between *MKRN1*-null and WT mice, suggesting that metabolic pathways related to lipogenesis are most strongly affected by MKRN1 depletion in the liver (Supplementary Fig. 7). Based on these data, MKRN1 deficiency promotes hepatic AMPK activation, possibly resulting in reduced hepatic lipid accumulation and the prevention of NAFLD.

Next, AMPK activity was inhibited through adenoviral delivery of small hairpin RNAs (shRNAs) targeting AMPKα2 to determine the role of hepatic AMPK activation in the prevention of hepatic steatosis in *MKRN1*-null mice (Fig. 4j and Supplementary Fig. 8). AMPK activation in the *MKRN1*-null liver was completely reversed by AMPKα2 knockdown (Supplementary Fig. 8b). Notably, the substantial reductions in lipid droplets and TG contents observed in the *MKRN1*-null liver were dramatically restored by hepatic AMPKα2 knockdown (Fig. 4k, l), without affecting the body weights of *MKRN1*-null mice. Finally, the levels of the lipogenesis markers *SREBP-1* and *ChREBP*, which were decreased in *MKRN1-*null mice, were restored upon AMPKα2 knockdown, corroborating the physiological data (Fig. 4m). Thus, MKRN1 deficiency protects against diet-induced hepatic steatosis in an AMPK-dependent manner.

### MKRN1 ablation alters metabolism in the liver and adipocytes

To investigate how cellular processes are influenced by MKRN1 ablation at the global level, we performed mRNA-sequencing analysis of the liver and adipose tissues from *MKRN1*-null and WT mice. Using mRNA-sequencing data, we identified 2310 (492 upregulated and 1879 downregulated) and 3383 (1379 upregulated and 2121 downregulated) differentially expressed genes (DEGs) between *MKRN1*-null and WT mice in the liver and adipose tissues, respectively (Supplementary Data 1).

**Fig. 5:**
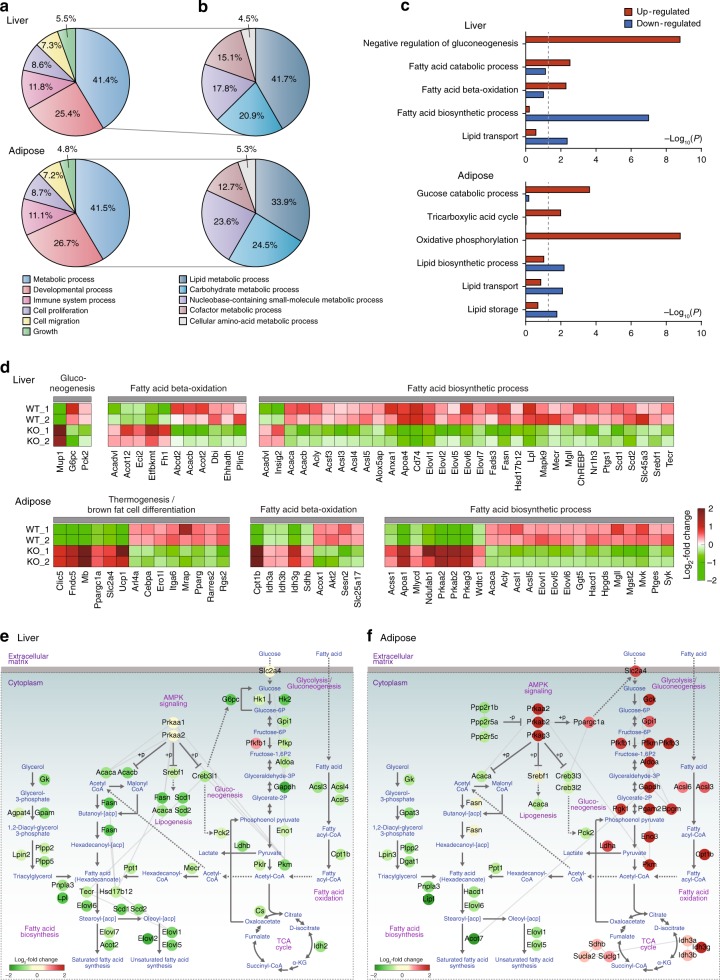
Regulation of lipid and glucose metabolism by MKRN1 and AMPK. **a**, **b** Relative proportions of DEGs in *MKRN1*-null liver and adipose tissues according to their associated GOBPs. The GOBP terms at level 1 (**a**) and levels 2–4 (**b**) were used for general cellular processes and metabolic processes, respectively. **c** Cellular processes related to lipid and carbohydrate metabolism enriched by the up- and downregulated genes identified in *MKRN1*-null liver or adipose tissue. The bars represent −log_10_ (*P*-value), where the *P*-value is the significance of the processes being enriched by the up- or downregulated genes. **d** Heat maps showing the changes in the expression of DEGs involved in fatty acid biosynthesis and β-oxidation, gluconeogenesis and thermogenesis. The colour bar shows the gradient of the log_2_ fold changes of mRNA expression levels in MKRN1-depleted samples relative to those in WT samples. **e**, **f** Network models describing alterations of metabolic reactions regulated by DEGs in *MKRN1*-null livers (**e**) and adipose tissue (**f**). Arrows denote metabolic reactions, and dotted lines denote the transportation of molecules or regulation involving intermediate regulators between the linked molecules. Node colours represent up- (red) or downregulation (green) in *MKRN1*-null livers or adipose tissue. The colour bar represents the gradient of the log_2_ fold changes of mRNA expression levels induced by MKRN1 ablation relative to those in WT

To examine how metabolic processes are affected by MKRN1 ablation, further functional enrichment analyses of the DEGs in the liver and adipose tissue were carried out. Among different functional categories, the DEGs were found to be most strongly associated (41.4% in the liver and 41.5% in the adipose tissue) with metabolism, suggesting that MKRN1 depletion significantly impacts metabolic processes in the liver and the adipose tissue (Fig. 5a). Notably, lipid metabolism (41.7% in the liver and 33.9% in the adipose tissue) was one of the processes that was most influenced by MKRN1 ablation, together with carbohydrate metabolism (20.9% in the liver and 24.5% in the adipose tissue) (Fig. 5b). Among cellular processes related to lipid metabolism, lipid anabolism pathways (i.e., lipid and fatty acid biosynthetic processes) were significantly (*P* < 0.05) downregulated in both *MKRN1*-null liver and adipose tissues (Fig. 5c–f). In the liver, the genes involved in the biosynthesis of saturated and unsaturated fatty acids from acetyl-CoA (*Acaca/b*, *Fasn*, *Tecr*, *Hsd17b12*, *Elovl1/2/5/6/7*, *Scd1/2* and *Acot2*) were downregulated, as were the genes involved in the conversion of glycerol to fatty acids (*Gk*, *Agpat4*, *Gpam*, *Lpin2*, *Plpp2/5*, *Pnpla3* and *Lpl*), suggesting systematic suppression of fatty acid biosynthetic pathways by MKRN1 ablation (Fig. 5e). In adipose tissue, similar downregulation of fatty acid biosynthetic pathways was observed (Fig. 5f). On the other hand, the genes involved in lipolysis (*Acsl3/4/5* and *Cpt1b*) were downregulated in the liver, but those (*Acsl3/6* and *Cpt1b*) in the same pathway were upregulated in the adipose tissue (Fig. 5e, f). These data suggest that MKRN1 ablation results in stricter regulation of fatty acids in the adipose tissue by decreasing lipogenesis and increasing lipolysis with respect to the regulation of these processes in the liver, by which both lipolysis and lipogenesis are decreased.

In addition to lipid metabolism, carbohydrate metabolism was significantly influenced by MKRN1 ablation (Fig. 5b). In the liver, the genes involved in glycolysis (*Hk1/2*, *Gpi1*, *Pfkp*, *Aldoa*, *Gapdh*, *Eno1*, *Pkm* and *Pklm*) and gluconeogenesis (*G6pc* and *Pck2*) were downregulated (Fig. 5e). In the adipose tissue, however, the genes involved in glucose uptake (*Slc2a4/Glu4*) and glycolysis were upregulated (Fig. 5f), as were those involved in lipolysis. On the other hand, lactate dehydrogenase was upregulated in the adipose tissue (*Ldha*), but downregulated (*Ldhb*) in the liver (Fig. 5e, f). Glucose metabolism is linked to fatty acid metabolism through acetyl-CoA. Up- and downregulation of glucose utilisation in the adipose tissue and the liver, respectively, reflect an increased amount of acetyl-CoA and, thus, a larger supply of acetyl-CoA for fatty acid biosynthesis in the adipose tissue than in the liver, which might lead to the upregulation of lipolysis in the adipose tissue (Fig. 5f). Furthermore, the genes involved in BAT thermogenesis (*Ucp1*, *Clic5* and *Ppargc1a*) were significantly upregulated in *MKRN1*-null adipose tissue (Fig. 5d). mTOR signalling was previously shown to be one of the main targets of *AMPK*^42,43^. Analyses of the mTOR pathway showed that mTOR and its upstream (*Hras*, *Mapk3*, *Pik3r1* and *Rheb*) and downstream (*Eif4e*, *Sgk1*, *Pparg* and *Srebf1*) genes were downregulated in the liver, while its upstream (*Pik3r1/3*, *Pik3cb* and *Akt2*) and downstream (*Rps6kb2*, *Eif4e*, *Prkca/g* and *Pparg*) genes were downregulated in the adipose tissue (Supplementary Fig. 9). These findings suggest that the activation of AMPK by MKRN1 ablation negatively regulates mTOR signalling in the liver and the adipose tissue. Taken together, these data suggest systematic customised regulation of fatty acid and glucose metabolism in the liver and the adipose tissue by MKRN1 and AMPK.

### MKRN1 deficiency suppresses HFD-induced diabetes

MKRN1 depletion promoted AMPK activation in adipose tissue, in addition to hepatocytes (Fig. 4g and Supplementary Fig. 6a–c). A lack of AMPK in adipocytes exacerbated the detrimental effects of an HFD, including NAFLD and insulin resistance, due to reductions in the metabolic activity of BAT^19^. In addition, AMPK activity in BAT has been implicated in the regulation of uncoupling protein 1 (UCP1) induction during thermogenesis^44^. Therefore, we postulated that AMPK activation in adipocytes would protect against obesity-induced BAT dysfunction^45–47^ and contribute to the anti-obesity effects on *MKRN1*-null mice. Consistent with the body weight results, the morphology of BAT (Supplementary Fig. 10a), UCP1 expression (Supplementary Fig. 10b), EE and oxygen consumption (VO_2_) (Supplementary Fig. 10c) did not differ between different genotypes fed a chow diet. In contrast, *MKRN1*-null mice fed an HFD showed reduced numbers of lipid droplets in BAT (Supplementary Fig. 11a, b), along with AMPK stabilisation and activation (Supplementary Fig. 11c) and expression of thermogenic genes in BAT, such as *UCP1* (Supplementary Fig. 11c, d). Under HFD feeding conditions, the respiratory exchange ratio of both WT and *MKRN1*-null mice varied in the range of 0.7–0.8, indicating complete dependence on fatty acid metabolism (Supplementary Fig. 11f). Contrary to our expectations, indirect calorimetry measurements performed for 2 days showed no significant difference in EE among genotypes when the mice were fed an HFD (Supplementary Fig. 11g). The overall data show that *MKRN1*-null mice maintain BAT activity as a result of resistance to weight gain when fed an HFD.

AMPK activation has been implicated in the regulation of BAT activity and hepatic gluconeogenesis, which are closely linked to insulin resistance^7,19^. We observed a decrease of rate-limiting enzymes, including glucose-6-phosphatase and phosphoenolpyruvate carboxykinase, in hepatic gluconeogenesis (Fig. 5d), and an increase in the thermogenic function of BAT and WAT in *MKRN1*-null mice (Fig. 5 and Supplementary Fig. 11). Consistent with these findings, *MKRN1*-null mice placed on an HFD displayed lower fasting plasma insulin levels (Supplementary Fig. 12a), a decrease in the HFD-induced enlargement of pancreatic islets (Supplementary Fig. 12b), increased glucose clearance, as indicated by glucose tolerance testing in both male and female mice (Supplementary Fig. 12c), and greater insulin sensitivity, as indicated by insulin tolerance testing (Supplementary Fig. 12d). Moreover, MKRN1 deficiency promoted insulin-stimulated AKT phosphorylation, a signature signalling pathway that reflects insulin sensitivity in the liver (Supplementary Fig. 12e). However, these pathways were not specifically activated in the skeletal muscle or WAT (Supplementary Fig. 12e). Collectively, AMPK activation alleviates obesity-induced insulin resistance and T2D in *MKRN1*-null mice.

### An acute reduction of hepatic MKRN1 level improves NAFLD

**Fig. 6:**
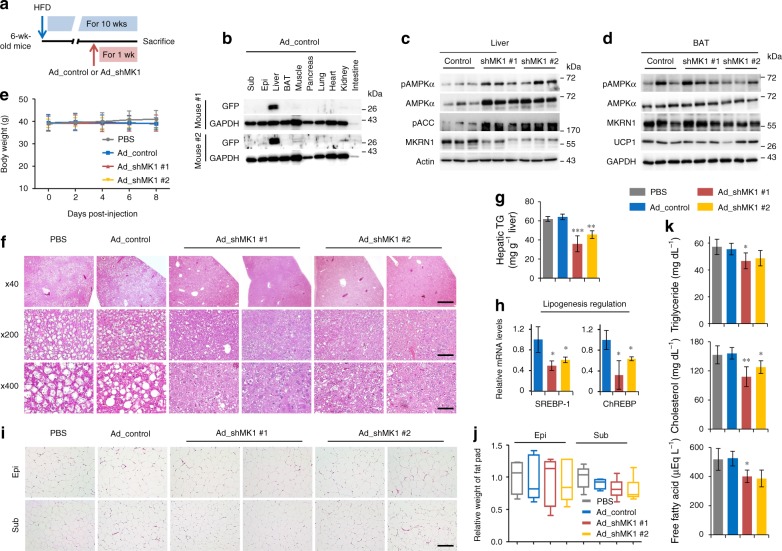
Ablation of hepatic MKRN1 improves hepatic steatosis in diet-induced obese mice. **a** Male B6 mice (6 weeks old) were fed an HFD for 9 weeks and then injected with either PBS or adenoviruses expressing GFP via the tail vein (Ad_control, Ad_shMK1 #1 and #2 (shRNA targeting MKRN1)). After 1 week of continuous HFD feeding, the mice were sacrificed and analysed. **b** Immunoblot analysis of GFP expression in extracts from the indicated tissues (Sub subcutaneous fat, Epi epididymal fat) of mice infected with adenovirus (*n* = 2). **c**, **d** Liver (**e**) and BAT (**d**) lysates from adenovirus-injected mice were analysed by immunoblotting as indicated. **e** Body weights of male mice fed with an HFD were measured in every 2 days for 8 days (*n* = 5 mice per group). **f** Representative H&E staining of liver sections. Scale bar = 50, 100 and 500 µm. **g** Liver TG levels were measured. **h** Lipogenic enzymes were analysed via quantitative real-time PCR (*n* = 5 mice per group). **i** Representative H&E staining of epididymal fat (top) and subcutaneous fat (bottom) sections. Scale bar = 500 µm. **j** The weights of fat tissues were recorded following sacrifice. **k** Liver TG levels (*n* = 5 mice per group). Plasma lipid (TG, cholesterol and FFA) concentrations in 24-h-fasted mice (*n* = 5 mice per group). The data are presented as the mean ± s.d. Two-tailed Student’s *t*-test; ^*^P ≤ 0.05, ^**^P ≤ 0.01, ***P ≤ 0.001.

The metabolic consequences of chronic AMPK activation exhibited by *MKRN1*-null mice led us to investigate whether hepatic AMPK activation was sufficient to improve NAFLD without affecting the activation of AMPK in adipose tissue and whether the acute reduction of MKRN1 expression would alleviate the symptoms of hepatic steatosis in obese mice. We generated adenoviruses expressing two independent shRNAs targeting MKRN1 (Ad-shMKRN1 #1 and #2) and injected these viruses into diet-induced obese mice via the tail vein to answer these questions (Fig. 6a). Following the delivery of adenovirus to mice, which was confirmed by green fluorescent protein (GFP) co-expression, the adenovirus was predominantly observed in the liver (Fig. 6b). The administration of both Ad-shMKRN1 #1 and #2 successfully ablated MKRN1 expression and led to the activation of hepatic AMPK and ACC phosphorylation (Fig. 6c). No apparent changes in BAT were observed (Fig. 6d). Notably, while there was no effect on the body weight of the mice, hepatic MKRN1 knockdown in obese mice dramatically reversed the generation of enlarged lipid droplets and reduced TG levels in the liver (Fig. 6e–g). In addition, the alleviation of hepatic steatosis accompanied the salutary effects on hyperlipidaemia in Ad-shMKRN1-injected obese mice (Fig. 6k). The decreases in SREBP-1 and ChREBP support the reduction of lipid droplets and hypolipidaemia observed in the liver upon MKRN1 knockdown (Fig. 6h). On the other hand, the knockdown of MKRN1 in the liver had no apparent effect on the adipose tissues (Fig. 6i, j). These observations reveal that MKRN1 could be a potential therapeutic target for alleviating the symptoms of hepatic steatosis.

## Discussion

The pharmacological and genetic activation of AMPK is associated with a wide range of effects on whole-body metabolism, including the suppression of gluconeogenesis and lipogenesis in the liver, promotion of glucose consumption in the skeletal muscle and potential functions in FAO and lipolysis in the liver and BAT^5,26,48^. Thus, AMPK has been suggested as an attractive target for the treatment of metabolic disorders. However, extended follow-up studies have presented issues and challenges because of the complexities of chronic activation of AMPK and AMPK-independent actions. Here the inhibition of the E3 ubiquitin ligase MKRN1 was sufficient to chronically activate AMPK in the liver and adipose tissues and subsequently led to systemic effects by preventing hepatic lipid accumulation and insulin resistance and promoting anti-obesity effects on diet-induced obese mice. Our findings highlight a therapeutically exploitable axis regulating AMPK, without the ramifications of activation in central circuits.

Substantial evidence supporting the beneficial effects of AMPK activation on peripheral tissues has rapidly emerged. Chronic treatment with direct AMPK activators, such as A769662^17^, PF-739^49^ or AICA [5-aminoimidazole-4-carboxamide] riboside^18^, restrains hepatic lipid accumulation and promotes glucose disposal. In a more recent study, genetic activation of hepatic AMPK through the γ1 mutation (γ1^D316A^) compellingly prevented hepatic TG accumulation in mice placed on a high-fructose diet^16^. AMPK has also been implicated in BAT functions and energy metabolism^44,50^, suggesting its possible therapeutic use for obesity. Recently, Mottillo et al.^19^ reported a role for adipocyte AMPK in BAT thermogenesis, with implications for hepatic steatosis and insulin resistance. However, in this study, diet-induced obesity was not significantly affected by the loss of AMPK. Because mice placed on a long-term HFD show a decrease in adipose AMPK activity^5,10^, the additional effect of AMPK deficiency on obesity might not be reflected in these models. Supporting this hypothesis, greater weight gain after a relatively short-term HFD challenge has been observed in the adipose tissue of *AMPK*-null mice^19^. The *MKRN1*-null mouse displays liver- and adipose tissue-specific AMPK activation. Thus, hepatic AMPK activation induced by MKRN1 depletion protects the liver from lipid accumulation, while simultaneously suppressing the expression of gluconeogenic genes.

Functional enrichment and network analyses revealed the differential regulation of AMPK, mTOR and fatty acid and glucose metabolism between the liver and the adipose tissue. Although we showed that AMPKs were regulated at the protein level by MKRN1, the mRNA expression levels of *Prkaa2*/AMPKα2, *Prkab2*/AMPKβ2 and *Prkag3*/AMPKγ3 were increased in adipose tissue by *MKRN1* ablation but showed no change in the liver, suggesting that there are additional metabolic needs to be accommodated by the increases of AMPKs, even at the mRNA level in adipose tissue (Supplementary Fig. 9a, b). In mTOR signalling, the MAPK pathway (*Hras*, *Mapk3*, *Pik3r1* and *Rheb*) and its upstream and downstream factors (*Eif4e*, *Sgk1*, *Pparg* and *Srebf1*) were predominantly downregulated in the liver, while the PI3K-AKT pathway (*Pik3r1/3*, *Pik3cb* and *Akt2*) and its downstream factors (*Rps6kb2*, *Eif4e*, *Prkca/g* and *Pparg*) were predominantly downregulated in the adipose tissue (Supplementary Fig. 9a, b). Although Pparα is one of the major transcription factors involved in lipid metabolism, there was no apparent change in the expression of Pparα in the liver or adipose tissue. We cannot, however, exclude the possibility that MKRN1 regulates Pparα at the post-translational level, since Pparγ is known to be a target of MKRN1^36^. Further studies are required to address this issue in the future. Although genes related to lipogenesis was consistently downregulated in both the liver and the adipose tissue, the catabolism of fatty acids and glucose was upregulated in the adipose tissue but downregulated in the liver, indicating the differential regulation of metabolic pathways in *MKRN1-*null mice (Fig. 5e, f). Importantly, further experiments including direct measurements of the lipid components in the liver should be carried out to reach explicit conclusions about the reduction of actual biochemical outcomes derived from comprehensive changes of the lipogenic genes. Of note most of the phenotypes observed in the *MKRN1*-null mouse could also be driven by AMPK-mediated phosphorylation of ACC, which suppresses its function. Thus, we cannot exclude the possibility that this process might have considerable negative effects on the accumulation of lipids in *MKRN1-*null mice, in addition to the downregulation of lipogenic gene expression. Taken together, the differential regulation of the above processes by MKRN1 might be ascribed to additional metabolic needs arising from the increased uptake and utilisation of glucose in adipose tissue.

Collectively, our findings illuminate a previously undescribed post-translation mechanism in which AMPK activity is regulated by E3 ubiquitin ligase and provides an attractive therapeutic strategy for the treatment of obesity and its related comorbidities. Because MKRN1 is able to degrade both of α subunits, a competitive antagonist capable of interfering with the interactions between AMPKα and MKRN1 or an enzyme inhibitor targeting ubiquitin ligase activity may lead to potent AMPK activation in an organ-specific manner. This strategy may represent an effective means of ameliorating diet-induced obesity and insulin resistance by exploiting not only hepatic AMPK activation and its subsequent contributions to de novo lipogenesis but also adipose AMPK activation, leading to an increase in the energy-burning capacity of BAT.

## Methods

### Mice

*MKRN1*-null (*MKRN1*^−*/*−^) mice were obtained from T. A. Gray (David Axelrod Institute, USA)^39^ and were further backcrossed with C57BL/6 mice until N10. *MKRN1*-null mice and their WT littermates (*MKRN1*^*+/+*^) were bred from *MKRN1*^*+/*−^ mice and were randomly grouped for all the experiments. The primers employed for MKRN1 genotyping were 5′-TGA CAGGCCACAGTGAACTC-3′, 5′- GGCAAAGCTGCTTCTTTGTCTCC-3′ and 5′-CAA AGGGAAAGGGTAAAGTGGTAGGG-3′, which amplified an approximately 1000-bp DNA fragment in *MKRN1*^−/−^ mice and an 820-bp DNA fragment in *MKRN1*^*+/+*^ mice. For HFD-induced obesity experiments, male and female *MKRN1*^*+/+*^ and *MKRN1*^−*/*−^ mice were placed on either an HFD (60% calories from fat; D12492, Research DIET Inc., NJ, USA) or a standard chow diet (17% calories from fat; 38057, Cargill Agri Purina Inc., Republic of Korea) for 16 weeks starting at 6–8 weeks of age. For adenovirus experiments, male C57BL/6 mice (6 weeks old) were placed in individual cages, treated with an HFD for 9 weeks and injected with adenovirus via the tail vein. After 1 week of continuous HFD feeding, the mice were sacrificed. All the mice were maintained under temperature-controlled and light-controlled (standard 12-h light–dark cycles) conditions and provided with food and water ad libitum. All the procedures were reviewed and approved by the Institutional Animal Care and Use Committees (IACUC) of the Laboratory Animal Research Center at Yonsei University (IACUC-A-201506-311-01).

### Cell lines and transfection

*MKRN1*^*+/*−^ mice with FBV/NJ × C57BL/6J background were backcrossed with the B6 background mice until N6. *MKRN1*^*+/*−^ mice were mated to produce MEFs with or without MKRN1. To acquire MEFs of 13.5 embryo day, embryos washed with phosphate-buffered saline (PBS; Welgene) were minced followed by incubation with the treatment of 3 ml trypsin/EDTA (Gibco) at 37 °C for 15 min. The MEFs were grown in 20 ml Dulbecco’s modified Eagle medium (DMEM) containing 10% foetal bovine serum (FBS; GIBCO) for 4–8 h followed by the change of media with DMEM containing 10% FBS. The confluent MEFs were then subcultured at 1:3 ratio^33^. HEK293T (a human embryonic kidney cell line) and HepG2 (a human hepatocellular carcinoma cell line) cells were acquired from the American Type Culture Collection (ATCC, Manassas, VA). The phenotypes of these cell lines were authenticated by ATCC on a regular basis. All the cell lines employed here were negative for mycoplasma when detected using an e-Myco plus Mycoplasma PCR Detection Kit (Intron, Gyeonggi-do, Republic of Korea) and were protected from mycoplasma infection by treatment with Plasmocin^TM^ (InvivoGen, CA, USA). Lipofectamine 2000 (Invitrogen, Carlsbad, CA, USA), PEI (Sigma-Aldrich, St. Louis, MO, USA) or Lipofectamine RNAiMAX (Invitrogen) were employed to transfect plasmid DNA or siRNA.

### Reagents

Phospho-AMPKα (1:1000, Thr 172) (2535), AMPKα (1:1000, 2603), AMPKα1 (1:1000, 2795), AMPKα2 (1:1000, 2757), AMPKβ1 (1:1000, 4178), AMPKγ1 (1:1000, 4187), phospho-ACC (1:1000, 3661), ACC (1:1000, 3676), phospho-AKT (1:1000, Ser 473) (9271) and AKT1 (1:2000, 2967) antibodies were acquired from Cell Signaling Technology (Danvers, MA, USA). The UCP1 antibody (1:1000, ab10983) and mouse AMPKα1 (1:1000, ab3759) were procured from Abcam (Cambridge, MA, USA). Mouse AMPKα2 (1:1000, AF2850) was purchased from R&D systems Inc. (MN, USA). FLAG (1:3000, F3165 and F7425) and β-actin (1:10000, A5316) antibodies were procured from Sigma-Aldrich. The MKRN1 antibody (1:3000, A300-990A) was procured from Bethyl Laboratories. The mono- and polyubiquitin chain antibodies (1:1000, FK2, Biomol, PW0150) were purchased from Enzo Life Sciences. The HA antibody (1:3000, 12013819001) was obtained from Roche. GAPDH (1:5000, sc-25778), GFP (1:5000, sc-8334) and HA (hemagglutinin) (1:3000, sc-7392 and sc-805) antibodies were procured from Santa Cruz Biotechnology (Dallas, TX, USA). MG132 (M-1157) was purchased from A.G. Scientific (CA, USA). CHX (C4859) was purchased from Sigma-Aldrich.

### Indirect calorimetry

Metabolic performance (energy intake and EE) was studied using a PHENOMASTER automated combined indirect calorimetry system (TSE System GmBH, Bad Homburg, Germany). For the experiment, the mice first acclimated for 48 h in a metabolic chamber provided with food and water were subsequently evaluated for 3 days for oxygen consumption (VO_2_), carbon dioxide production (VCO_2_) and food consumption. The temperature for these studies was kept at 22 °C, with a 12-h-light/dark cycle. Standard in-house software was used for EE.

### Micro-positron emission tomography/-CT image acquisition and analysis

After 6 h of fasting, each mouse was intravenously injected with 200 µCi of F-18 fluorodeoxyglucose via the tail vein and then maintained in a box at 4 °C for 1 h. Each mouse was anaesthetised during the scan with isoflurane (2.5% flow rate). The animals were placed in a prone position on a standard mouse bed. The limbs of the mice were positioned laterally from the body to obtain uniform CT images. Whole-body CT images were obtained employing a micro-positron emission tomography (PET)/-CT scanner (nanoScan PET/CT, Mediso Inc., Budapest, Hungary). The X-ray source was fixed at 200 µA and 45 kVp with 0.5 mm for CT image acquisition. The CT images were recreated through cone beam reconstruction using a Shepp filter with a cutoff at the Nyquist frequency and a binning factor of 4, which resulted in an image matrix of 480 × 480 × 632 with a voxel size of 125 µm. The PET images were recreated using the Tear-Tomo Real 3D PET engine (nanoScan PET/CT, Mediso Inc.). The weight of each mouse was determined before taking image of lean body mass (LBM). The overall body image, including total and visceral adipose tissue volume was measured as reported previously^51^. The LBM is defined as the weight of the body without the weight of fat. Here LBM was analysed according to the following equation, where the density of fat was 0.9 g cm^−3^. Detailed methods are described in a previous report^51^.

1 $$\ {\mathrm{LBM}} = \{ {\mathrm{body}}\;{\mathrm{weight}}\,\left( {\mathrm{g}} \right)-({\mathrm{total}}\;{\mathrm{adipose}}\;{\mathrm{tissue}}\;{\mathrm{volume}}\,({\mathrm{cm}}^3) \times 0.9\,({\mathrm{g}}\;{\mathrm{cm}}^{ - 3}))\}$$

### Glucose tolerance test (GTT), insulin tolerance test (ITT) and insulin stimulation

The mice were treated with an HFD for 16 weeks and tested after an overnight (16-h) fast. The glucose tolerance test (GTT) was performed via oral glucose (G8769, Sigma-Aldrich) administration at a dose of 1 g kg^−1^ after an overnight fast. The blood glucose levels were analysed at 0, 15, 30, 60 and 120 min post-injection using GlucoDr. Plus (AGM-3000, Allmedicus). For insulin tolerance test assays insulin (I9278, Sigma-Aldrich) stimulation was accomplished via intraperitoneal insulin injection at a dose of 0.75 U kg^−1^ (final volume 125 μl) after an overnight fast. Glucose levels were measured at 0, 15, 30, 60, 90 and 150 min after injection. Protein samples of skeletal muscle, liver and epididymal fat pads were collected 10 min after injection for western blot analysis.

### Faecal energy content

Faeces were collected and dried in a drying oven until the samples were completely free of water. The energy contents of 0.5–1 g of dried samples were measured using a bomb calorimeter (Parr6400 Calorimeter, IL, USA). TG and fatty acid contents were measured using enzymatic methods (AU680, Beckman Coulter, USA).

### Metabolomic analysis

Metabolomic analysis was performed employing the Basic Scan package from Human Metabolome Technologies (HMT) Inc. (Tsuruoka, Japan) and capillary electrophoresis–time-of-flight mass spectrometry (CE-TOFMS) as described previously^52^. Metabolites were extracted from *MKRN1*^−*/*−^ MEFs and the MEFs of WT littermates as the manufacturer suggested (E-130637; HMT). Briefly, the cells were washed twice using a 5% (w/w) mannitol solution, added to methanol to extract metabolites and treated with internal standards solution 1 (HMT). The extracted solution was centrifuged for 5 min at 2300 × *g* and 4 °C. The proteins of the upper aqueous layer were removed using a Millipore 5-kDa cutoff filter. The filtrate was subsequently lyophilised and dissolved in 50 µl of Milli-Q water and analysed via CE-TOFMS. Automatic integration software (MasterHands ver. 2.16.0.15 developed at Keio University) was employed to process peaks from CE-TOFMS analysis and to acquire information of *m*/*z*, migration time (MT) and a relative peak area. The HMT metabolite database based on the peak MTs and *m*/*z* values established using TOFMS were employed to assign all the target metabolite.

### Glucose consumption measurements

HepG2 cells and MEFs were cultured in fresh high-glucose (4500 mg l^−1^) medium for 24 or 48 h at 24 h after siRNA transfection. Live cells were treated with 2-[*N*-(7-nitrobenz-2-oxa-1,3-diazol-4-yl) amino]-2-deoxy-d-glucose for 30 min (20 μM), and glucose uptake was measured by quantifying fluorescence using flow cytometry.

### FFA-induced steatosis

For the FFA stock solution, oleic acid (OA, 100 mM) was conjugated with 1% (w/v) bovine serum albumin (BSA). The required volume of the FFA stock was added to the medium to obtain a 0.5 or 1 mM concentration of fatty acids for experiments. HepG2 cells were treated with OA complexed with BSA or fatty acid-free BSA (as the control) for 24 h and then stained with ORO (Oil Red O staining) . Images were procured using aNikon eclipse 80i light microscope (Nikon, Tokyo, Japan) and analysed with Nikon NIS-Elements F 3.2 software. Cellular TGs were also extracted from FFA-treated HepG2 cells and measured.

### TG measurement

For TG measurement, 0.2-g liver samples from male *MKRN1*^*+/+*^ and *MKRN1*^−*/*−^ mice or FFA-treated HepG2 cells were homogenised in 5% NP-40 in water, and the samples were then heated to 95 °C and cooled to room temperature twice. TG contents were analysed using the Triglyceride Quantification Colorimetric Kit (Bio Vision, CA, USA). TG levels were calculated from measurements of the absorbance at 570 nM.

### FAO measurement

For FAO measurement in HepG2 cells, the cells were plated in triplicate in 96-well plates at a density 6 × 10^4^ cells per well. The culture medium was changed to glucose deprivation medium (1 mM of glucose, 1 mM of l-glutamine, 0.5 mM of l-carnitine, 1% FBS, penicillin/streptomycin solution, pH 7.4) overnight, followed by replacement of the medium with FA/FA-free measurement medium (ab217602) with or without carbonyl cyanide *p*-trifluoro-methoxyphenylhydrazone (FCCP) in the presence of an O_2_ consumption reagent (ab197243). Measurements were performed using a fluorescent plate reader (GloMAX-Discover, Promega). For FAO measurement in MEFs, an extracellular flux (XF24) analyser (Seahorse Bioscience) was used to measure oxygen consumption rates (OCR). The MEFs were plated at a density of 6 × 10^4^ cells per well in triplicate in custom-designed 24-well plates (Cat. No. 103010-100). OCR was evaluated over time with or without the addition of oligomycin (2.5 μM), followed by the addition of FCCP (1.6 µM), and rotenone/antimycin-A (0.5 μM) with or without palmitate-conjugated BSA (Cat. No. 102720-100).

### Lactate measurement

The l-Lactate Assay Kit II (1200051002, Eton Bioscience) was employed to quantitate the amount of lactate.

### Serum biochemistry

Fasting plasma insulin levels were measured in mice fasted for 16 h using the Mouse Metabolic Hormone Magnetic Bead Panel (Metabolism Multiplex Assay, MMHMAG-44K, Millipore). Plasma TG, cholesterol and FFA concentrations were determined using enzymatic methods (Roche Diagnostics, Mannheim, Germany) with a Hitachi 7600 clinical chemistry analyser (Hitachi Ltd., Tokyo, Japan) in overnight-fasted mice. Serum AST and ALT levels were measured via ultraviolet methods (Roche Diagnostics) with a Hitachi 7600 analyser, according to the recommended protocols from the International Federation of Clinical Chemistry.

### Histology and immunohistochemistry

Tissues fixed in 10% buffered formalin were embedded in paraffin followed by staining with haematoxylin and eosin or periodic acid Schiff for glycogen detection. Images were obtained using a Leica DM2500 microscope (Leica Microsystems, IL, USA) and analysed with Leica LAS V4.7 software. For immunohistochemistry, the ImmPRESS Peroxidase Polymer kit (Vector Laboratories, Burlingame, CA, USA) was employed. Briefly, the slides blocked with 2.5% horse serum were incubated overnight at 4 °C with the F4/80 antibody (1:200; sc-377009; Santa Cruz Biotechnology) followed by washing. Slides were then incubated for 30 min with an appropriate peroxidase polymer-linked secondary antibody. For colorimetric detection the slides were stained with ImmPact DAB substrate (SK-4105, Vector Laboratories) followed by counterstaining with Meyer’s haematoxylin for 10 s. For Oil Red O staining, formalin-fixed tissues were equilibrated in 30% sucrose, embedded in OCT compound and snap-frozen in liquid nitrogen. Frozen sections were stained with Oil Red O (Sigma-Aldrich). Images were obtained using a Nikon eclipse 80i microscope and analysed with Nikon NIS-Elements F 3.2 software. The freehand area selection tool in ImageJ were employed to determine adipocyte size (mean area of white adipose cells, minimum *n* = 3 samples per group and *n* = 3 fields per section).

### Ubiquitination assay

The ubiquitination assay was conducted under denaturing conditions to detect ubiquitinated endogenous and overexpressed AMPKα proteins. Briefly, to detect proteins ubiquitinated with HA-conjugated ubiquitin or endogenously ubiquitinated proteins under denaturing conditions, cells were lysed by boiling for 10 min in PBS containing 1% SDS and 5 mM NEM (*N*-ethylmaleimide). The lysates were immunoprecipitated in lysis buffer (a final concentration of 0.1% SDS). For immunoblotting, proteins were transferred to polyvinylidene difluoride membranes and denatured using 6 M guanidine-HCl containing 20 mM Tris-HCl (pH 7.5), 5 mM mercaptoethanol and 1 mM phenylmethyl sulphonyl fluoride for 30 min at 4 °C. Ubiquitinated proteins were identified by horseradish peroxidase-conjugated anti-Ub antibodies (FK2, PW0150, Biomol)^35^. For in vitro ubiquitination assays, a 0.5-µg sample of bacterially purified recombinant proteins was incubated with 100 ng of E1 (UBE1, E-305, Boston Biochem, Cambridge, MA, USA), 250 ng of E2 (UbcH5c, E2-627, Boston Biochem) and 5 µg of ubiquitin (U-100H, Boston Biochem) in 20 µl of reaction buffer (40 mM of Tris, 50 mM of NaCl, 5 mM of MgCl_2_, 2 mM of ATP and 1 mM of dithiothreitol, pH 7.6) as indicated. The reaction was stopped after 3 h at 37 °C by the addition of SDS sample buffer and boiling.

### Protein purification and immunoprecipitation

Glutathione *S*-transferase (GST)-tagged recombinant MKRN1 and the AMPKα1, α2, β1 and γ1 proteins were purified from bacteria using GST Sepharose beads according to the manufacturer’s protocol (GE Healthcare).

Immunoprecipitation assay: The cells were lysed in lysis buffer (50 mM of Tris-HCl (pH 7.5), 150 mM of NaCl, 0.5% Triton X-100 and 1 mM of EDTA) containing a protease inhibitor cocktail. The cell lysates were then incubated with 1 µg of antibody with rotation, followed by incubation with 25 µl of protein G agarose (Invitrogen), and the precipitated proteins were eluted in SDS sample buffer under boiling conditions^53^.

### Recombinant adenoviruses

The adenoviral clone pAV-U6-GFP (control vector, Ad_control) and an adenoviral clone containing an shRNA for murine MKRN1 (Ad_shMKRN1) were purchased from ViGene Biosciences Inc. (Rockville, MD, USA). The adenoviruses were propagated in HEK293 cells according to the manufacturer’s protocol. Adenoviruses expressing a nonspecific RNAi control (Ad_US) and shRNA for murine AMPKα2 (Ad_shα2) were generated via homologous recombination between the adenovirus backbone vector pAD-Easy and the linearised transfer vector pADTrack. For animal experiments, the viruses were purified in a CsCl gradient, dialysed against PBS buffer containing 10% glycerol and stored at −80 °C. The mice were tail vein-injected with the recombinant adenovirus (0.5 × 10^9^ pfu per mice). Plasma ALT and AST levels were not significantly different between the mice in the same experimental groups that were injected with various adenoviruses. The shRNA sequences were as follows: Ad_shMKRN1 #1: 5′-CCGGGCGAGATGTTGCTTATGCTTTCTCGAGAAAGCATAAGCAACATCTCGC TTTTTG-3′; Ad_shMKRN1 #2: 5′-CCGGGAGTGGGACTTGTTTCACGATCTCGAGATC GTGAAACAAGTCCCACTCTTTTTG-3′; and Ad_shα2: 5′-CCATAAAGTGGCAGTTAAG ATCTTAAA-3′.

### Statistical analysis

All statistical tests were two sided, and the values are expressed as the means with 95% confidence intervals. The normality of the distribution was assessed with the Kolmogorov–Smirnov method. Statistically significant differences between any two groups were examined using unpaired two-tailed *t*-tests or Mann-Whitney *U* tests, depending on normality. Data involving more than two groups were assessed via analysis of variance using GraphPad Prism software (version 7; GraphPad Software Inc., La Jolla, CA). Analysis of covariance (ANCOVA) was employed to compare genotypes, with adjustment for the confounding effect of mouse body weight for EE. The EE ANCOVA method used in this work was provided by the NIDDK Mouse Metabolic Phenotyping Centers (MMPC, www.mmpc.org) on their Energy Expenditure Analysis page (http://www.mmpc.org/shared/regression.aspx) and supported by grants DK076169 and DK115255. A value of *P* < 0.05 was considered statistically significant.

### mRNA-sequencing and data analysis

Total RNAs were obtained from the liver and adipose tissues of *MKRN1*-null and WT mice. Poly(A) mRNA isolation from total RNA and fragmentation was performed using the Illumina TruSeq RNA Sample Prep Kit v2, according to the manufacturer’s instructions. Reverse transcription of RNA fragments was performed using Superscript II reverse transcriptase (Life Technologies). The adaptor-ligated libraries were sequenced on the Illumina HiSeq 4000 platform (Marcrogen, Korea). mRNA-sequencing analysis was performed with two independent replicates from each condition. Adapter sequences (TruSeq universal and indexed adapters) were removed from the resulting read sequences for each sample using cutadapter software^54^. Then, the remaining reads were aligned to the mouse GRCh38 genome using Tophat2 software (version 2.1.1) with the default parameters^55^. After alignment, we computed RPKM (reads per kilobase of target per million mapped reads) for the gene features (GTF file of GRCm38.91) using Cufflinks^56^.

### Identification of DEGs

The numbers of reads counted by HTseq were converted to log_2_ read counts after adding one to the read counts. The log_2_ read counts for the samples from each condition were then normalised via the trimmed mean of *M*-values normalisation in the edgeR package^57^. To identify DEGs, we performed integrative statistical hypothesis tests as previously described^58^. Briefly, for each gene, we calculated a *T*-statistic value using Student’s *t*-test as well as the log_2_ fold change in the comparison of *MKRN1*-null versus WT. We then estimated the empirical distributions of T-statistic values and the log_2_-fold-change for the null hypothesis (i.e., the genes are not differentially expressed) by performing all possible combinations of random permutations of the four samples. Using the estimated empirical distributions, we computed adjusted *P*-values for the two tests for each gene and then combined these *P*-values using Stouffer’s method^59^. Finally, we identified DEGs as those genes showing combined *P*-values ≤ 0.05 and absolute log_2_ fold changes ≥ 0.58 (1.5-fold). To identify the cellular processes represented by the DEGs, we performed enrichment analysis of gene ontology biological processes (GOBPs) using DAVID software^60^ and selected the GOBPs with *P*-values < 0.05 as the processes enriched by the DEGs.

### Reconstruction of a network model

To construct a network model for the DEGs, we first selected a subset of the DEGs involved in the GOBPs related to mTOR signalling (Supplementary Fig. 9a, b) and glucose and fatty acid metabolism (Fig. 5c). We then collected the protein–protein interactions for these genes from five interactome databases: Biomolecular Interaction Network Database, Human Protein Reference Database, Biological General Repository for Interaction Datasets, Molecular INTeraction Database and Search Tool for Recurring Instances of Neighbouring Genes^61–65^. The initial network model was built with the target genes and their interactors using Cytoscape^66^. We then arranged the nodes according to their associated GOBPs and Kyoto Encyclopedia of Genes and Genomes pathways^67^ related to glucose and fatty acid metabolism, such that the nodes involved in the same GOBPs and pathways were located close to each other.

### Data availability

Uncropped gels and blots are available in the Supplementary Information (Supplementary Figs. 13–22).

RNA-seq data generated for this study have been deposited in the Gene Expression Omnibus database under accession number GSE115012.

## Electronic supplementary material

Supplementary Information

Peer Review

Description of Additional Supplementary Files

Supplementary Data 1
