# Supplementary material for: Loss of the E3 ubiquitin ligase MKRN1 represses diet-induced metabolic syndrome through AMPK activation
Source: Nat Commun. 2018 Aug 24;9:3404. doi: 10.1038/s41467-018-05721-4 (PMC6109074; doi:10.1038/s41467-018-05721-4)
Supplement: Supplementary file 1 — Supplementary Information [file 41467_2018_5721_MOESM1_ESM.pdf]

Supplementary Information

Supplementary Methods

Supplementary Figures 1-22

Supplementary References

Loss of the E3 ubiquitin ligase MKRN1 represses diet-induced metabolic syndrome through AMPK activation

Lee & Han et al. 2018

Supplementary Methods

Plasmids and siRNAs

pcDNA3.1-MKRN1, pcDNA3-HA-MKRN1 and pGEX4T-1-GST-MKRN1 WT/H307E were described previously[1]. AMPK cDNA was purchased from Addgene (Cambridge, MA, USA) and subcloned into pcDNA3-FLAG. The pRK5-HA-Ub construct was provided by B. J. Hwang (Kangwon University, Korea).

All the siRNAs were obtained from Qiagen (Valencia, CA, USA), and the sequences were as follows: human MKRN1 #6, 5′-CGGGATCCTCTCCAACTGCAA-3′; #7, 5′-CACAGGCG AAGCTGAGTCAAG-3′; human AMPKα1, 5′-TCGGGATCAGTTAGCAACTAT-3′; human AMPKα2, 5′-CCGAAGTCAGAGCAAACCGTA-3′; human PTEN, 5′-TCGGCTTCTCCTG AAAGGGAA-3′; mouse AMPKα1, 5′-CACGAGTTGACCGACATAAA-3′; and mouse AMPKα2, 5′-CAGGGAAGCCTTAAATATTTA-3′.

Real-time qPCR analysis

RNA was obtained from tissues and cells using the TRIzol reagent according to the manufacturer’s instructions (Invitrogen, Carlsbad, CA, USA). cDNA was amplified using 1 μg of total RNA and analysed using the QuantiTect SYBR Green PCR Kit and real-time PCR (Rotor-GeneQ 2plex, Qiagen) with custom Primetime qPCR Primers (IDT, Coralville, IA, USA). The primer pairs were as follows: human PRKAA1/AMPKα1, 5′-TGGAAGGTCCTG TTTAAACCA-3′ and 5′-TTTAAATCCTTCTGTGATTAGCCTTT-3′; human PRKAA2/ AMPKα2, 5′-CCAACAACATCTAAACTGCGAA-3′ and 5′-GTGAAGATCGGACACTACG TG-3′; human Gapdh, 5′-TGTAGTTGAGGTCAATGAAGGG-3′ and 5′-ACATCGCTCAGA CACCATG-3′; mouse PRKAA1/AMPKα1, 5′-CCTGCGTACAATCTTCCTGAA-3′ and 5′-T GAATGCAAAGATAGCCGACT-3′; mouse PRKAA2/ AMPKα2, 5′-GACTTCCTTCACAG

CCTCATC-3′ and 5′-CGAGCGACTATCAAAGACATACG-3′; mouse Glut1, 5′-GCTTCT CCAACTGGACCTCAAAC-3′ and 5′-ACGAGGAGCACCGTGAAGATGA-3′; mouse Glut4, 5′-GGTGTGGTCAATACGGTCTTCAC-3′ and 5′-AGCAGAGCCACGGTCATCAA GA-3′; mouse Hk2, 5′-CCCTGTGAAGATGTTGCCCACT-3′ and 5′-CCTTCGCTTGCCATT ACGCACG-3′; mouse Srebp1, 5′-GTCACTGTCTTGGTTGATG-3′ and 5′-CGAGATGTGC GAACTGGAC-3′; mouse Fasn, 5′-ACTCCTGTAGGTTCTCTGACTC-3′ and 5′-GCTCCTC GCTTGTCGTC-3′; mouse ChREBP, 5′-CCAGCCTCAAGGTGAGCAAA-3′ and 5′- CATGT CCCGCATCTGGTCA-3′; mouse SCD, 5′-AGATCTCCAGTTCTTACACGA CCAC-3′ and 5′-GACGGATGTCTTCTTCCAGGTG-3′; mouse PPARα, 5′-CCAGCTTCAGCCGAATAGT TC-3′ and 5′-GTGCCCTGAACATCGAGTG-3′; mouse FADS1, 5′-ACCTGTCAGTCTTTG GCACCTC-3′ and 5′-TCCTTGCGGAAGCAGTTAGGCT-3′; mouse FADS2, 5′-TTCCTGG AGAGCCACTGGTTTG-3′ and 5′-GAAGAAGGACTGCTCCACATTGC-3′; mouse Ppargc1α/PGC-1α, 5′-GAATCAAGCCACTACAGACACCG-3′ and 5′-CATCCCTCTTGAG CCTTTCGTG-3′; mouse G6Pase, 5′-GGAGGCTGGCATTGTAGATG-3′ and 5′-TCTACCTT GCTGCTCACTTTC-3′; mouse Tnf, 5′-TCTTTGAGATCCATGCCGTTG-3′ and 5′-AGACC CTCACACTCAGATCA-3′; mouse Prdm16, 5′-ATCCACAGCACGGTGAAGCCAT-3′ and 5′ -ACATCTGCCCACAGTCCTTGCA-3′; mouse Cidea, 5′-GGTGGACACAGAGGAGTTCT TTC-3′ and 5′-CGAAGGTGACTCTGGCTATTCC-3′; and mouse β-actin, 5′-GTACGACCA GAGGCATACAG-3′ and 5′-CTGAACCCTAAGGCCAACC-3′. The mRNA levels were normalized by human Gapdh and mouse β-actin levels.

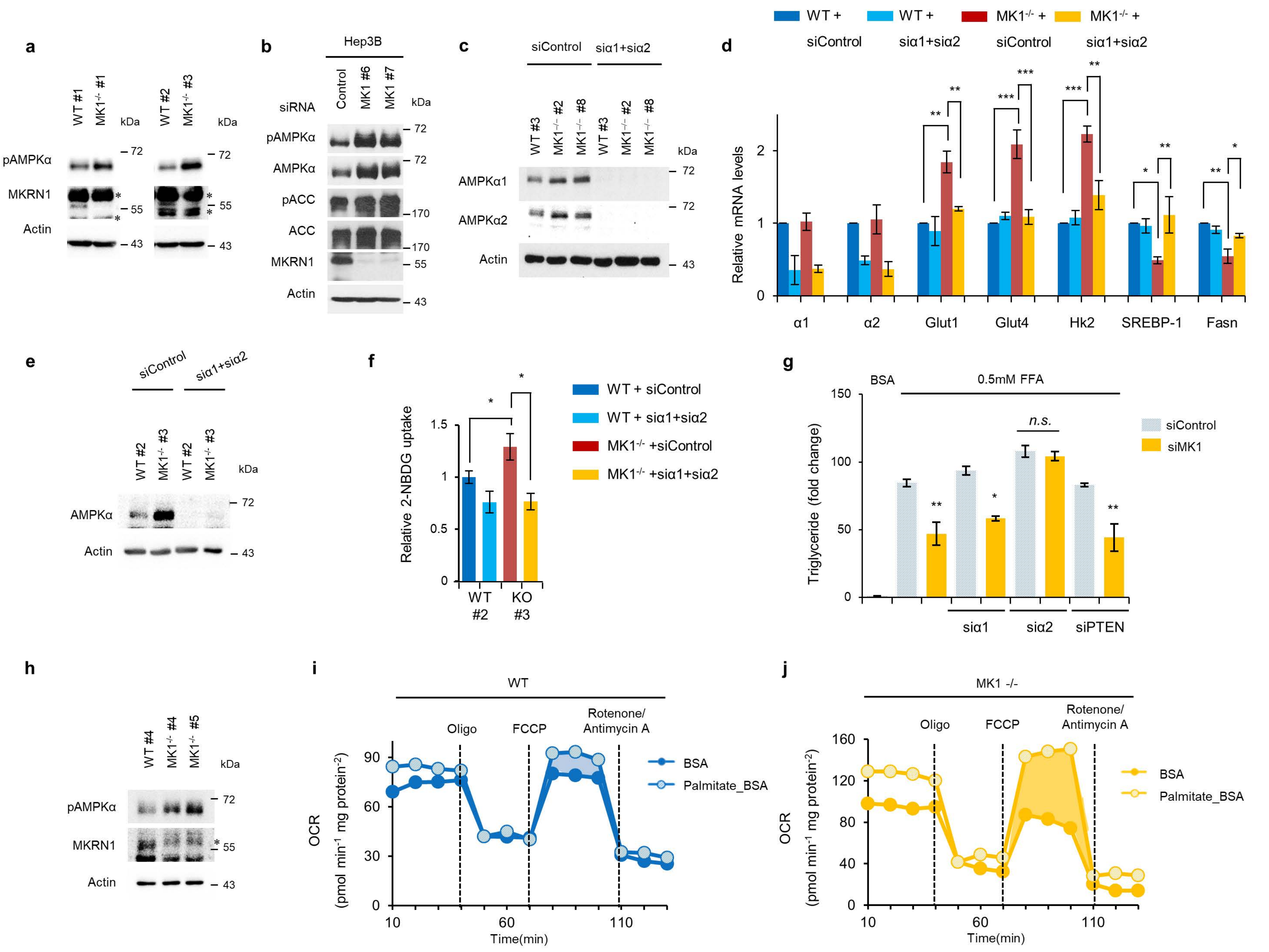

a
WT #1
MK1-/- #1
WT #2
MK1-/- #3
kDa
pAMPKα
MKRN1
Actin
72
55
43
b
Hep3B
siRNA
Control
MK1 #6
MK1 #7
pAMPKα
AMPKα
pACC
ACC
MKRN1
Actin
170
c
siControl
siα1+siα2
WT #3
MK1-/- #2
MK1-/- #8
AMPKα1
AMPKα2
d
WT + siControl
WT + siα1+siα2
MK1-/- + siControl
MK1-/- + siα1+siα2
Relative mRNA levels
α1
α2
Glut1
Glut4
Hk2
SREBP-1
Fasn
e
AMPKα
f
Relative 2-NBDG uptake
WT #2
KO #3
WT + siControl
WT + siα1+siα2
MK1-/- +siControl
MK1-/- +siα1+siα2
g
BSA
0.5mM FFA
Triglyceride (fold change)
n.s.
siControl
siMK1
siα1
siα2
siPTEN
h
WT #4
MK1-/- #4
MK1-/- #5
i
WT
Oligo
FCCP
Rotenone/ Antimycin A
OCR (pmol min-1 mg protein-2)
Time(min)
BSA
Palmitate_BSA
j
MK1 -/-

Supplementary Figure 1. Effect of MKRN1 deficiency on AMPK signalling and FFA-induced cellular steatosis.
(**a, h**) Depletion of MKRN1 activates AMPKα. The activity of AMPKα was analysed in wild-type (WT) or *MKRN1* knockout (*MK1*$^{-/-}$) littermate primary mouse embryonic fibroblasts (MEFs). The cell lysates were immunoblotted with antibodies for phospho-AMPKα (pAMPKα), MKRN1 and actin.
(**b**) Hep3B cells (p53-deficient) were transduced with 20 nM of MKRN1 siRNAs #6 and #7 or control siRNA for 48 hr as indicated. The cell lysates were immunoblotted with antibodies for pAMPKα, AMPKα, phospho-ACC (pACC), ACC, MKRN1 and actin.
(**c, e**) WT and *MK1*$^{-/-}$ MEFs were transfected with 30 nM of mouse AMPKα1 and α2 siRNAs. After 48 hr, the cell lysates were immunoblotted with antibodies for AMPKα1, α2, α and actin.
(**d**) WT or *MK1*$^{-/-}$ MEFs were transfected with 20 nM of mouse AMPKα siRNA for 48 hr. mRNA expression was analysed with specific primers for glycolytic or lipogenic enzymes as indicated by quantitative real-time PCR.
(**f**) Under conditions identical to those from Supplementary Figure 1d, the amount of glucose used was measured by quantifying of 2-NBDG uptake. The cells were treated with 20 μM of 2-NBDG for 30 min and were then analysed using flow cytometry.
(**g**) HepG2 cells were transfected with 20 nM of MKRN1 or 20 nM of PTEN siRNA with or without 20 nM of AMPKα siRNAs, as indicated. The transfected cells were treated with 0.5 mM of FFAs for 24 hr and harvested at 72 hr post-transfection. Cellular TG levels were measured via a colorimetric assay.
(**i, j**) To measure the oxygen consumption rate (OCR), WT or *MK1*$^{-/-}$ MEFs were sequentially treated with oligomycin, FCCP and rotenone/antimycin A in the presence of bovine serum albumin either alone (BSA) or conjugated to palmitate (palmitate-BSA). O_2 consumption was measured per minute with an XF-Analyzer, and the results were normalized to the amount of protein.
The data are presented as the mean ± standard deviation (s.d.) of triplicate samples in at least three independent experiments. *$P \leq 0.05$, **$P \leq 0.01$, ***$P \leq 0.001$, *n.s.*, not significant. Please ensure that the intended meaning has been maintained in this edit.

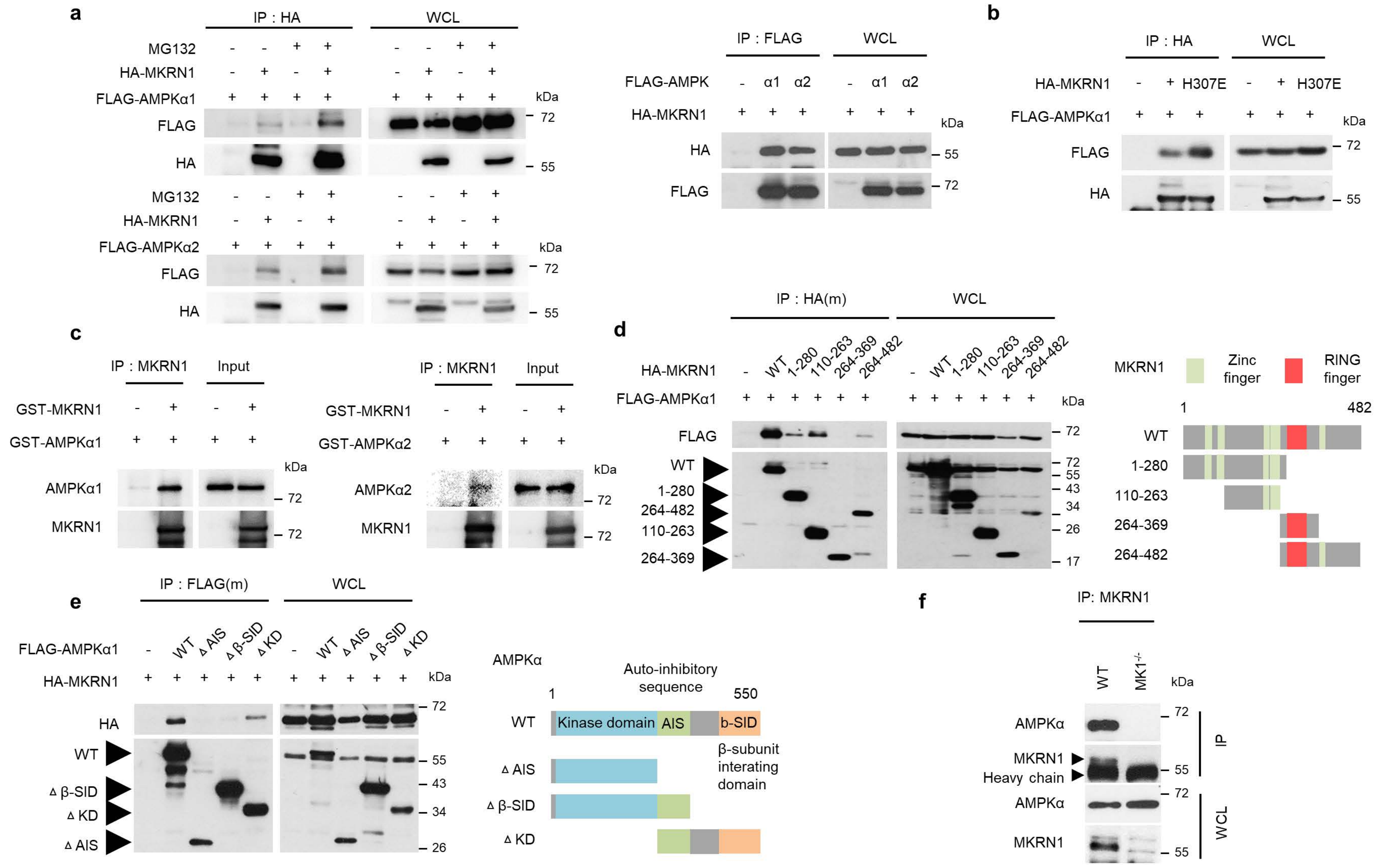

a
IP : HA
WCL
MG132
HA-MKRN1
FLAG-AMPKα1
FLAG-AMPKα2
FLAG
HA
kDa
72
55
IP : FLAG
FLAG-AMPK
α1
α2
b
H307E
c
IP : MKRN1
Input
GST-MKRN1
GST-AMPKα1
GST-AMPKα2
AMPKα1
AMPKα2
MKRN1
d
IP : HA(m)
WT
1-280
110-263
264-369
264-482
Zinc finger
RING finger
1
482
e
IP : FLAG(m)
Δ AIS
Δ β-SID
Δ KD
AMPKα
Auto-inhibitory sequence
550
Kinase domain
AIS
b-SID
β-subunit interating domain
f
IP: MKRN1
MK1-/-
Heavy chain
IP

Supplementary Figure 2. The E3 ubiquitin ligase MKRN1 interacts with AMPK a subunits.
(**a**, **b**) Constructs expressing FLAG-MKRN1 FLAG-MKRN1 H307E and HA-AMPKα1 and α2 were transfected into 293T cells, as indicated. After 24 hr, the transfected cells were treated with 20 μM of MG132 for 5 hr, and immunoprecipitation assays were then performed using the cell lysates. AMPKα1, α2 or MKRN1 was immunoprecipitated using antibodies for HA and FLAG. MKRN1 WT, H307E and AMPKα1 and α2 were detected using antibodies for FLAG and HA.
(**c**) GST-MKRN1 and AMPKα1 (left) and α2 (right) proteins purified from bacteria were incubated and immunoprecipitated with an MKRN1 antibody. The lysates were immunoblotted with antibodies for MKRN1 and AMPKα.
(**d**) Plasmids containing FLAG-AMPKα1, HA-MKRN1 and various domains of MKRN1 were transduced into 293T cells as indicated. Then, MKRN1 and its various domains were immunoprecipitated with an antibody for HA. AMPKα1, MKRN1 and various domains of MKRN1 were detected with antibodies for FLAG and HA.
(**e**) Reciprocally, HA-MKRN1, FLAG-AMPKα and various domains of AMPKα1 were expressed in 293T cells. AMPKα1 was immunoprecipitated with an antibody for FLAG, and each of the proteins was detected with an antibody for FLAG or HA.
(**f**) Cell lysates of WT and *MK1*$^{-/-}$ MEFs were immunoprecipitated using an antibody for MKRN1. AMPKα and MKRN1 were detected with specific antibodies for MKRN1 and AMPKα.

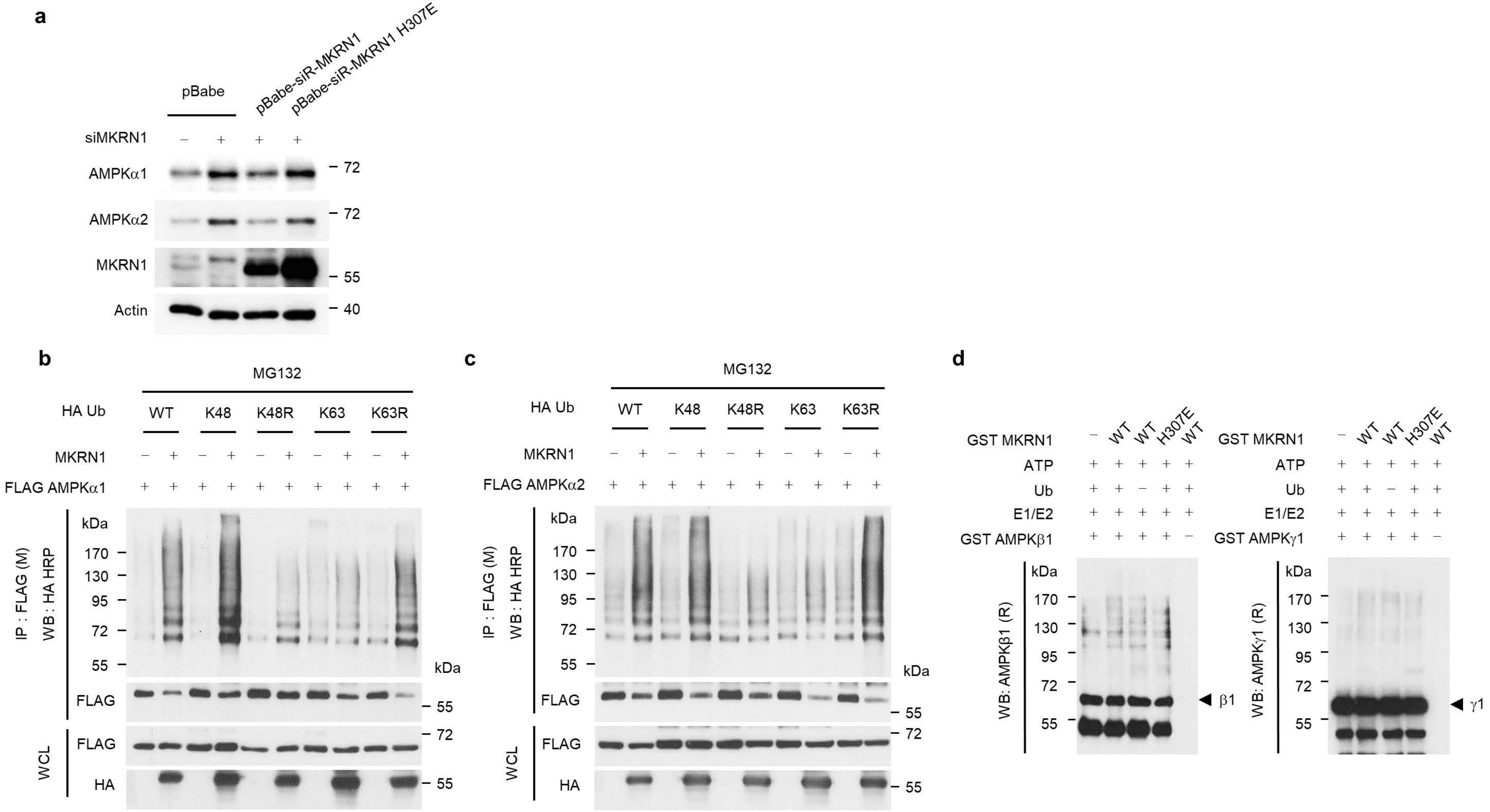

Supplementary Figure 3. MKRN1 ubiquitinates AMPKα, but not AMPKβ or γ via K48 linkage.
(**a**) HepG2 cells were infected with retroviruses expressing siRNA-resistant MKRN1 WT and H307E, and the cells were then transfected with 20 nM of siRNA for 48 hr. The cell lysates were detected with specific antibodies for AMPKα1, α2, MKRN1 and actin. (**b**, **c**) 293T cells were transfected with the indicated plasmids along with HA-tagged Ub (WT) or the Ub-K48-only mutant (K48), Ub-K48R mutant (mutation of lysine 48 to arginine), Ub-K63-only mutant (K63) or Ub-K63R mutant (mutation of lysine 63 to arginine). AMPKα1 and α2 were immunoprecipitated using an antibody for FLAG. Ubiquitinated AMPK was identified using an HA-HRP antibody under denaturing conditions. (**d**) Recombinant MKRN1 and the H307E mutant (enzymatically defective mutant MKRN1 protein) as well as the AMPKβ1 and γ1 subunits were incubated with E1, E2, ubiquitin (Ub) and ATP for 3 hr at 37 °C, as indicated, to determine the in vitro ubiquitination of AMPK.

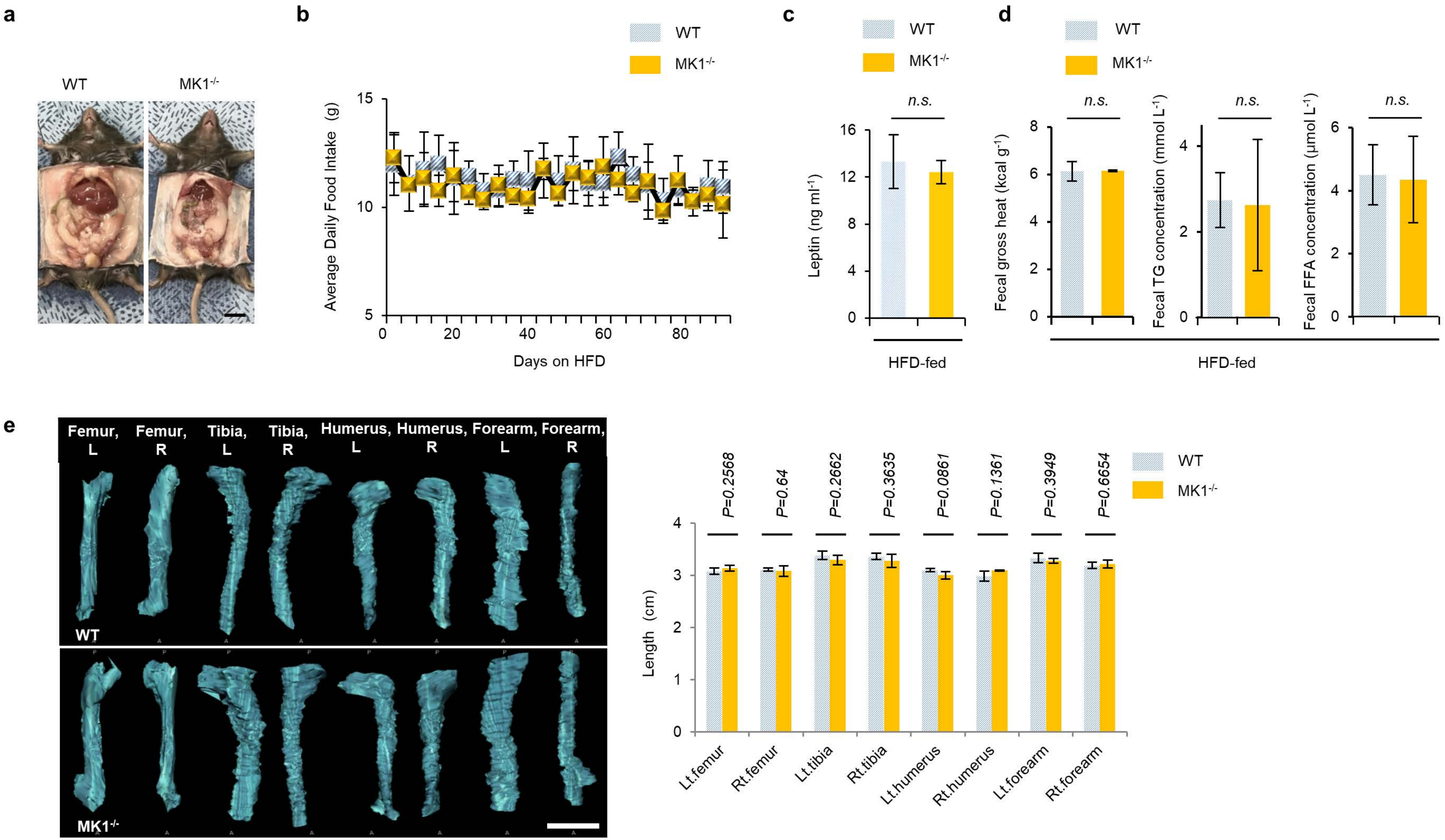

Supplementary Figure 4. Effects of MKRN1 depletion on energy intake and bone length.
(**a**-**d**) *MKRN1* knockout has no effect on energy intake in HFD-fed male mice. (**a**) Photographs show the abdominal cavities of representative male mice from each group after HFD treatment for 16 weeks. Scale bar = 1 cm. (**b**) Average daily food intake. The amounts of HFD consumed by WT and *MK1*$^{-/-}$ mice were measured every 4 days during the HFD-feeding period. (**c**) Plasma leptin levels in HFD-fed, overnight-fasted mice (WT *n* = 5 and *MK1*$^{-/-}$ *n* = 6). (**d**) Unabsorbed energy was determined based on the measured faecal gross heat and the levels of faecal TG and FFA from HFD-fed WT and *MK1*$^{-/-}$ mice. (WT *n* = 6 and *MK1*$^{-/-}$ *n* = 8).
(**e**) Representative micro-CT images (left) and bone lengths of the left (L) and right (R) femurs, tibia, humerus and forearm from mice fed an HFD. (*n* = 3 mice per group). Scale bar = 1 cm.
The data in **c**, **d, e** and **f** are presented as the mean ± s.d. Two-tailed Student's *t*-test for **c, d, e** and **f**, *n.s.*, not significant.

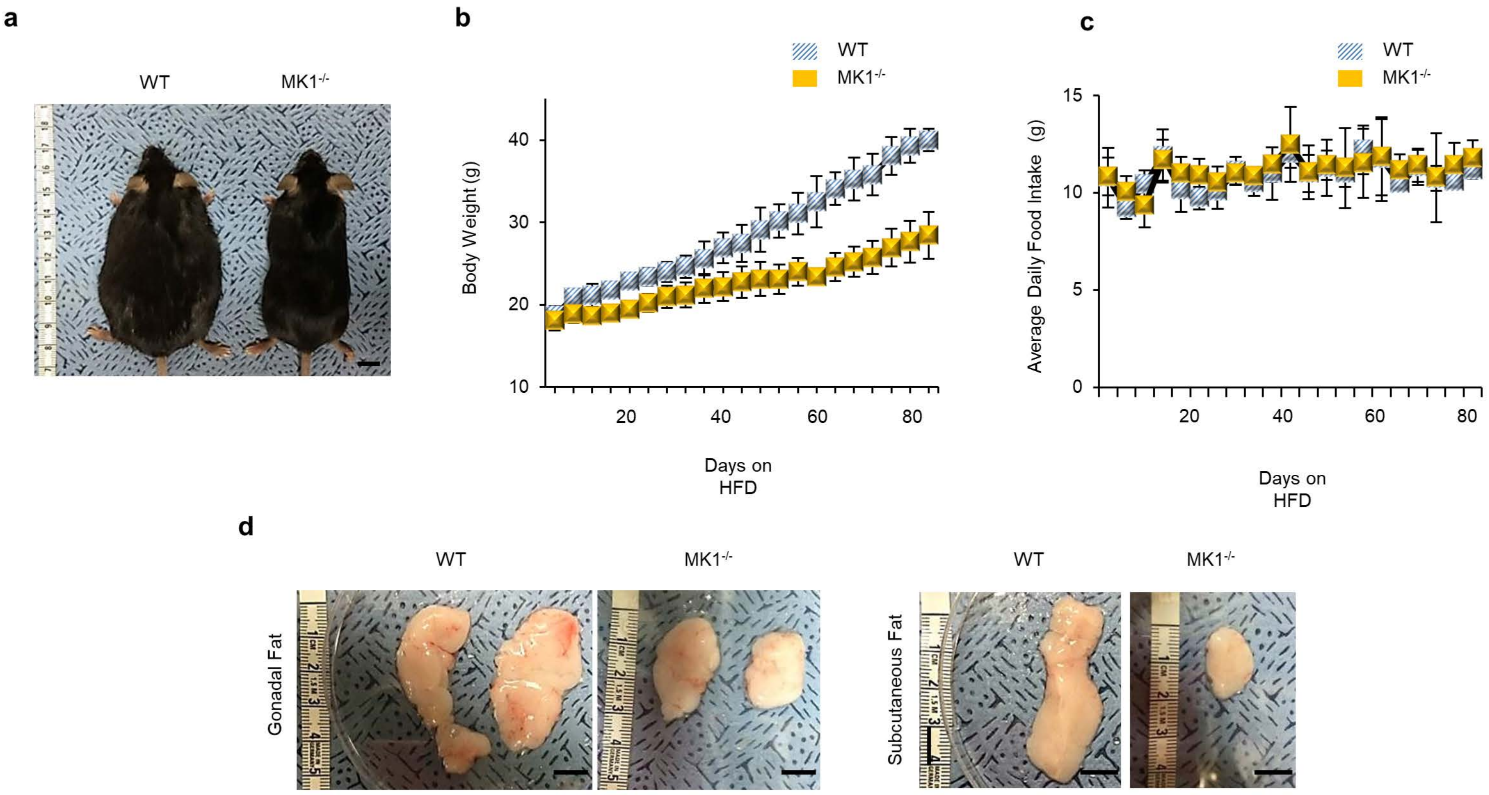

Supplementary Figure 5. Female *MKRN1*-null mice display resistance against HFD-induced weight gain.
(**a-d**) Six- to seven-week-old female WT and $MK1^{-/-}$ mice were fed an HFD for 16 weeks. (**a**) Representative images of female WT and $MK1^{-/-}$ mice fed an HFD. Scale bar = 1 cm. (**b**, **c**) Body weights (**b**) and food intake (**c**) were recorded every 4 days for WT and $MK1^{-/-}$ female mice fed an HFD (WT n = 7 and $MK1^{-/-}$ n = 8). The data are presented as the mean ± s.d. (**d**) $MK1^{-/-}$ female mice exhibited a reduced fat-pad size. Representative images of gonadal (left) and subcutaneous fat (right) from female WT and $MK1^{-/-}$ mice fed an HFD. Scale bar = 1 cm.

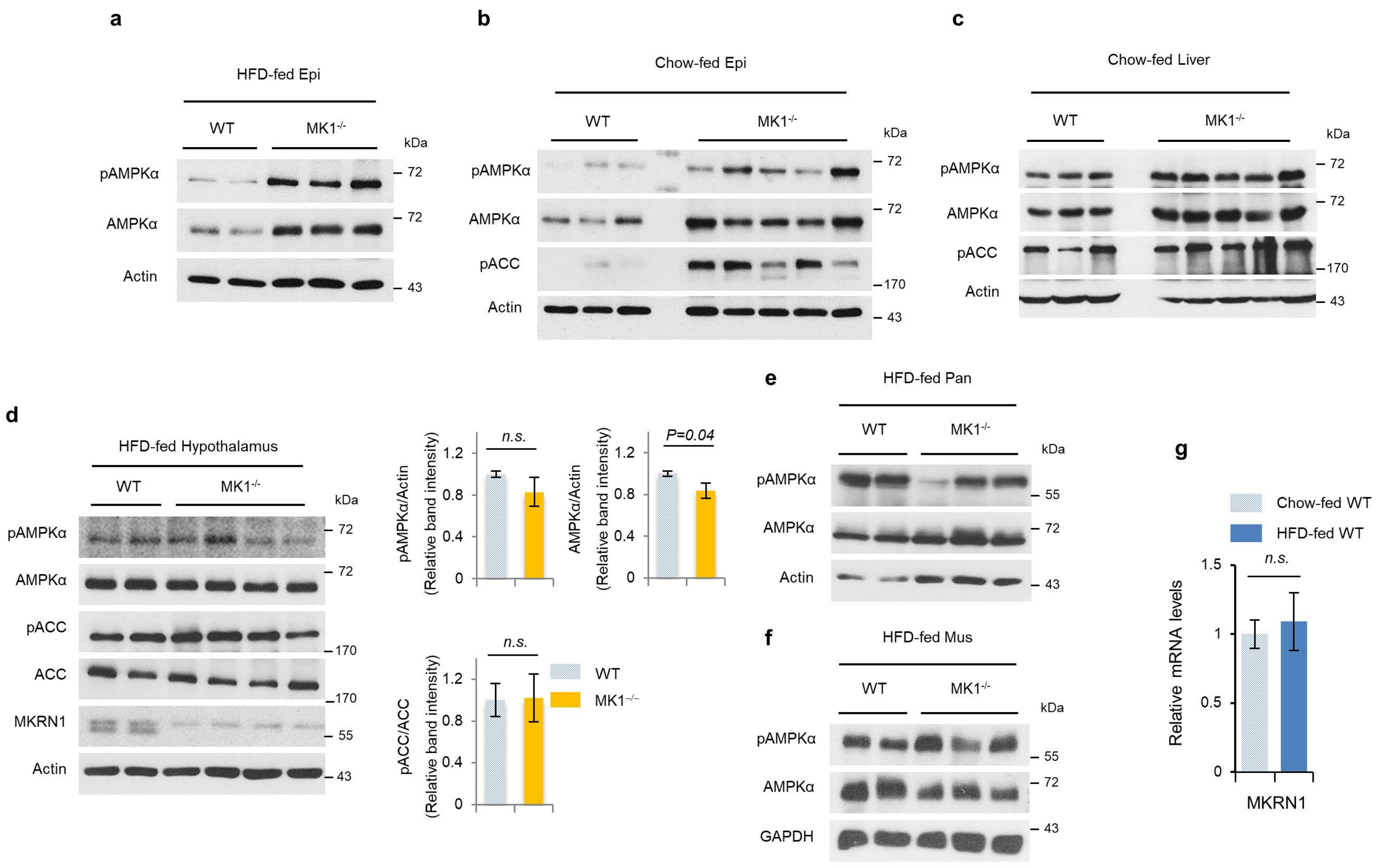

Supplementary Figure 6. A lack of MKRN1 expression induces stabilization and activation of AMPK in the liver and adipose tissue.

(**a**-**f**) Male WT and *MK1*$^{-/-}$ mice fed a normal chow diet or HFD for 16 weeks. Lysates of epididymal fat (**a**, **b**), liver (**c**), hypothalamus (**d**), pancreas (**e**) or skeletal muscle (**f**) tissues were analysed by immunoblotting with antibodies for pAMPKα, AMPKα, pACC, ACC, MKRN1 and actin (*n* = 2-5 mice per group). (**d**) The relative band intensity of pAMPKα, AMPKα and pACC was quantified using the ImageJ program and normalized to the band intensity for actin and ACC.

(**g**) Standard chow- and HFD-fed mice liver lysates were prepared for MKRN1 mRNA extraction. The mRNA expression of MKRN1 was analysed by quantitative real-time PCR with a specific primer for MKRN1 (standard chow-fed *n* = 4 and HFD-fed *n* = 6).

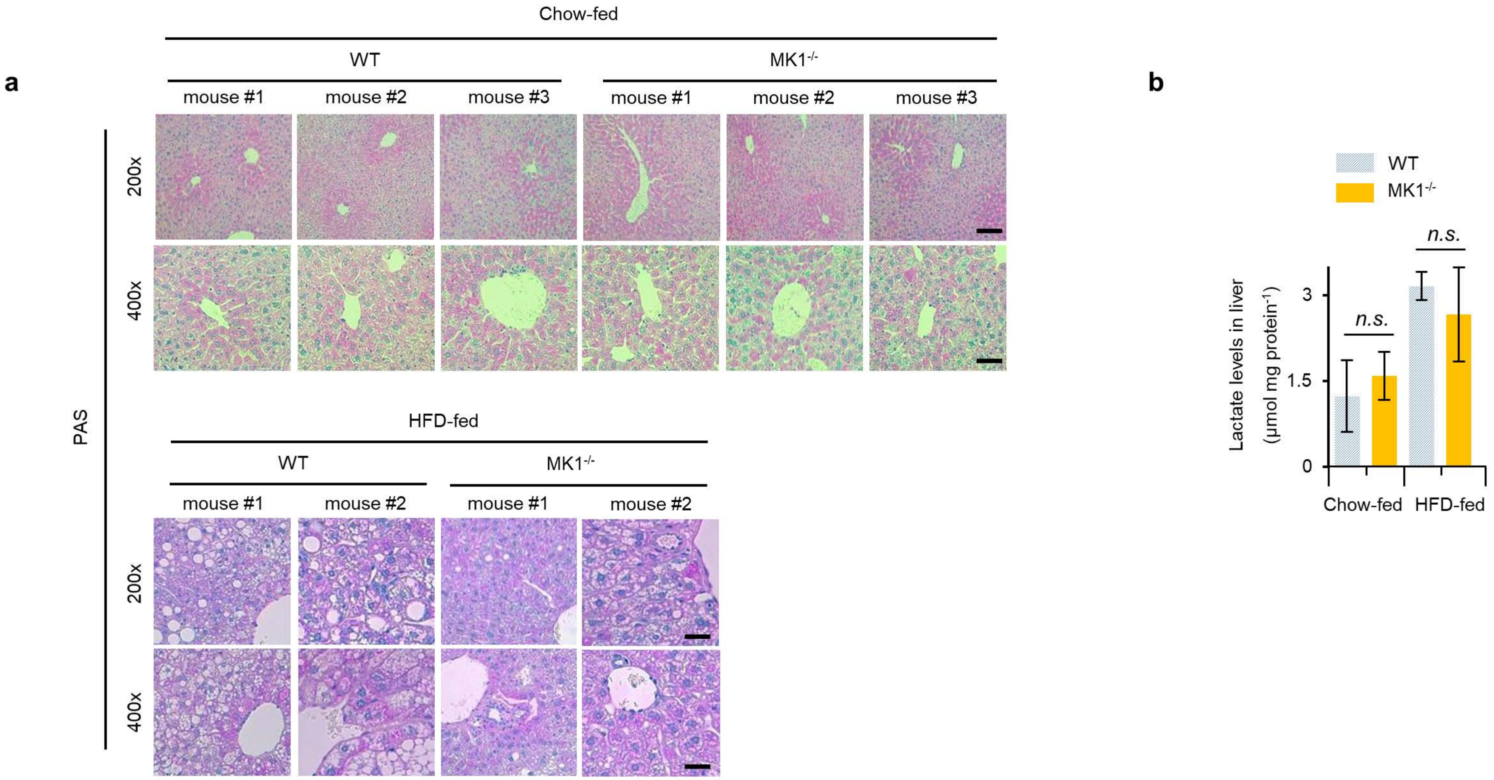

Supplementary Figure 7. No effect of MKRN1 depletion on glycogen accumulation or generation of lactate.
(**a**) Representative images of periodic acid Schiff (PAS)-stained liver tissues of WT and *MK1*$^{-/-}$ mice for the detection of glycogen. *MKRN1* knockout has no effect on glycogen accumulation in the livers of normal chow diet- and HFD-fed mice. Scale bar = 100 μm (top) and 50 μm (bottom).
(**b**) Liver lactate contents were measured in WT or *MK1*$^{-/-}$ liver lysates with or without HFD. The data are presented as the mean ± standard deviation (s.d.) of triplicate samples. *n.s.*, not significant. Scale bar = 100 μm (top) and 50 μm (bottom).

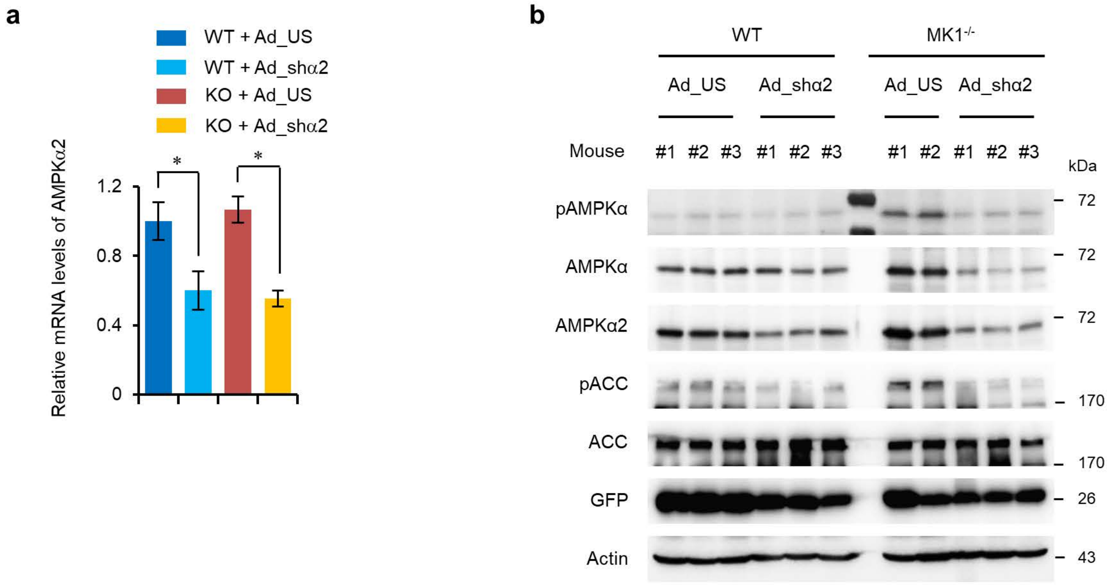

Supplementary Figure 8. MKRN1 deficiency induces activation of AMPK signalling in the liver.
(**a**) Adenovirus-mediated knockdown of hepatic AMPKα2. The mRNA levels of AMPKα2 were analysed by quantitative real-time PCR with a specific primer for AMPKα2. The data are presented as the mean ± standard deviation (s.d.) of triplicate samples in at least three independent experiments. *$P \leq 0.05$, **$P \leq 0.01$, ***$P \leq 0.001$, *n.s.*, not significant.
(**b**) Liver lysates from adenovirus-injected mice were immunoblotted with antibodies for pAMPKα, AMPKα, AMPKα2, pACC, ACC, GFP and actin.

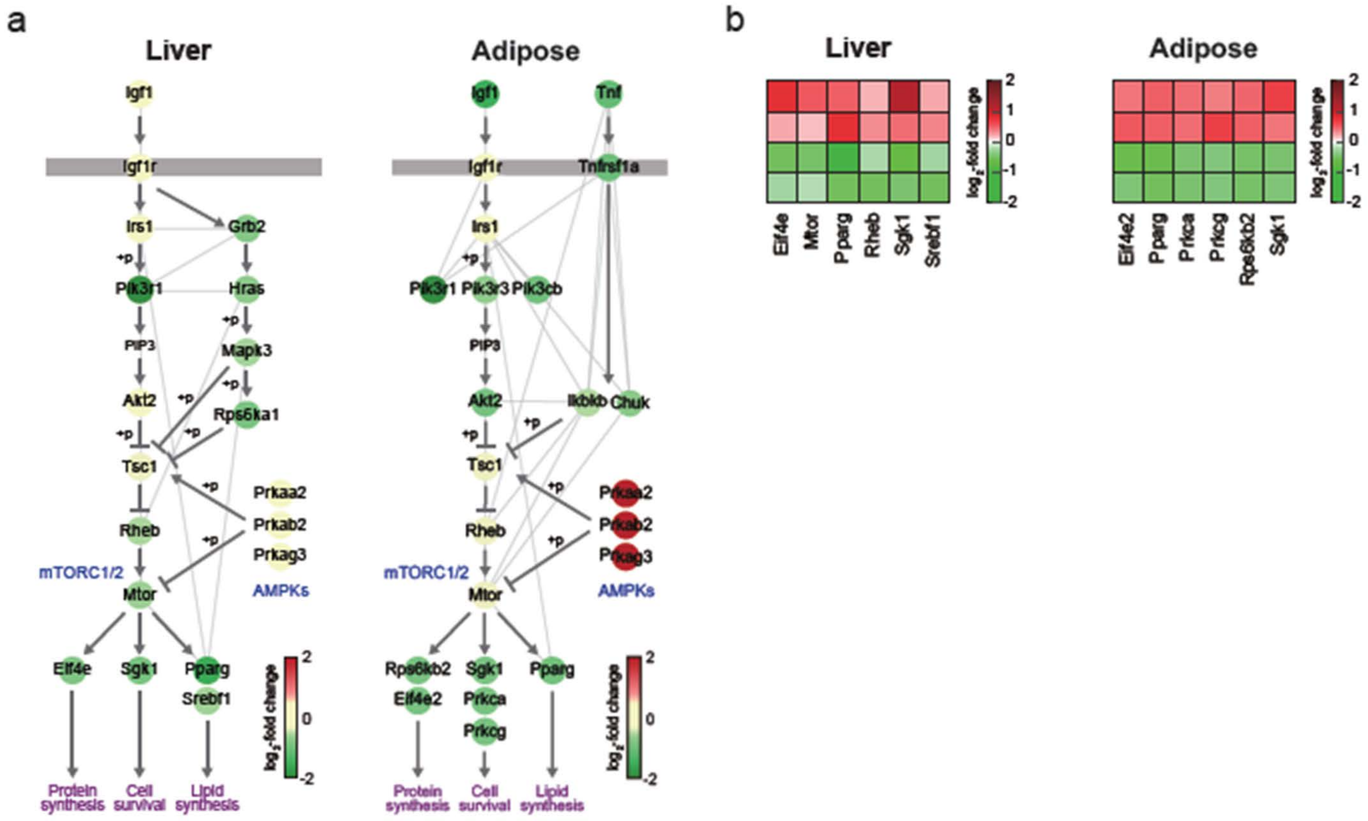

Supplementary Figure 9. Regulation of mTOR signalling by MKRN1 and AMPK.
(**a**,**b**) Network models describing alterations of mTOR signalling in *MKRN1*-knockout liver (left) and adipose tissues (right). Arrows denote positive regulation in signalling cascades, while inhibition symbols denote negative regulation. Node colours represent up- (red) or down-regulation (green) in *MK1*$^{-/-}$ liver or adipose tissues (**a**). The colour bar represents the gradient of the log_2-fold-changes of mRNA expression levels following MKRN1 ablation relative to those in WT (**b**).

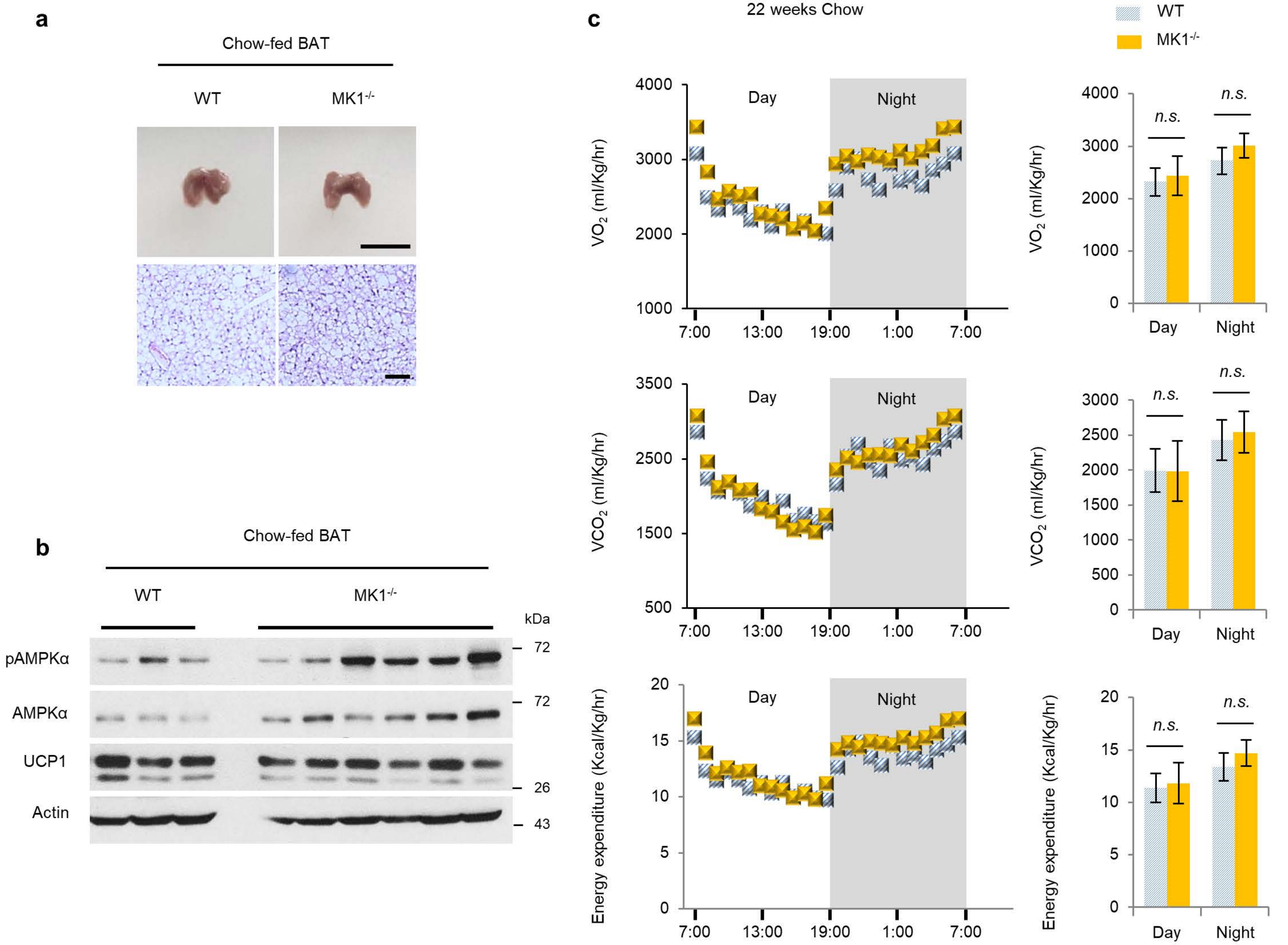

Supplementary Figure 10. MKRN1-deficient mice display near-equal energy expenditure to that of WT mice placed on a normal chow diet.

(**a**) Representative image (top, Scale bar = 1cm) and H&E staining (bottom, Scale bar = 25 μm) of BAT in chow-fed male WT and *MK1*$^{-/-}$ mice at 22 weeks of age.

(**b**) BAT lysates were immunoblotted using antibodies for pAMPKα, AMPKα, UCP1 and actin.

(**c**) Oxygen consumption (VO_2), carbon dioxide production (VCO_2) and energy expenditure during light and dark cycles in mice fed a chow diet over a period of 48 hr (right). Each plot shows the mean of the indicated time points over 2 days. The bar graph represents the average day and night values (right) (WT n = 5 and *MK1*$^{-/-}$ n = 8).

The data are presented as the mean ± s.d. Two-tailed Student's t-test for c, *n.s.*, not significant.

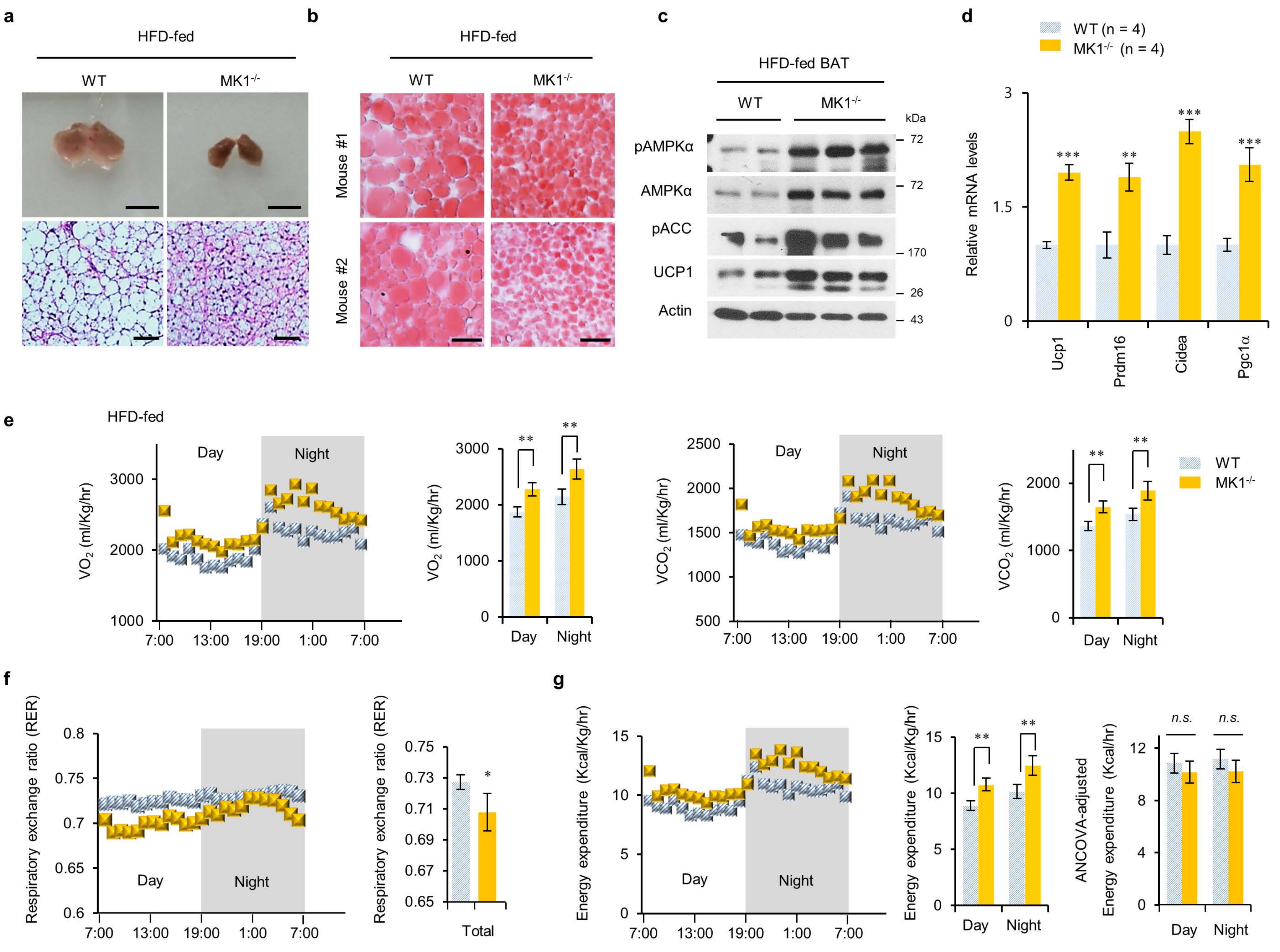

a
HFD-fed
WT
MK1-/-
b
HFD-fed
WT
MK1-/-
Mouse #1
Mouse #2
c
HFD-fed BAT
WT
MK1-/-
kDa
pAMPKα
AMPKα
pACC
UCP1
Actin
72
72
170
26
43
d
WT (n = 4)
MK1-/- (n = 4)
Relative mRNA levels
Ucp1
Prdm16
Cidea
Pgc1α
e
HFD-fed
Day
Night
VO2 (ml/Kg/hr)
VCO2 (ml/Kg/hr)
WT
MK1-/-
f
Respiratory exchange ratio (RER)
Total
g
Energy expenditure (Kcal/Kg/hr)
ANCOVA-adjusted Energy expenditure (Kcal/hr)
n.s.

Supplementary Figure 11. Depletion of MKRN1 promotes BAT-mediated thermogenesis through near-equal energy expenditure compared with WT mice placed on an HFD.

(**a-e**) Six- to seven-week-old male WT and *MK1*$^{-/-}$ mice were fed an HFD for 16 weeks. (**a**) Representative images (top, Scale bar = 1 cm) and H&E staining (bottom, Scale bar = 25 μm) of BAT. (**b**) BAT tissues were stained with Oil Red-O. Scale bar = 50 μm. (**c**) BAT lysates were immunoblotted using antibodies for pAMPKα, AMPKα, UCP1 and actin. (**d**) The relative mRNA expression of genes associated with thermogenesis in BAT was analysed by quantitative real-time PCR using the indicated specific primers (n = 4 mice per group). p-value compared with WT. The data are the mean ± s.d. Two-tailed Student's *t*-test; $^{**}P \leq 0.01$, $^{***}P \leq 0.001$. (**e**) Oxygen consumption (VO_2) (left), carbon dioxide production (VCO_2) (right), (**f**) respiratory exchange ratio (RER) and (**g**) energy expenditure during light and dark cycles in mice fed an HFD over a period of 48 hr (left). Each plot shows the mean of the indicated time points over 2 days. The bar graph represents the average day and night values (right) (WT n = 5 and *MKRN1*$^{-/-}$ n = 8). The data are presented as the mean ± s.d. Two-tailed Student's *t*-test for c, *n.s.*, not significant. Energy expenditure data were analysed by ANCOVA.

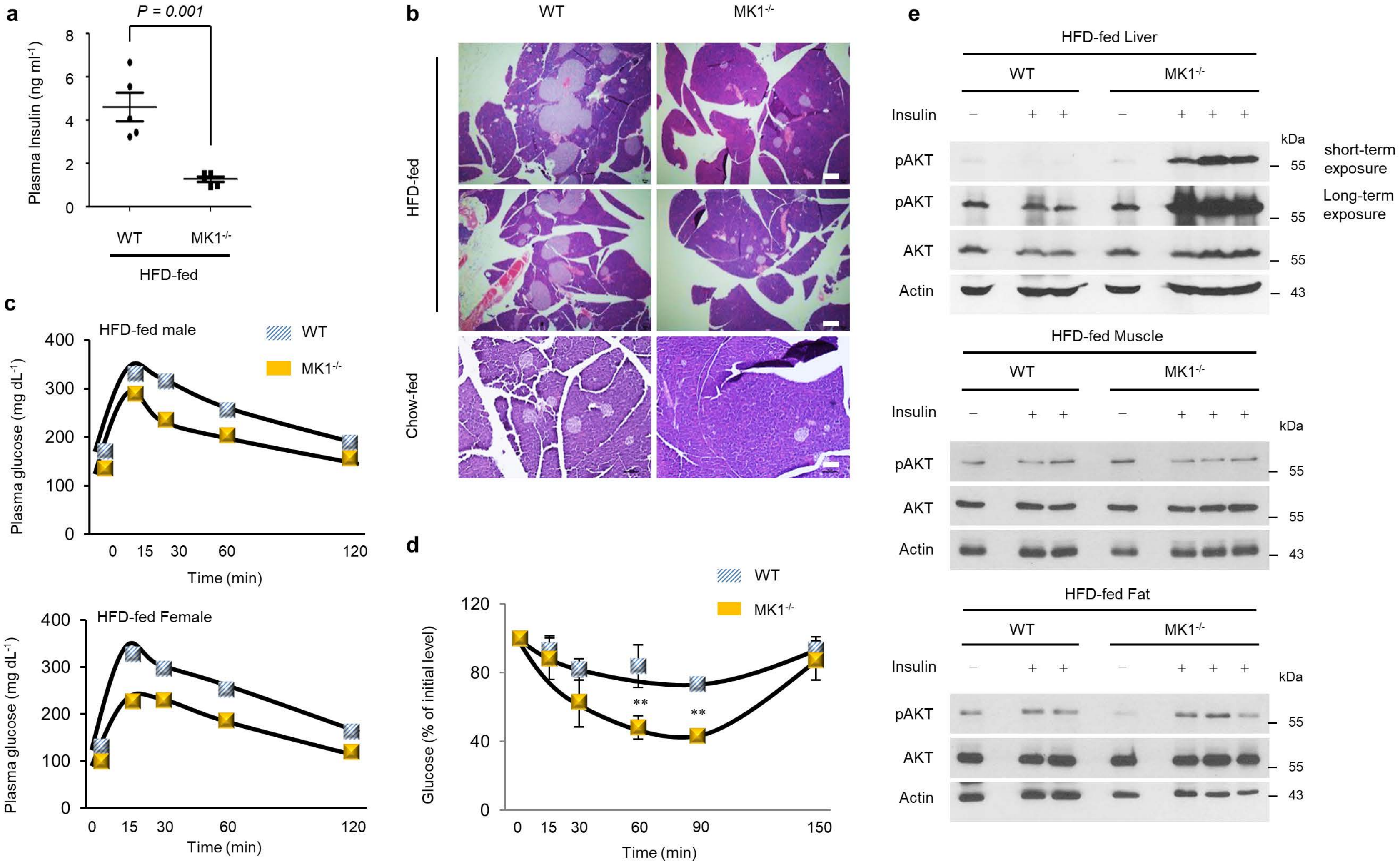

Supplementary Figure 12. Lack of MKRN1 expression prevents insulin resistance associated with an HFD in mice.
(**a-e**) WT and MK1$^{-/-}$ mice maintained on an HFD for 16-18 weeks. (**a**, **b**) *MKRN1* knockout suppresses diet-induced fasting hyper-insulinaemia (**a**) and pancreatic islet hyperplasia (H&E staining of pancreas sections) Scale bar = 100 µm. (**b**).
(**c**) Effect of MKRN1 depletion on glucose intolerance. Glucose tolerance tests (GTTs) were performed using male (top) and female (bottom) mice (*n* = 5 mice per group).
(**d**, **e**) Improved insulin-sensitivity in *MKRN1* knockout mice. Insulin tolerance tests (ITTs) (**d**). The blood glucose concentrations of *MK1*$^{-/-}$ mice and WT controls before the i.p. administration of insulin were set as 100%. (**e**) Immunoblot analysis of insulin-stimulated phosphorylation of AKT in liver, skeletal muscle or WAT. Mice were injected with either saline (-) or insulin (+). Mouse tissue lysates were immunoblotted with antibodies against pAKT, AKT and actin.
The error bars in **c** and **d** indicate s.d. Two-tailed Student's *t*-test for a, c, d; $^{*}P \leq 0.05$, $^{**}P \leq 0.01$

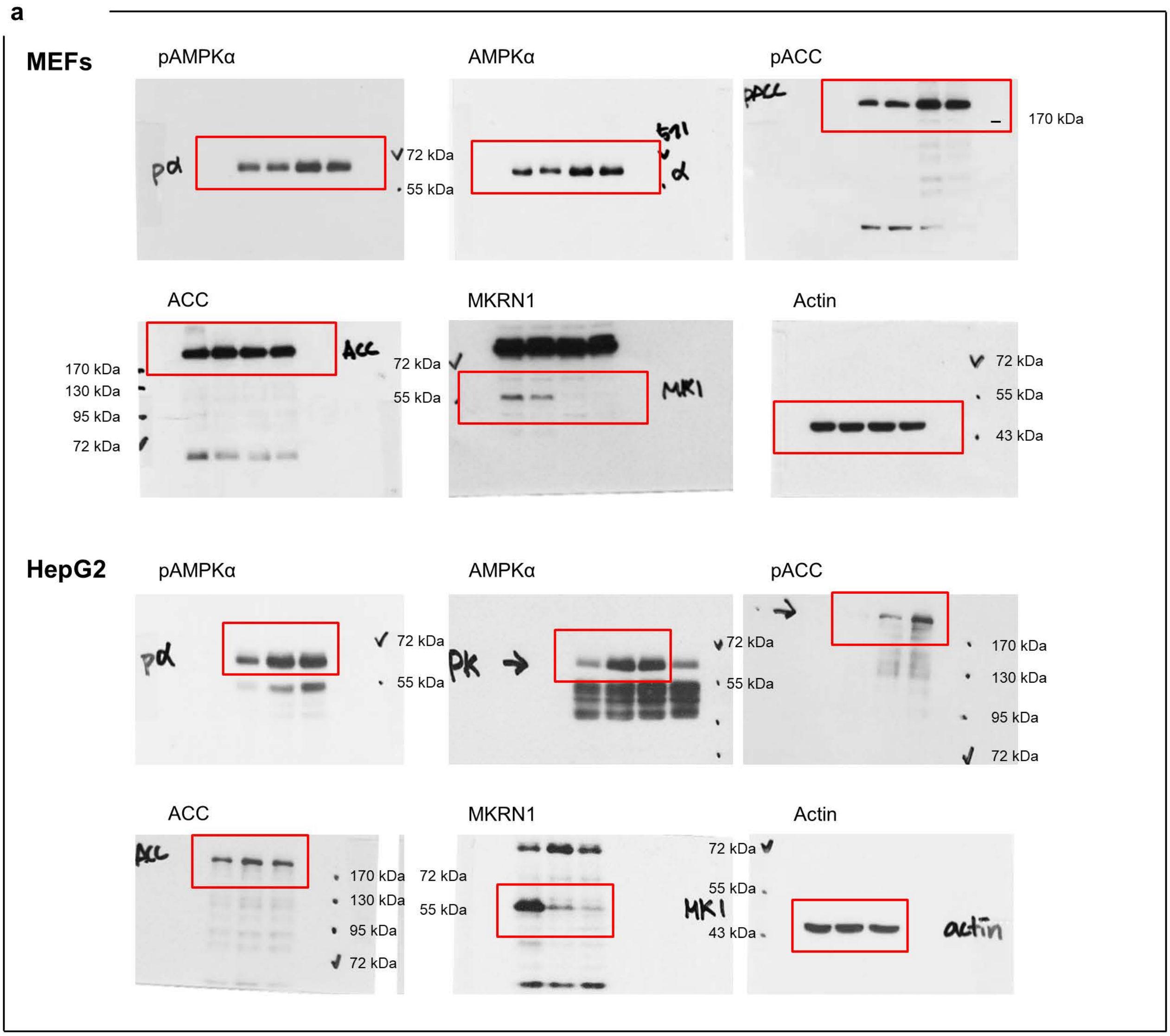

Supplementary Figure 13. Uncropped blots.
(**a**) Uncropped Western blots showing molecular weight markers from Figure 1d (MEFs) and 1f (HepG2 cells).

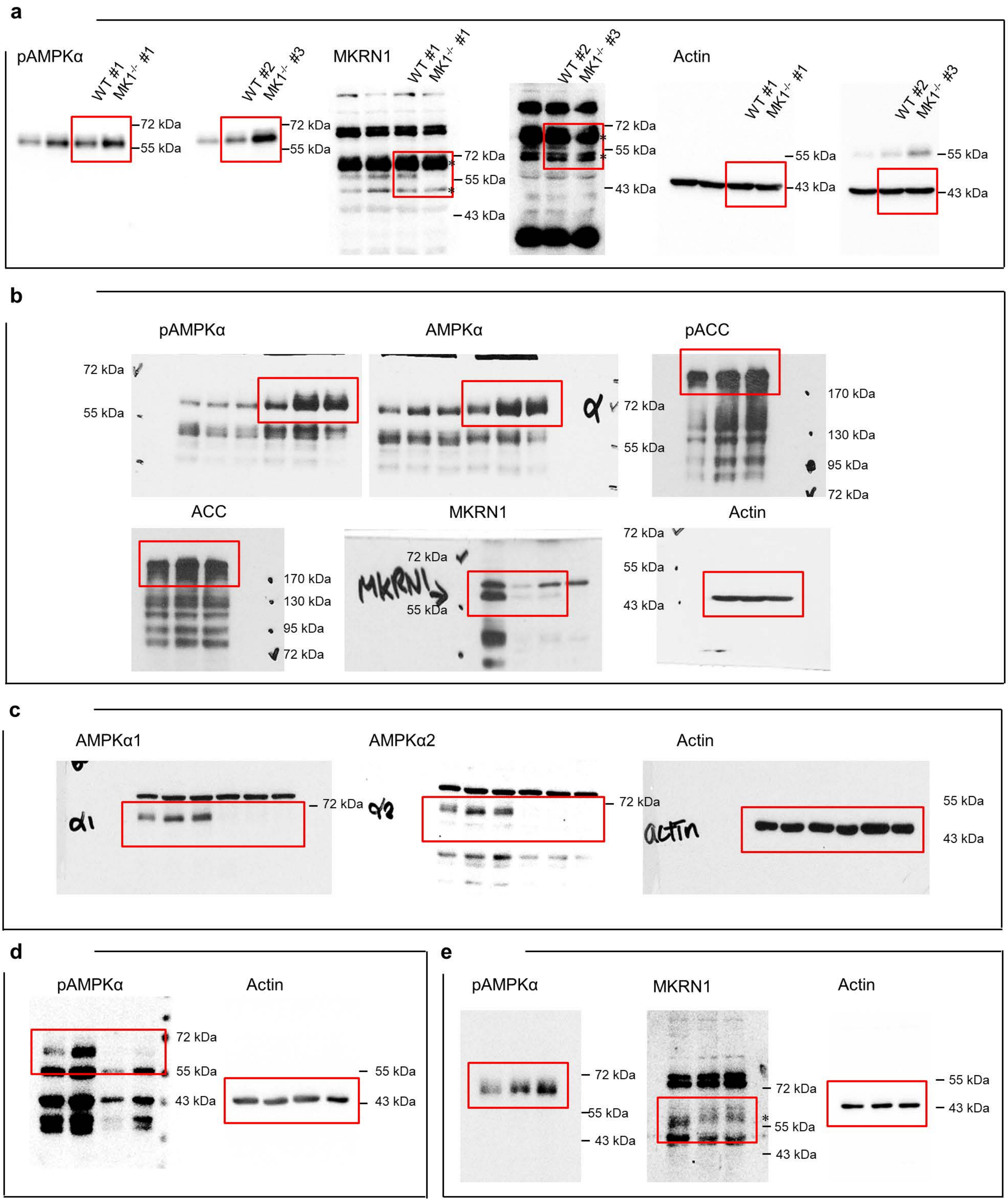

Supplementary Figure 14. Uncropped blots.

(**a-e**) Uncropped Western blots showing molecular weight markers from Supplementary Figure 1a (**a**), 1b (**b**), 1c (**c**), 1e (**d**) and 1h (**e**).

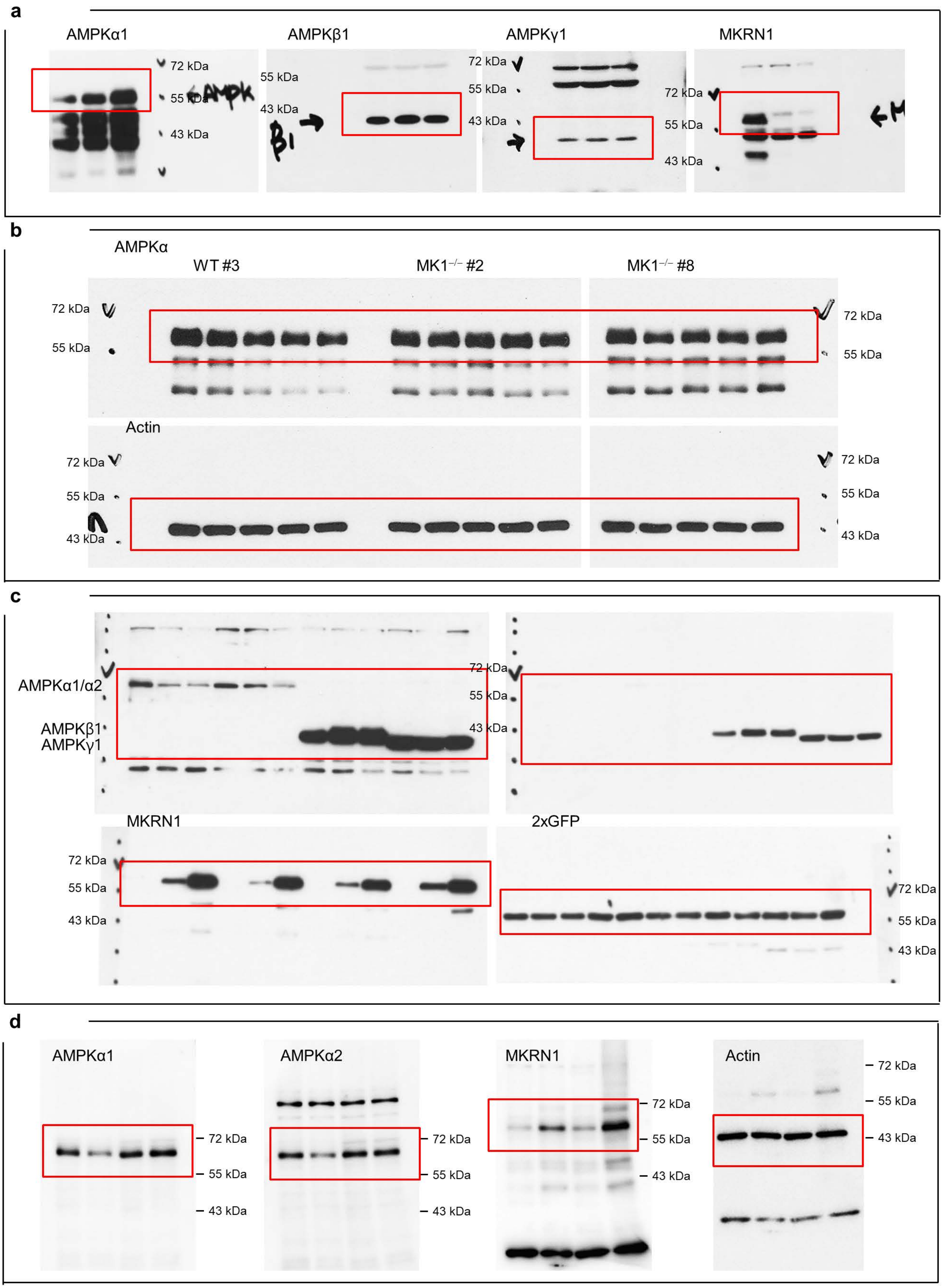

Supplementary Figure 15. Uncropped blots.
(**a-d**) Uncropped Western blots showing molecular weight markers from Figure 2a (**a**), 2b (**b**), 2c (**c**) and 2d (**d**).

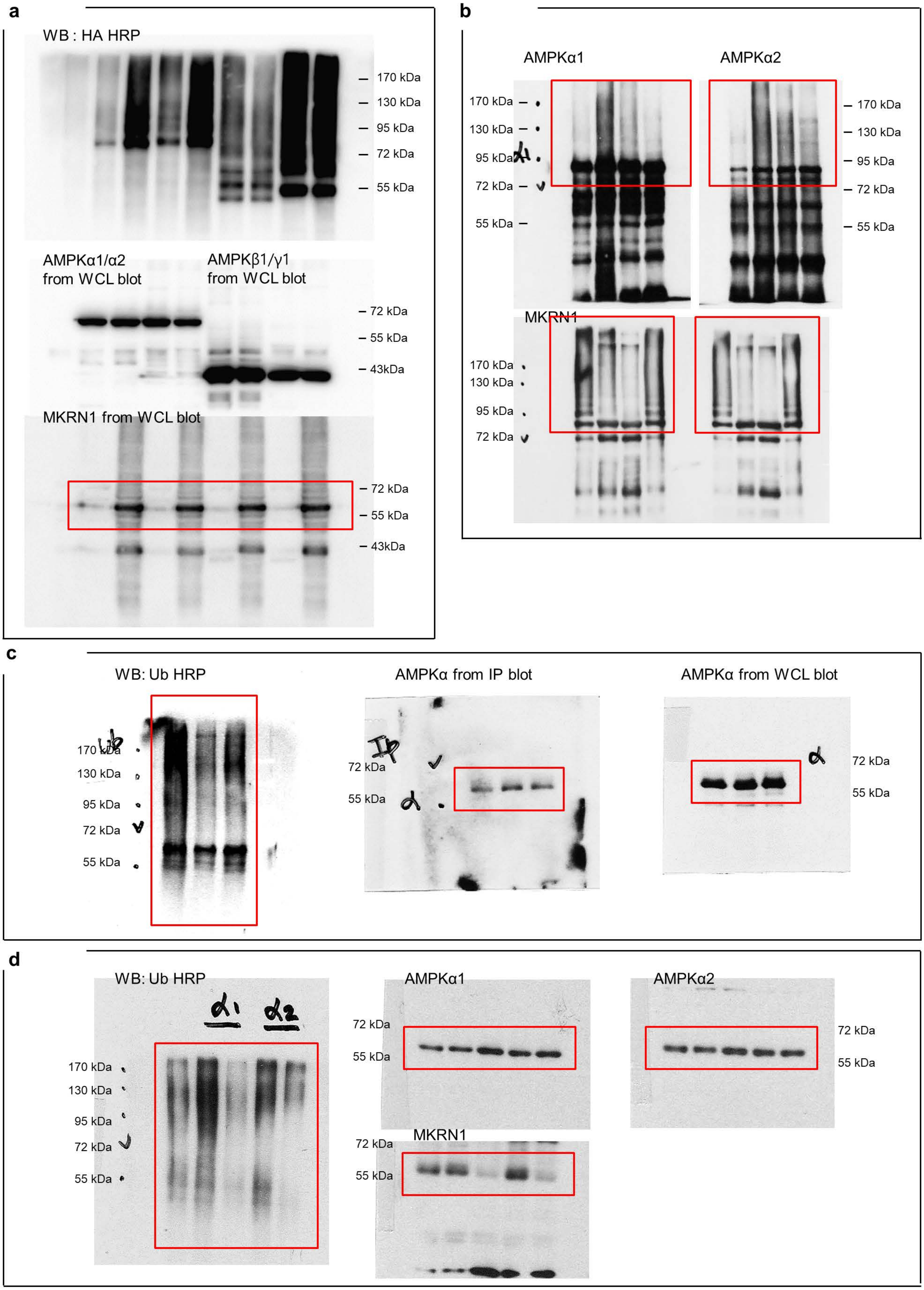

Supplementary Figure 16. Uncropped blots.
(**a-d**) Uncropped Western blots showing molecular weight markers from Figure 2e (**a**), 2f (**b**:left), 2g (**b**:right), 2h (**c**) and 2i (**d**).

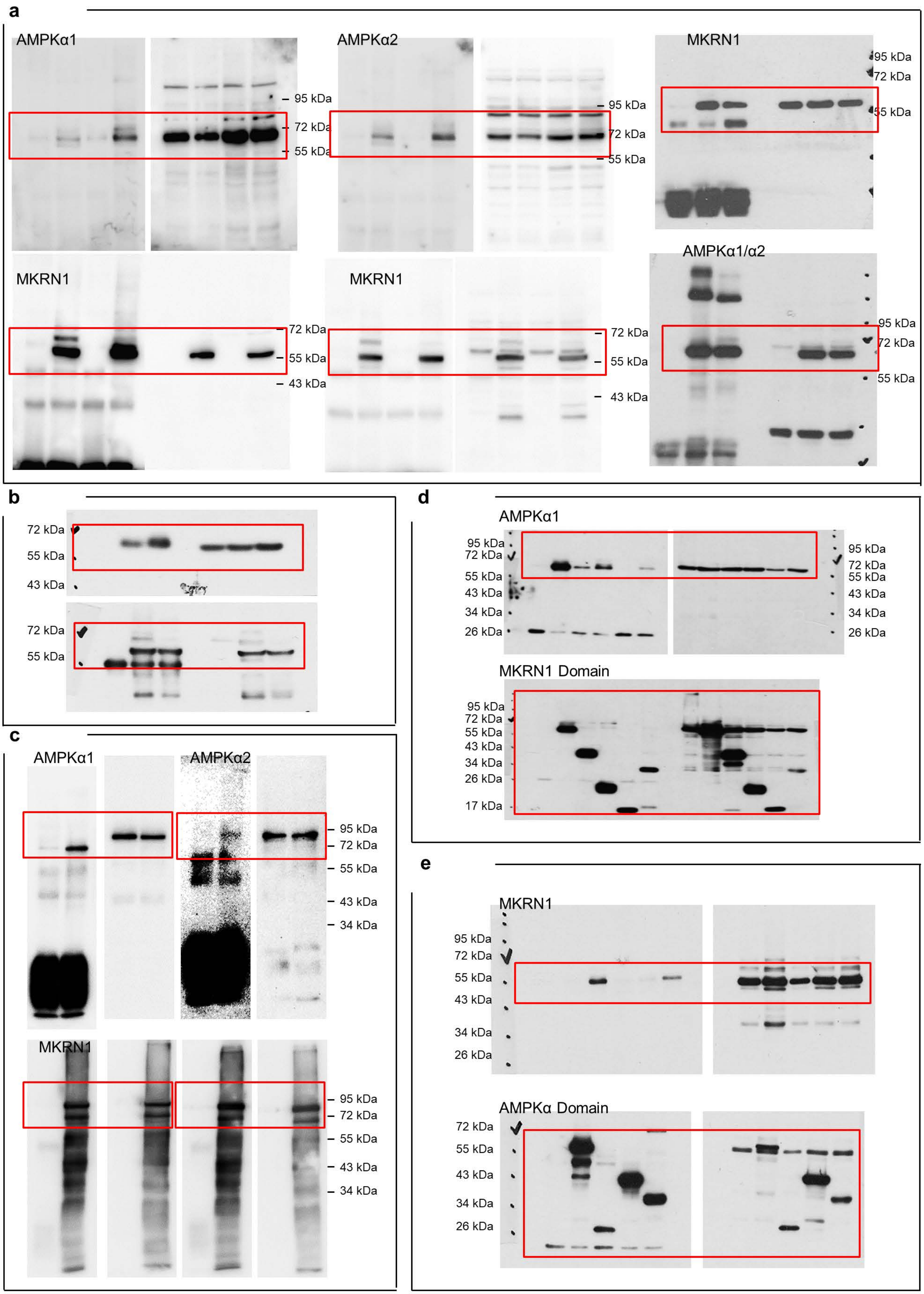

Supplementary Figure 17. Uncropped blots.
(**a-e**) Uncropped Western blots showing molecular weight markers from Supplementary Figure 2a (**a**), 2b (**b**), 2c (**c**), 2d(**d**) and 2e (**e**).

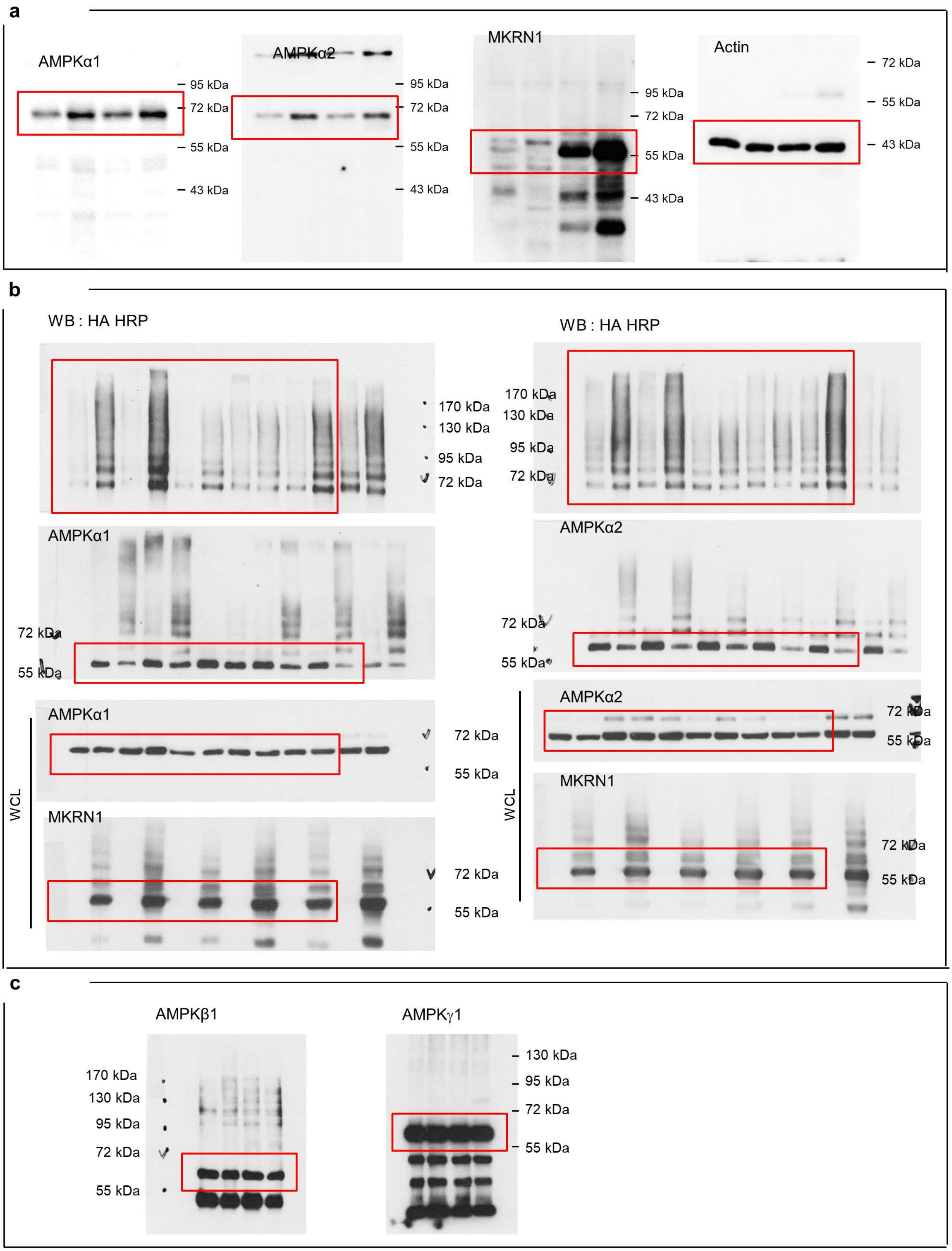

Supplementary Figure 18. Uncropped blots.

(**a-c**) Uncropped Western blots showing molecular weight markers from Supplementary Figure 3a (**a**), 3b (**b**:left), 3c (**b**:right), and 3d (**c**).

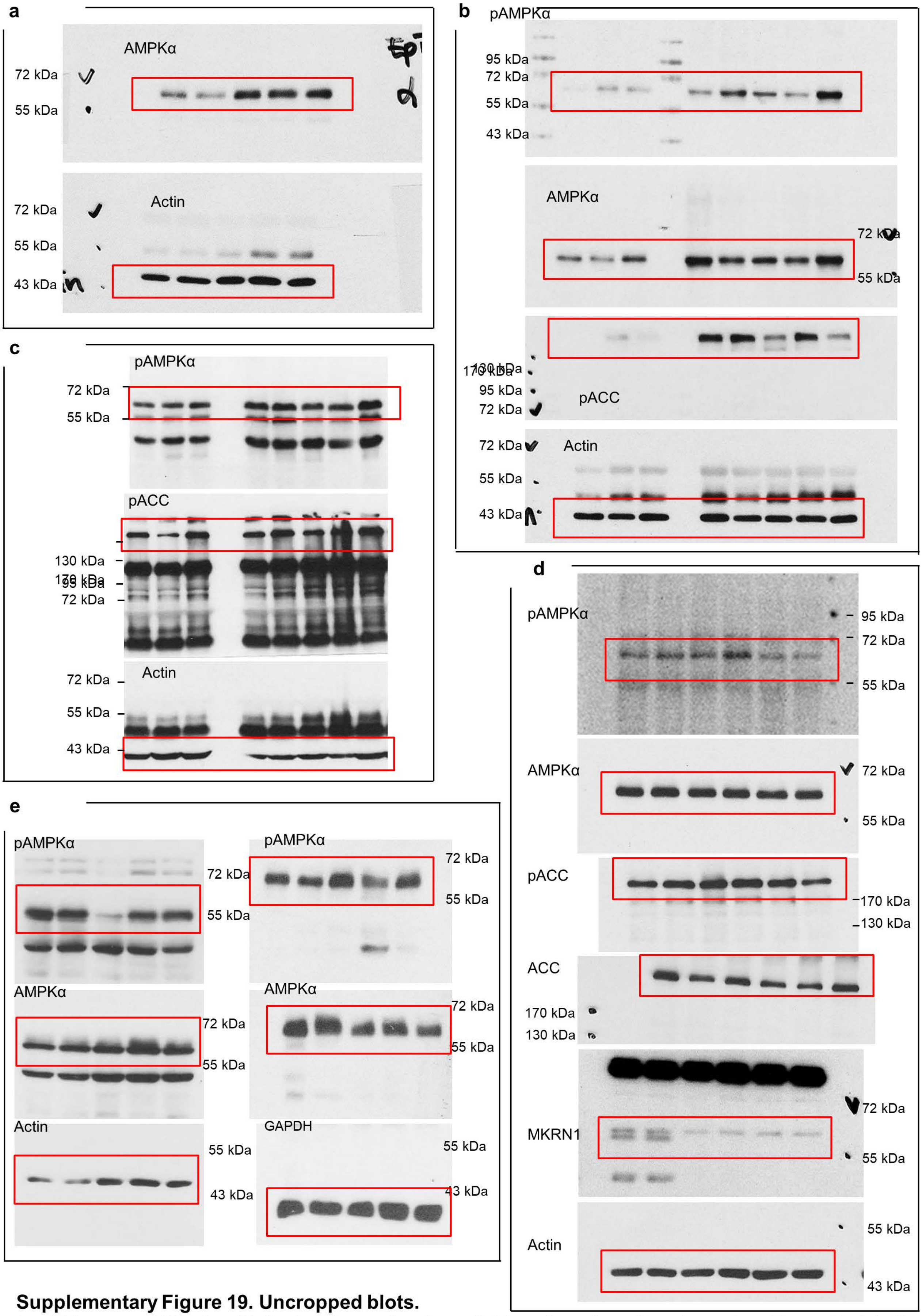

Supplementary Figure 19. Uncropped blots.
(**a-e**) Uncropped Western blots showing molecular weight markers from Supplementary Figure 6a (**a**), 6b (**b**), 6c (**c**), 6d (**d**), 6e (**e**:left) and 6f (**e**:right).

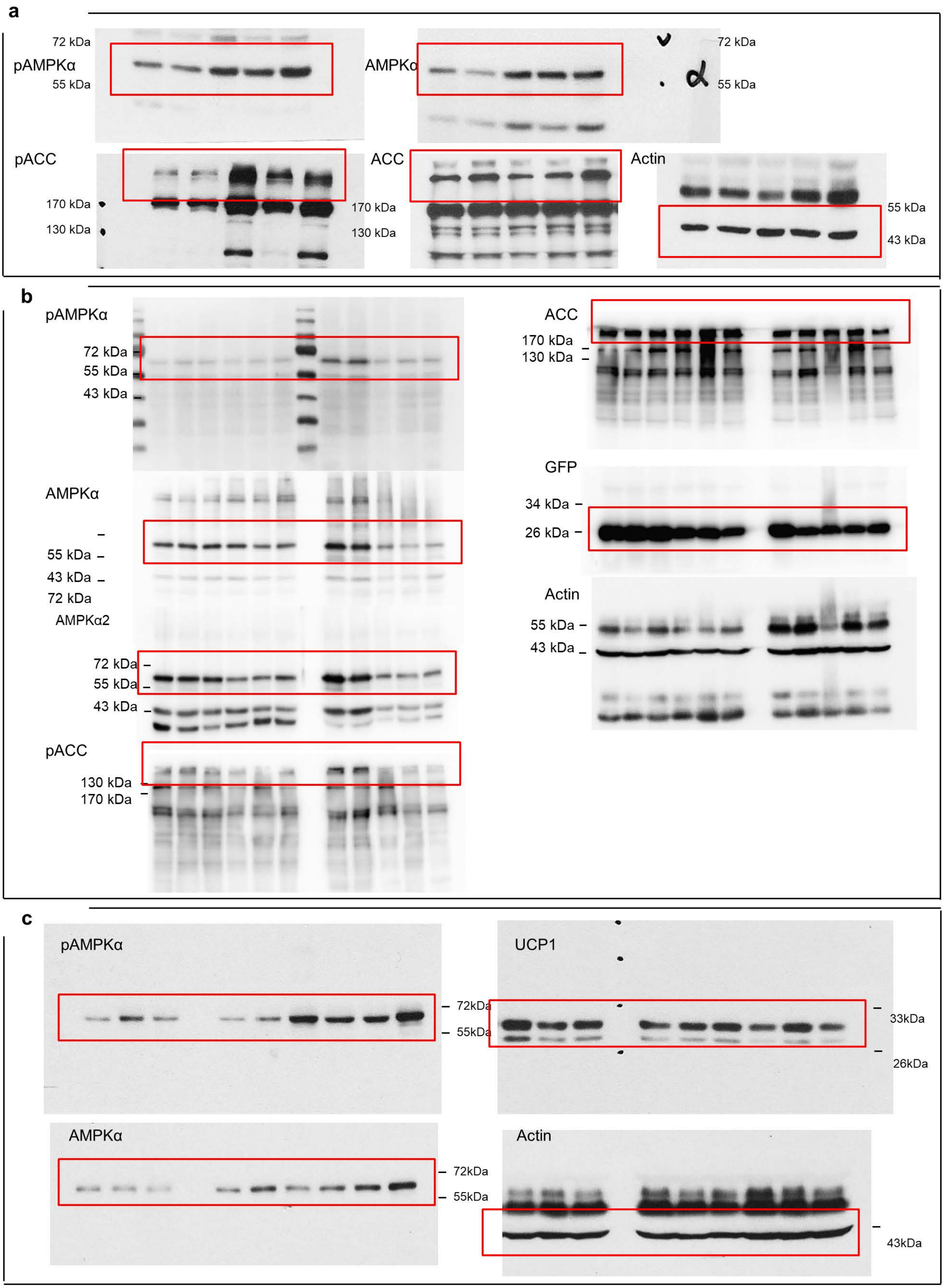

Supplementary Figure 20. Uncropped blots.
(**a-c**) Uncropped Western blots showing molecular weight markers from Figure 4g (**a**), Supplementary figure 8b (**b**) and 10b (**c**).

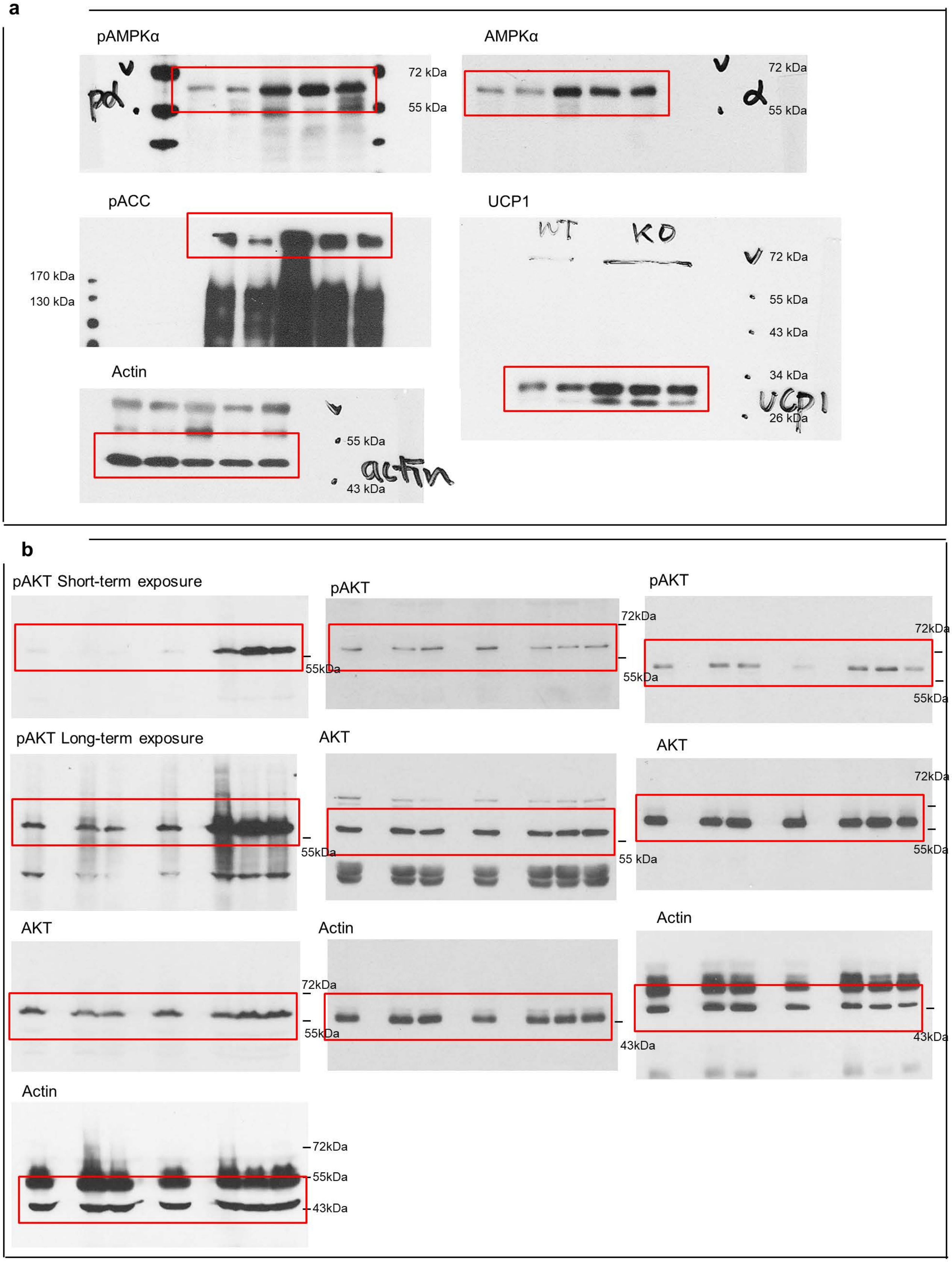

Supplementary Figure 21. Uncropped blots.
(**a-c**) Uncropped Western blots showing molecular weight markers from Supplementary Figure 11c (**a**) and 12e (**b**).

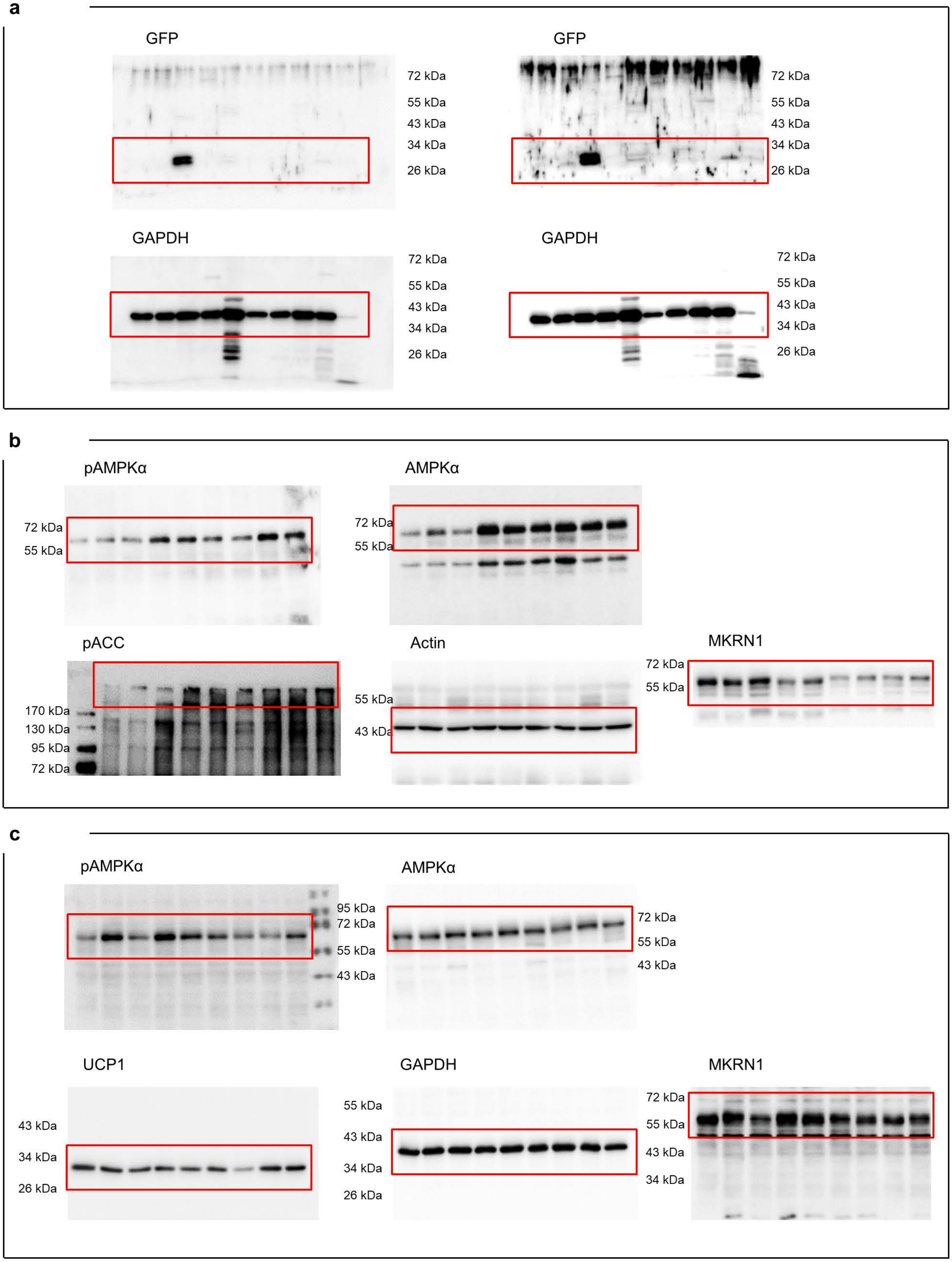

Supplementary Figure 22. Uncropped blots.
(**a-c**) Uncropped Western blots showing molecular weight markers from Figure 6b (**a**), 6c (**b**) and 6d (**c**).

Supplementary Reference

1. Lee, E. W. et al. Differential regulation of p53 and p21 by MKRN1 E3 ligase controls cell cycle arrest and apoptosis. *EMBO J.* **28**, 2100-2113 (2009).
