# Supplementary material for: Loss of the E3 ubiquitin ligase MKRN1 represses diet-induced metabolic syndrome through AMPK activation
Source: Nat Commun. 2018 Aug 24;9:3404. doi: 10.1038/s41467-018-05721-4 (PMC6109074; doi:10.1038/s41467-018-05721-4)
Supplement: Supplementary file 3 — Description of Additional Supplementary Files [file 41467_2018_5721_MOESM3_ESM.docx]

**Description of Additional Supplementary Files**

File Name: Supplementary Data 1

Description: For each DEG, gene symbol and description, P-value, log2-fold-change of mRNA expression levels between MKRN1 KO and WT mice are presented.
