# Supplementary material for: Loss of the E3 ubiquitin ligase MKRN1 represses diet-induced metabolic syndrome through AMPK activation
Source: Nat Commun. 2018 Aug 24;9:3404. doi: 10.1038/s41467-018-05721-4 (PMC6109074; doi:10.1038/s41467-018-05721-4)
Supplement: Supplementary file 2 — Peer Review [file 41467_2018_5721_MOESM2_ESM.pdf]

Reviewers' comments:

Reviewer #1 (Remarks to the Author):

The manuscript "Loss of the E3 ubiquitin ligase MKRN1 represses diet-induced metabolic syndrome though AMPK activation" by Min-Sik Lee et al. describes a mechanism by which AMPK is regulated in a tissue-specific manner. The authors demonstrate in cell culture and in vitro experiments that the ubiquitin ligase MKRN1 binds to AMPK alpha, mediating its ubiquitination and degradation. Knockout of MKRN1 leads to reduced bodyweight gain and increased AMPK activity in liver and fat, and both acute and genetic MKRN1 loss reduces hepatic steatosis upon high fat diet feeding, which is dependent on AMPK. It is overall a good manuscript with sound data, with a few flaws (listed below), that will be of interest to many groups working on AMPK and tissue-specific metabolic regulation.

The increased AMPK activity upon MKRN1 depletion has been shown before by the same authors. The novelty of this manuscript therefore lies in (a) the molecular characterization of the AMPK/MKRN1 interaction and (b) the relevance to metabolic disease. AMPK activity has proven significance for hepatic steatosis, and the manuscript adds a layer of regulation of this factor. The authors claim that it is the first time that AMPK regulation by ubiquitination has been implicated in metabolic disease. However, a study by Qi et al. (EMBO J 2008) demonstrates a role of AMPK ubiquitination for BAT function – this study should also be included in the reference list.

The abstract and main text are clearly written and cohesive. Both the in vitro and the in vivo data are presented clearly and well in the figures and seem of good quality. The approach is largely valid, although in some cases (outlined below) other cell lines or additional in vivo experiments are necessary. Some figure legends lack information, in particular in the supplementary information. Antibodies used for western blots should be specified for every blot. Also, the methodology section is not detailed enough for glucose uptake and immunoprecipitation. Hep3B cells are not mentioned in the methods section.
Throughout the manuscript, t-test has been used wrongly in multiple comparison settings. For example, Fig 1d,e, 3c,h, 4c,i,l, 5e require 2-way ANOVA (2 factorial design); Figure 6f requires 1-way ANOVA. Also, the authors should indicate if they tested for Gaussian distribution. If the samples are not normally distributed the use of non-parametric tests is required instead of t-test.

The data interpretation and drawn conclusions are largely justified with two exceptions: The data on energy expenditure (Fig 5), and the data on glucose uptake and metabolism (Fig 1) as outlined below. In some instances, additional data would be needed to support the claims. Western blot quantification would allow to draw better conclusions regarding protein content in the mouse studies.

The reported energy expenditure data is problematic (Fig 5). Energy expenditure is calculated relative to body weight - which is reduced in MKRN-/- mice. Naturally, dividing by a smaller number yields a larger result. However, based on the consensus reporting for energy expenditure data (Tschop et al. Nat Med 2012), energy expenditure needs to be calculated using ANCOVA, since body weight and energy expenditure do not display a linear relationship. A critical view on the data suggests minimal changes to energy expenditure once body weight differences are accounted for, making assumptions based on this data difficult. Thus, the section on BAT and energy expenditure needs to be thoroughly revised.

Additional suggested improvements:

- Fig 1a/b: It is not described clearly how glucose uptake was measured for Fig. 1. The authors describe glucose measurement from medium, which is not an accurate measurement of glucose uptake. Traced glucose should be used instead to accurately measure glucose uptake. There is a discrepancy between glucose uptake (5-fold) and levels of glycolytic intermediates (1.2 to 1.5-

fold). To draw any meaningful conclusions, the flux through the pathway needs to be quantified using labelled substrate.

- Figure 1 seems somewhat disconnected from the rest of the story. The relevance needs to be better addressed. The authors show increased glucose uptake upon MKRN1 kd in cells. Is this also observed in liver and AT? If so, how does this relate to decreased lipogenesis? Is liver glycogen or lactate increased? Are genes involved in glucose uptake and glycolysis also altered in liver?

- Regarding Co-immunoprecipitation (Sup Fig 2): The method and system used are not described accurately. If, as the authors claim and also show in Fig 2c, MKRN1 ubiquitinates AMPK, leading to its degradation, then AMPK should be degraded by MKRN1 over expression. Were proteasome inhibitors added during culture? Was a cell-free system used? How is this lack of effect explained?

- The rescue of AMPK degradation by proteasomal inhibition (MG132) is not adequately shown. For one, rescue of AMPK degradation MG132 (Fig. 2d) should be shown in a relevant cell type instead of HEK cells. Second, Fig. 2e, Sup3a,b show reduced AMPKa levels in response to MKRN1 expression despite presence of MG132.

- H307E is an enzymatically inactive MKRN1 mutant which the authors show in the figures (2f,g and supplementary Fig). This is not mentioned in the text. Also, to demonstrate specificity of the enzymatic activity more experiments are needed (e.g. AMPK levels upon re-expression of mutant vs control MKRN1 in MKRN-/-MEFS)

- Strong conclusions are drawn from the absence of AMPK activation in the hypothalamus upon MKRN1 depletion (Sup Fig.6d, lines 189-195). However, pACC levels appear elevated in MKRN1-/- hypothalamus. To draw strong conclusions, this effect should be quantified and undergo statistical testing.

- Using AMPK knockdown in livers of MKRN1 knockout mice, the authors show the dependence of the liver phenotype on AMPK (Fig. 4j-l). However, the rescue experiment needs more experimental readout to support the rescue effect, such as gene expression of lipogenic genes and SREBP1/ChREBP

- AMPK ubiquitination in dependence of MKRN1 should be shown in vivo to support the claims, since all ubiquitination and interaction studies were performed in tissue culture.

Discussion:
- One of the main targets of AMPK in the liver is mTOR. Is mTOR protein and pathway activity affected by MKRN knockout? Or is there a discrepancy between different AMPK pathways? If so, how is this explained?

- One other target of MKRN1 is PPARgamma (Kim et al. Cell Death and Differentiation 2014). In the liver, PPARalpha might be a target– which is a major regulator of lipid metabolism. This needs to be discussed

Minor comments
- Line 170: what is meant by “the different chow feeding conditions”? Only one chow feeding condition is explained. Please specify
- The MKRN1 antibody used in this study (A300-990A; Bethyl Laboratories) is immunogen between 432 and C-terminus. Please indicate which antibodies were used for immunoblot in Sup Fig 2d,e (HA or MKRN1)

Reviewer #2 (Remarks to the Author):

This study assesses the metabolic role of MKRN1 in vivo. The results are novel and of interest, with a very clear phenotype in mice lacking this gene. More specifically, these mice are protected against diet induced obesity and NAFLD. These alterations are mediated by AMPK activation. Since the model used here is a global knockout of MKRN1, the doubt is where this gene acts primarily to protect mice against high fat diet. The fact that AMPK is not activated in the hypothalamus restrings the possibilities and the focus in BAT and liver is a logical approach to answer this question. However, additional studies must be performed to clearly address some issues:

1. MKRN1-deficient mice show a remarkable lean phenotype when fed a high fat diet. The explanation for this lean phenotype is the increased energy expenditure. Since the authors have performed indirect calorimetry measurements, it would be also interesting to see data on respiratory exchange ratio, as this is an indirect marker for lipid utilization and there are several figures of the study indicating that the lack of MKRN1 alters lipid metabolism. In line with this, it would be also interesting to address whether there are alterations in lipid absorption that might potentially contribute to the DIO protection.

2. The protection of MKRN1-deficient mice against diet-induced NAFLD is also clear. The authors have just measured some genes involved in fatty acid synthesis (Srebp-1 and ChREBP), but it is well established that AMPK regulates also lipolysis. In order to clarify how MKRN1/AMPK affect lipid deposition in the liver, a more comprehensive investigation should be made, and assessing fatty acid synthesis and oxidation per se would be much precise than just measuring a few markers.

3. MKRN1 deficiency led to long-term AMPK activation in BAT and WAT. Figure 5 focuses in BAT and energy expenditure, but surprisingly there are no data on WAT. Recent papers have established that the lack of AMPK in white adipose tissue causes important metabolic alterations, and therefore, it is logical to hypothesize that the activation of WAT AMPK might contribute to the phenotype of MKRN1-deficient mice. A detailed study of WAT should be performed here (histology, lipogenesis, lipolysis, browning, etc)

4. The final set of data is also very interesting as they highlight the relevance of MKRN1 in the liver. The findings obtained in the liver of leaner MKRN1 ko mice might be simply the result of the lower amount of global fat in this model, but the results of figure 6 point to a direct effect of MKRN1 in the liver. In addition to these hepatic effects, is there any change in body weight or adiposity after the acute knockdown of MKRN1 in the liver? This might help to understand if the liver contributes to the leaner phenotype of MKRN1 ko mice when fed a HFD.

Minor:
Page 13, line 178. Do the authors mean energy expenditure instead energy intake? It was stated a few lines above that food intake remained unchanged.

Reviewer #3 (Remarks to the Author):

This is an interesting paper which has identified a novel mechanism regulating AMPK expression in brown adipose tissue and the liver. The findings are interesting and largely consistent with the role of AMPK in regulating metabolism in these tissues.

Major Comments:
1. The increase in glycolysis in the MEFS is quite surprising given the activation of AMPK which would be expected to counter this response by promoting oxidative metabolism and mitochondrial biogenesis. Did MEFS have increases in mitochondrial biogenesis, fatty acid oxidation and reductions in lipogenesis as one would anticipate given the increase in ACC phosphorylation and if so why was glycolysis increased under these conditions.

2. In order to understand the physiological significance of the findings it would be interesting to know whether the expression of the ubiquitin ligase MKRN1 is elevated with obesity and diabetes.
3. Figure 6 Authors have found that liver lipids are lower in mice treated with the liver directed shRNA to MKRN1. Body mass and adiposity of these mice should be reported. Similarly it would be important to measure liver lipogenesis under these conditions as previous studies have indicated this is the primary mechanism by which increases in AMPK reduces liver lipid content (Fullerton et al. Nat Med 2013).

Minor Comments:
1. The manuscript is full of grammatical errors that must be corrected.
2. There is a large number of reviews cited some of which are quite dated. It would be best for authors to use primary research articles to support claims and more recent reviews in select cases.

Reviewers' comments:

Reviewer #1 (Remarks to the Author):

The manuscript "Loss of the E3 ubiquitin ligase MKRN1 represses diet-induced metabolic syndrome though AMPK activation" by Min-Sik Lee et al. describes a mechanism by which AMPK is regulated in a tissue-specific manner. The authors demonstrate in cell culture and in vitro experiments that the ubiquitin ligase MKRN1 binds to AMPK alpha, mediating its ubiquitination and degradation. Knockout of MKRN1 leads to reduced bodyweight gain and increased AMPK activity in liver and fat, and both acute and genetic MKRN1 loss reduces hepatic steatosis upon high fat diet feeding, which is dependent on AMPK. It is overall a good manuscript with sound data, with a few flaws (listed below), that will be of interest to many groups working on AMPK and tissue-specific metabolic regulation.

1. The increased AMPK activity upon MKRN1 depletion has been shown before by the same authors. The novelty of this manuscript therefore lies in (a) the molecular characterization of the AMPK/MKRN1 interaction and (b) the relevance to metabolic disease. AMPK activity has proven significance for hepatic steatosis, and the manuscript adds a layer of regulation of this factor. The authors claim that it is the first time that AMPK regulation by ubiquitination has been implicated in metabolic disease. However, a study by Qi et al. (EMBO J 2008) demonstrates a role of AMPK ubiquitination for BAT function – this study should also be included in the reference list.

Answer:

It is noteworthy (as the Reviewer pointed out) that a link between AMPK ubiquitination and energy metabolism has been previously recognized[1]. Whether an E3 ubiquitin ligase can control systemic metabolism through AMPK regulation has yet to be more comprehensively addressed. We have added the Qi *et al*. reference in the main text and have corrected the paragraph claiming that MKRN1 plays a novel role as an E3 ligase for AMPK: see main text, lines 92-95 of the manuscript, on page 5.

2. The abstract and main text are clearly written and cohesive. Both the in vitro and the in vivo data are presented clearly and well in the figures and seem of good quality. The approach is largely valid, although in some cases (outlined below) other cell lines or additional in vivo experiments are necessary. Some figure legends lack information, in particular in the supplementary information. Antibodies used for western blots should be specified for every blot. Also, the methodology section is not detailed enough for glucose uptake and immunoprecipitation. Hep3B cells are not mentioned in the methods section.

Answer:

As the Reviewer suggested, we have augmented the Supplementary Information and legends throughout the manuscript. In particular, we have added detailed information on the glucose uptake assay, immunoprecipitation methodology, and Hep3B cell line in the Methods section of the revised manuscript and have indicated the antibodies used for western blotting in the figure legends. Additionally, we have provided more complete information for the supplementary figures.

3. Throughout the manuscript, t-test has been used wrongly in multiple comparison settings. For example, Fig 1d,e, 3c,h, 4c,i,l, 5e require 2-way ANOVA (2 factorial design); Figure 6f requires 1-way ANOVA. Also, the authors should indicate if they tested for Gaussian distribution. If the samples are not normally distributed the use of non-parametric tests is required instead of t-test.

Answer:

We apologize for not clarifying this better in the first submission. As suggested by the Reviewer, we performed 2-way ANOVA for the data in Fig. 1d,e, Fig. 3c,h, Fig. 4c,i,l, and Fig. 5e (these data are newly presented in Supplementary Fig. 1d , Fig. 3c,h, Fig. 4c,i,l and Fig. 11e-g.) and 1-way ANOVA for Fig. 6g. The normality of the distribution was assessed with the Kolmogorov–Smirnov method. Statistical significance between the 2 groups was determined using 2-tailed Student's *t* tests for normally distributed data and Mann-Whitney U tests for nonparametric analyses.
These analyses have been clarified in the Methods section of the revised manuscript.

4. The data interpretation and drawn conclusions are largely justified with two exceptions: The data on energy expenditure (Fig 5), and the data on glucose uptake and metabolism (Fig 1) as outlined below. In some instances, additional data would be needed to support the claims. Western blot quantification would allow to draw better conclusions regarding protein content in the mouse studies.

The reported energy expenditure data is problematic (Fig 5). Energy expenditure is calculated relative to body weight - which is reduced in MKRN-/- mice. Naturally, dividing by a smaller number yields a larger result. However, based on the consensus reporting for energy expenditure data (Tschop et al. Nat Med 2012), energy expenditure needs to be calculated using ANCOVA, since body weight and energy expenditure do not display a linear relationship. A critical view on the data suggests minimal changes to energy expenditure once body weight differences are accounted for, making assumptions based on this data difficult. Thus, the section on BAT and energy expenditure needs to be thoroughly revised.

Answer:

As the reviewer correctly noted, there was no difference in energy expenditure between the wild-type and knockout mice according to ANCOVA. Thus, we removed our interpretation regarding energy expenditure in BAT, as suggested. The data are newly presented in Supplementary Fig.11g, and the description is included in line 320 of the manuscript, on page 14.

Additional suggested improvements:

5. - Fig 1a/b: It is not described clearly how glucose uptake was measured for Fig. 1. The authors describe glucose measurement from medium, which is not an accurate measurement of glucose uptake. Traced glucose should be used instead to accurately measure glucose uptake. There is a discrepancy between glucose uptake (5-fold) and levels of glycolytic intermediates (1.2 to 1.5-fold). To draw any meaningful conclusions, the flux through the pathway needs to be quantified using labelled substrate.

Answer:

We measured glucose uptake by quantifying the uptake of 2-[N-(7-nitrobenz-2-oxa-1,3-diazol-4-yl) amino]-2-deoxy-D-glucose (2-NBDG), which was measured based on fluorescence using flow cytometry. All the data are newly presented in Fig. 1a (in line 115 of the manuscript, on page 6) and Supplementary Fig. 1f (in line 133 of the manuscript, on page 7).

6. - Figure 1 seems somewhat disconnected from the rest of the story. The relevance needs to be better addressed. The authors show increased glucose uptake upon MKRN1 kd in cells. Is this also observed in liver and AT? If so, how does this relate to decreased lipogenesis? Is liver glycogen or lactate increased? Are genes involved in glucose uptake and glycolysis also altered in liver?

Answer:

This is a valid question that needs to be addressed. Unfortunately, neither our facilities nor those of our collaborators have the appropriate equipment to measure the direct uptake of glucose in the liver and AT of mice. The facility in Korea that has these systems requires embryonic transfer to obtain knockout mice, and the limited allotted time for revisions does not allow us to perform these in vivo studies.

Alternatively, to thoroughly observe the systemic changes in knockout and wild-type mice on a high-fat diet, we performed RNA-Seq analyses. Network analysis showed differential regulation of glucose and fatty acid metabolism. In the liver, the genes involved in glycolysis (Hk1/2, Gpi1, Pfkp, Aldoa, Gapdh, Eno1, Pkm and Pklm) and gluconeogenesis (G6pc and Pck2) were down-regulated (see revised Fig. 5e above). In adipose tissue, however, the genes involved in glucose uptake (Slc2a4/Glu4) and glycolysis were up-regulated as were those involved in lipolysis (see revised Fig. 5f above). On the other hand, lactate dehydrogenase was up-regulated in adipose tissue (Ldha), but down-regulated (Ldhb) in the liver. This up- and down-regulation of glucose utilization in adipose tissue and the liver, respectively, reflects a greater supply of acetyl-CoA for fatty acid biosynthesis in adipose tissue than in the liver. These data suggest systematic differential regulation of fatty acid and glucose metabolism in the liver and adipose tissue by MKRN1 and AMPK (Fig. 5: The data are described in line 254 of the manuscript, on page 12).

Our MEF data are very similar to the gene expression profile of adipose tissue. Glucose transporters and the glycolysis pathway, lactate and acetyl CoA production, the TCA cycle and oxidative phosphorylation were all increased in the adipose tissue of MKRN1 KO mice and in MEFs depleted of MKRN1. Furthermore, we were able to observe that fatty acid oxidation was increased in MKRN1-null MEFs (Fig. 1k) as well as MKRN1-KO adipose tissue. Our data are consistent with a previous report demonstrating a preference for AMPK activation to take up glucose and use it as an energy source[2-5].

There was no obvious difference in the levels of glycogen in the MKRN1-null or wild-type mice with or without HFD feeding (Supplementary Fig. 7a: The data are described in line 235 of the manuscript, on page 11). The metabolic pathway related to lipid metabolism seems to be more significantly affected in MKRN1-null mice.

The RNA-Seq analyses are explained in the Results and Discussion sections (in line 394 of the manuscript, on page 17).

7. - Regarding Co-immunoprecipitation (Sup Fig 2): The method and system used are not described accurately. If, as the authors claim and also show in Fig 2c, MKRN1 ubiquitinates AMPK, leading to its degradation, then AMPK should be degraded by MKRN1 over expression. Were proteasome inhibitors added during culture? Was a cell-free system used? How is this lack of effect explained?

Answer:

We confirmed the interaction between AMPK and MKRN1 with or without treatment with MG132, a proteasome inhibitor. Regardless of MG132 treatment, the two proteins interacted. The data are presented in Supplementary Fig. 2a, and more detailed explanations of the methods and results are included in line 153 of the manuscript, on page 8.

8. - The rescue of AMPK degradation by proteasomal inhibition (MG132) is not adequately shown. For one, rescue of AMPK degradation MG132 (Fig. 2d) should be shown in a relevant cell type instead of HEK cells. Second, Fig. 2e, Sup3a,b show reduced AMPKa levels in response to MKRN1 expression despite presence of MG132.

Answer:

The indicated experiments were repeated with better results, which are presented (Fig. 2d). The HepG2 cell line was employed instead of HEK cell line in these experiments.

In Supplementary Fig. 3b and 3c (re-labelled from Supplementary Fig. 3a and 3b), the levels of AMPK in whole-cell lysates (WCL) presented in the lower panels of the figure were not changed by MKRN1 overexpression upon MG123 treatment.

9. - H307E is an enzymatically inactive MKRN1 mutant which the authors show in the figures (2f,g and supplementary Fig). This is not mentioned in the text. Also, to demonstrate specificity of the enzymatic activity more experiments are needed (e.g. AMPK levels upon re-expression of mutant vs control MKRN1 in MKRN-/- MEFS)

Answer:

We mentioned that H307E is an enzymatically inactive form of MKRN1 in the line 160 of the manuscript, on page 8.
As suggested, rescue analyses were performed by expressing siRNA-resistant MKRN1 (Supplementary Fig. 3a). We were able to observe that the accumulated AMPK alpha induced by ablation of MKRN1 was degraded by overexpression of siRNA-resistant MKRN1. On the other hand, the MKRN1 H307E mutant was not able to induce the degradation of AMPK alpha. These data are included in Supplementary Fig. 3a, and the corresponding description is included in line 160 of the manuscript, on page 8.

10. - Strong conclusions are drawn from the absence of AMPK activation in the hypothalamus upon MKRN1 depletion (Sup Fig.6d, lines 189-195). However, pACC levels appear elevated in MKRN1-/- hypothalamus. To draw strong conclusions, this effect should be quantified and undergo statistical testing.

Answer:

When the levels of pACC were quantified, no difference was found between WT and KO mice (Supplementary Fig. 6d). The data are presented in Supplementary Fig. 6d, and the description is included in line 206 of the manuscript, on page 10.

11. - Using AMPK knockdown in livers of MKRN1 knockout mice, the authors show the dependence of the liver phenotype on AMPK (Fig. 4j-l). However, the rescue experiment needs more experimental readout to support the rescue effect, such gene expression of lipogenic genes and SREBP1/ChREBP

Answer:

As suggested, we analysed the mRNA levels of SREBP-1 and ChREBP in the liver upon injection of sh-MKRN1 adenovirus via qRT analysis, (Fig. 4m). These data corroborated the findings from the immunohistochemistry data. The explanation of these data is presented in the Results section in line 248 of the manuscript, on page 11

12. - AMPK ubiquitination in dependence of MKRN1 should be shown in vivo to support the claims, since all ubiquitination and interaction studies were performed in tissue culture.

Answer:

As the reviewer correctly suggested, we tried to observe the ubiquitinated forms of AMPK in both KO and WT several times with no success. To our frustration, we could not overcome the technical difficulties involved in these experiments. The antibodies for ub and AMPK might not be sufficiently sensitive to detect ubiquitinated forms of AMPK. Alternatively, detection of the ubiquitinated forms of endogenous AMPK in the cells requires several hours of MG132 treatment, as seen in Fig. 2h and i. In the ubiquitination analyses of the liver, this step was not possible, making the detection of accumulated forms of ubiquitinated AMPK very difficult. We hope that the reviewer understands the difficulty of detecting endogenous forms of ubiquitinated proteins from organs.

Discussion:
13. - One of the main targets of AMPK in the liver is mTOR. Is mTOR protein and pathway activity affected by MKRN knockout? Or is there a discrepancy between different AMPK pathways? If so, how is this explained?

Answer:

According to gene expression analysis, the mRNA expression levels of genes in the mTOR signalling pathway were down-regulated: mTOR and its upstream (Hras, Mapk3, Pik3r1 and Rheb) and downstream (Eif4e, Sgk1, Pparg and Srebf1) genes were down-regulated in the liver, while its upstream (Pik3r1/3, Pik3cb and Akt2) and downstream (Rps6kb2, Eif4e, Prkca/g and Pparg) genes were down-regulated in adipose tissue (see revised Supplementary Fig. 9a,b below). These data suggest that activation of AMPK via MKRN1 ablation might have a strong negative effect on mTOR signalling in the liver and adipose tissue. To clarify this point, we have added a paragraph to the Results and Supplementary Fig. 9a,b.

Based on these analyses, it is difficult to accurately interpret whether there is any discrepancy between different AMPK pathways. Notably, Prkaa2/AMPKα2, Prkab2/AMPKβ2, and Prkag3/AMPKγ3 were up-regulated in adipose tissue by MKRN1 ablation but showed no expression changes in the liver, suggesting that these AMPKs were up-regulated at the mRNA level in adipose tissue to accommodate metabolic needs arising from increased glucose utilization (see Fig. 5 above and our response to Comment 3 below).

These results are addressed in the Results and Discussion sections (in line 394 of the manuscript, on page 17).

14. - One other target of MKRN1 is PPARgamma (Kim et al. Cell Death and Differentiation 2014). In the liver, PPARalpha might be a target– which is a major regulator of lipid metabolism. This needs to be discussed.

Answer:

We did indeed detect PPARγ and α in both the liver and AT using RNA-seq. The levels of PPARγ were suppressed in the adipose tissue of MKRN1 KO mice, possibly due to their small amount of fat tissue compared with that in the wild-type. There was no difference in the mRNA levels of PPARα in the liver. We do not exclude the possibility that MKRN1 might target PPARα at the post-translational level. This possibility is discussed in line 405, on page 18.

Minor comments

- Line 170: what is meant by "the different chow feeding conditions"? Only one chow feeding condition is explained. Please specify

Answer:

We apologize for the confusion caused by this sentence. We corrected the sentence as follows:
No differences in body weight were observed under the standard chow feeding conditions. (original sentence: No differences in body weight were observed under the different chow feeding conditions)

- The MKRN1 antibody used in this study (A300-990A; Bethyl Laboratories) is immunogen between 432 and C-terminus. Please indicate which antibodies were used for immunoblot in Sup Fig 2d,e (HA or MKRN1)

Answer:

We detected MKRN1using an antibody for HA. We have re-labelled the figure.

Reviewer #2 (Remarks to the Author):

This study assesses the metabolic role of MKRN1 in vivo. The results are novel and of interest, with a very clear phenotype in mice lacking this gene. More specifically, these mice are protected against diet induced obesity and NAFLD. These alterations are mediated by AMPK activation. Since the model used here is a global knockout of MKRN1, the doubt is where this gene acts primarily to protect mice against high fat diet. The fact that AMPK is not activated in the hypothalamus restrings the possibilities and the focus in BAT and liver is a logical approach to answer this question. However, additional studies must be performed to clearly address some issues:

1. MKRN1-deficient mice show a remarkable lean phenotype when fed a high fat diet. The explanation for this lean phenotype is the increased energy expenditure. Since the authors have performed indirect calorimetry measurements, it would be also interesting to see data on respiratory exchange ratio, as this is an indirect marker for lipid utilization and there are several figures of the study indicating that the lack of MKRN1 alters lipid metabolism. In line with this, it would be also interesting to address whether there are alterations in lipid absorption that might potentially contribute to the DIO protection.

Answer:

As the reviewer commented, we compared the respiratory exchange ratio (RER) between the WT and MKRN1 KO groups. The RER phase of WT and KO was between 7.5 and 7.0, indicating that fat is the major energy source for both groups of mice. These data are presented in Supplementary Fig. 11.f, and the description is provided in line 319 of the manuscript, on page 14.

We also measured the TG and FFA contents of the faeces of WT or KO mice fed with the HFD, in addition to faecal gross calories. As shown in Supplementary Fig. 4d, there was no difference between WT and KO, indicating that the lean phenotype could be based on metabolic consumption (line 191 of the manuscript, on page 9).

2. The protection of MKRN1-deficient mice against diet-induced NAFLD is also clear. The authors have just measured some genes involved in fatty acid synthesis (Srebp-1 and ChREBP), but it is well established that AMPK regulates also lipolysis. In order to clarify how MKRN1/AMPK affect lipid deposition in the liver, a more comprehensive investigation should be made, and assessing fatty acid synthesis and oxidation per se would be much precise than just measuring a few markers.

Answer:

This suggestion is valid, and we therefore analysed the liver and adipose tissue of KO and WT mice fed with the HFD through RNA-seq analyses. The detailed data and explanation are presented in Fig. 5 and the Results section (line 254 of the manuscript, on page 12). Briefly, the patterns of gene expression in both the liver and adipose tissues displayed an increase of lipid energy expenditure and suppression of lipogenesis in KO mice, which corroborates the observed phenotype. The mTOR pathway was suppressed in both tissues of KO mice. Gluconeogenesis was also suppressed in the liver of KO mice. It is important to note that thermogenesis and browning markers were increased in MKRN1 KO mice, corroborating the finding that utilizing lipids for energy consumption is not only preferred but also up-regulated in the KO mice. The detailed data and interpretation are included in Fig. 5, Supplementary Fig. 11 and the Results section (in line 326, on page 15).

3. MKRN1 deficiency led to long-term AMPK activation in BAT and WAT. Figure 5 focuses in BAT and energy expenditure, but surprisingly there are no data on WAT. Recent papers have established that the lack of AMPK in white adipose tissue causes important metabolic alterations, and therefore, it is logical to hypothesize that the activation of WAT AMPK might contribute to the phenotype of MKRN1-deficient mice. A detailed study of WAT should be performed here (histology, lipogenesis, lipolysis, browning, etc)

Answer:

As the reviewer suggested, we performed RNA-seq analyses of KO and WT mice and presented the results in Fig. 5. The data include the results of comprehensive analyses of the metabolic pathways such as lipogenesis, lipolysis and browning in adipose tissues as well as in the liver. The detailed analyses are presented in the Results section beginning on page 12. We have also newly presented histology data for subcutaneous fat in Fig. 3f and 3g, in addition to epididymal fat tissues (in line 197 on page 9). Furthermore, we have presented new mouse phenotype data used for RNA-seq analyses in the Supplementary Fig. 4a.

4. The final set of data is also very interesting as they highlight the relevance of MKRN1 in the liver. The findings obtained in the liver of leaner MKRN1 ko mice might be simply the result of the lower amount of global fat in this model, but the results of figure 6 point to a direct effect of MKRN1 in the liver. In addition to these hepatic effects, is there any change in body weight or adiposity after the acute knockdown of MKRN1 in the liver? This might help to understand if the liver contributes to the leaner phenotype of MKRN1 ko mice when fed a HFD.

Answer:

This is a very interesting point, which might indicate the possibility of employing MKRN1 as a therapeutic target. We attempted these experiments again and found no change in body weight or adiposity after acute injection of sh-MKRN1 adenovirus. This finding might indicate, as the reviewer suggested, that the liver contributes to the leaner phenotype of MKRN1 KO mice when fed an HFD. Currently, we are generating a conditional MKRN1 KO, and more detailed information about the long-term effects of MKRN1 KO in specific organs might therefore be obtained in the future. All the data are presented in Fig. 6e, 6i and 6j (in line 353, on page 16).

Minor:
Page 13, line 178. Do the authors mean energy expenditure instead energy intake? It was stated a few lines above that food intake remained unchanged.

Answer:

We revised the indicated sentences as follows:

Little difference was observed between MKRN1-null and WT mice in plasma leptin levels, supporting the finding that there was no difference in the amount of feeding. In addition, there were no significant changes in gross faecal energy, the levels of TG and free fatty acid in faeces or bone length, suggesting that the decreased body weights of the MKRN1-null mice were not due to changes in nutritional excretion or morphology.

(original section: Little difference was observed in plasma leptin levels or the ability to absorb calories, as indicated by gross faecal energy and bone length, between the two groups fed an HFD, suggesting that the decreased body weights in MKRN1-null mice were not due to changes in energy intake or morphology.)

Reviewer #3 (Remarks to the Author):

This is an interesting paper which has identified a novel mechanism regulating AMPK expression in brown adipose tissue and the liver. The findings are interesting and largely consistent with the role of AMPK in regulating metabolism in these tissues.

Major Comments:
1. The increase in glycolysis in the MEFS is quite surprising given the activation of AMPK which would be expected to counter this response by promoting oxidative metabolism and mitochondrial biogenesis. Did MEFS have increases in mitochondrial biogenesis, fatty acid oxidation and reductions in lipogenesis as one would anticipate given the increase in ACC phosphorylation and if so why was glycolysis increased under these conditions.

Answer:

This is a valid question. The metabolomic analyses showed increased levels of metabolites in the TCA cycle (Fig. 1c, in line 118, on page 6). We also further confirmed the glucose uptake of HepG2 cells and MEFs using 2-NBDG (2-[N-(7-nitrobenz-2-oxa-1,3-diazol-4-yl) amino]-2-deoxy-D-glucose), followed by detection by flow cytometry (Fig. 1a, in line 114, on page 6). There was an increase of glucose uptake upon MKRN KO and knockdown, which was AMPK dependent, suggesting the possibility of an increase in glycolysis (Supplementary Fig. 1d and 1f, in line 133, on page 7). These observations are consistent with the previous reports showing increases of glucose uptake and the glycolysis pathway upon the activation of AMPK[2-5].

Furthermore, there was an increase in fatty acid oxidation in MKRN1 KO MEFs and HepG2 cells depleted of MKRN1 (Fig. 1i-k, in line 141, on page 7). The possible reduction in lipogenesis could be induced by decreases in the levels of SREBP-1 and CHREBP in MKRN1 KO MEFs (Fig. 1e, in line 129 on page 7).

There was also an increase of PGC1α in MKRN1 KO MEFs, suggesting that the biogenesis of mitochondria is increased (Fig. 1e).

The metabolic pathways occurring in MEFs lacking MKRN1 were very similar to those occurring in the adipose tissue of the MKRN1 KO mice. For example, the increase of PGC1α was confirmed through RNA-seq analyses in adipose tissues from MKRN1 KO mice compared with WT mice (Fig. 5). Furthermore, the data indicated enrichment of genes in the glucose transporter pathway, glycolysis and TCA cycle as well as oxidative phosphorylation. On the other hand, lipid production was significantly suppressed. These findings are presented in the Results and Discussion sections (in line 254, on page 12).

2. In order to understand the physiological significance of the findings it would be interesting to know whether the expression of the ubiquitin ligase MKRN1 is elevated with obesity and diabetes.

Answer:

We checked the levels of MKRN in the obese and normal mice using qRT. The levels of MKRN1 did not change upon the induction of obesity and diabetes (Supplementary Fig. 6g, in line 211, on page 10).

3. Figure 6 Authors have found that liver lipids are lower in mice treated with the liver directed shRNA to MKRN1. Body mass and adiposity of these mice should be reported. Similarly it would be important to measure liver lipogenesis under these conditions as previous studies have indicated this is the primary mechanism by which increases in AMPK reduces liver lipid content (Fullerton et al. Nat Med 2013).

Answer:

This was also pointed out by the other two reviewers. We performed these experiments again to address these points. Briefly, there was no difference in body mass and adiposity in the obese mice injected with adenovirus carrying sh-MKRN1, as shown in Fig. 6e and 6j (in line 353, on page 16). We also measured the mRNA levels of SREBP and ChREBP, which are major factors in lipogenesis, and found that these two factors were decreased in the livers of the obese mice injected with adenovirus carrying sh-MKRN1 (Fig. 6h, in line 357, on page 16).

Minor Comments:

1. The manuscript is full of grammatical errors that must be corrected.

Answer:

We have had the grammatical errors corrected by an editing company.

2. There is a large number of reviews cited some of which are quite dated. It would be best for authors to use primary research articles to support claims and more recent reviews in select cases.

Answer:

We have updated the reviews as suggested.

1. Qi, J.*, et al.* Downregulation of AMP-activated protein kinase by Cidea-mediated ubiquitination and degradation in brown adipose tissue. *The EMBO journal* **27**, 1537-1548 (2008).
2. Barnes, K.*, et al.* Activation of GLUT1 by metabolic and osmotic stress: potential involvement of AMP-activated protein kinase (AMPK). *Journal of cell science* **115**, 2433-2442 (2002).
3. Marsin, A.S.*, et al.* Phosphorylation and activation of heart PFK-2 by AMPK has a role in the stimulation of glycolysis during ischaemia. *Current biology : CB* **10**, 1247-1255 (2000).
4. Chen, S.*, et al.* Complementary regulation of TBC1D1 and AS160 by growth factors, insulin and AMPK activators. *The Biochemical journal* **409**,

449-459 (2008).

5. McGee, S.L., *et al.* AMP-activated protein kinase regulates GLUT4 transcription by phosphorylating histone deacetylase 5. *Diabetes* **57**, 860-867 (2008).

REVIEWERS' COMMENTS:

Reviewer #1 (Remarks to the Author):

The authors have adequately addressed the concerns I have raised regarding both results and interpretation of the results. The manuscript has gained value with the inclusion of the new data, especially RNA sequencing data.

Reviewer #2 (Remarks to the Author):

The authors have addressed all my concerns and i have no further comments.

Reviewer #3 (Remarks to the Author):

The authors have addressed many of my concerns. However, with respect to liver lipogenesis they have not measured this directly. Therefore comments such as those found on line 410 " Although lipogenesis was consistently down-regulated in both the liver and adipose tissue" are incorrect and need to be modified and a line should be inserted stating this is a limitation of hte current manuscript.

Furthermore, it seems authors are discounting the possibility that the majority of their phenotype could be explained by increased AMPK phosphorylation of ACC (and decreased ACC activity and malonyl-CoA) rather than reductions in lipogenic gene expression as previously observed (PMID:24185692). This should be mentioned in the discussion.

Answers to the reviewers' comments

REVIEWERS' COMMENTS:

Reviewer #1 (Remarks to the Author):

The authors have adequately addressed the concerns I have raised regarding both results and interpretation of the results. The manuscript has gained value with the inclusion of the new data, especially RNA sequencing data.

Answer: No comment

Reviewer #2 (Remarks to the Author):

The authors have addressed all my concerns and i have no further comments.

Answer: No comment

Reviewer #3 (Remarks to the Author):

Question:

The authors have addressed many of my concerns. However, with respect to liver lipogenesis they have not measured this directly. Therefore comments such as those found on line 410 " Although lipogenesis was consistently down-regulated in both the liver and adipose tissue" are incorrect and need to be modified and a line should be inserted stating this is a limitation of the current manuscript.

Answer:

We corrected and added the following sentences in the result section in accordance with the reviewer's request.

Although **genes related to lipogenesis** were down-regulated consistently in both liver and adipose tissue, catabolism of fatty acid and glucose were up-regulated in adipose tissue, but down-regulated in the liver indicating different regulation of metabolic pathway in MKRN1 null mice: see main text, in line 409 on the manuscript page 18.

Importantly, further experiments including direct measurements of the lipid components in the liver should be carried out to reach explicit conclusions about the reduction of actual biochemical outcomes derived from comprehensive changes of the lipogenic genes: see main text, in line 413 on the manuscript page 18.

Question:

Furthermore, it seems authors are discounting the possibility that the majority of their phenotype could be explained by increased AMPK phosphorylation of ACC (and decreased ACC activity and malonyl-CoA) rather than reductions in lipogenic gene expression as previously observed (PMID:24185692). This should be mentioned in the discussion.

Answer:

We added the following sentences in the discussion section in accordance with the reviewer's request.

Of note most of the phenotypes observed in the MKRN1-null mouse could also be driven by AMPK-mediated phosphorylation of ACC, which suppresses its function. Thus, we cannot exclude the possibility that this process might have considerable negative effects on the accumulation of lipids in MKRN1-null mice, in addition to the downregulation of lipogenic gene expression: see main text, in line 415 on the manuscript page 18.
